# The conundrum of current endodontic disinfection strategies in microbial load reduction: a scoping review

**DOI:** 10.1186/s12903-026-07952-0

**Published:** 2026-03-18

**Authors:** Shymaa Shaaban, Abdelrahman Thabet, Semha Elsayed Elnaggar, Farah Tarek Barakat, Rana Hegazi, Hams Abdelrahman, Rania Elbackly, Hisham Elnawam

**Affiliations:** 1 Endodontics, Conservative Dentistry Department, Faculty of Dentistry, Alexandria University, Alexandria, Egypt; 2 Tissue Engineering Laboratories, Faculty of Dentistry, Alexandria University, Alexandria, Egypt; 3 Department of Endodontics, Faculty of Dentistry, Pharos University, Alexandria, Egypt; 4 Department of Pediatric Dentistry and Dental Public Health, Faculty of Dentistry, Alexandria University, Alexandria, Egypt

**Keywords:** Root canal disinfection, Endodontic Infection, Microbial reduction, Root canal irrigation, Intracanal medication, Culture-based microbial detection, Molecular methods of microbial detection

## Abstract

**Background:**

Effective disinfection is a diligent step in the treatment of endodontic infections to reduce the microbial load below a critical level that promotes healing of apical periodontitis (AP). Despite its clinical importance, there is no consensus on the most effective disinfection strategy to achieve this goal. Therefore, this scoping review aimed to comprehensively review the role of current disinfection therapies in microbial reduction during root canal treatment (RCT).

**Methods:**

A systematic search was conducted in PubMed, Web of Science, Scopus, and Google Scholar. Relevant clinical studies were selected based on predefined inclusion criteria. Data extraction focused on microbial load reduction, disinfection protocols, and microbial detection techniques.

**Results:**

Out of 3519 retrieved articles, 148 were eligible and processed for data extraction. All included studies were clinical studies. Most of the studies were primary RCT (82.43%) and secondary RCT (15.54%). While only 3 studies (2.03%) included both primary and secondary RCT. Microbial load reduction was assessed in the majority of the studies, while few others assessed endotoxin reduction. The mean percentage of microbial load reduction was (87.24 ± 21.86). The most frequently used disinfection regimens were irrigation with; (2.5% NaOCl alone, 2.5% NaOCl followed by 17% EDTA, 2%CHX; 16.05%, 11.11%, 10.49% respectively), activation methods including; passive ultrasonic irrigation (PUI); 12.35%, photodynamic therapy (PDT); 11.11%, and laser; 11.11%) and intracanal medication (ICM) with Ca (OH)2, Ca (OH)2 + 2%CHX, or 2%CHX; 31.48%, 10.51%, 7.41% respectively. The most prevalent methods of microbial detection were culture-based (64.86%), and molecular-based methods were performed in 58.10% of the studies.

**Conclusion:**

A combination of advanced irrigation techniques, appropriate ICM, and adjunctive disinfection activation techniques provides the most effective approach for both microbial and endotoxin reduction. However, complete disinfection and its influence on the validity of treatment outcomes remain a dilemma. The healing of apical periodontitis is multifactorial, and future research should focus on personalized treatment protocols based on patient and tooth specific considerations to optimize clinical outcomes.

**Supplementary Information:**

The online version contains supplementary material available at 10.1186/s12903-026-07952-0.

## Background

The success of root canal treatment (RCT) largely relies on the thorough eradication of microorganisms from the root canal system or their reduction to a subcritical level that allows periapical healing [1]. However, this objective is difficult to achieve due to various considerations, including anatomical complexity, microbial load, and the resilience of microbial biofilms [2, 3]. In the framework of endodontic treatment, these factors may adversely impact the treatment outcomes [4].

Apical periodontitis (AP) is primarily driven by persistent microbial infection within the root canal system [5]. The relationship between microbial levels and the development of AP highlights the clinical significance of effective disinfection strategies that can reduce the microbial load to a level that may effectively promote periapical healing or even prevent disease progression [1, 5, 6].

A solid understanding of the microbiological nature of endodontic infections is crucial for achieving successful endodontic treatment. Root canal infections are primarily polymicrobial [7], with anaerobic and facultative anaerobic bacteria being the most observed [5, 8]. Frequently, these microorganisms organize into biofilms that firmly attach to the canal walls, penetrate dentinal tubules, and exhibit resistance to disinfection agents [9]. As a result, the emergence of these structured organizations contributes to the persistence of infection and subsequently increases the likelihood of treatment failure [10].

Current disinfection protocols typically involve a combination of mechanical instrumentation, irrigation solutions, and intracanal medicaments (ICM) to reduce microbial load within the root canal system [11–13]. Sodium hypochlorite (NaOCl) has been widely acknowledged as the primary irrigant in root canal treatment [14, 15], often accompanied with adjunctive agents such as chlorhexidine (CHX) and ethylenediaminetetraacetic acid (EDTA) to enhance antimicrobial activity and aid in smear layer removal [16]. However, because irrigants have limited residual activity, intracanal medicaments, chiefly calcium hydroxide Ca(OH)₂, are applied between appointments to promote disinfection and eradicate remaining microorganisms [13]. Besides conventional chemical methods, literature has progressively explored a range of approaches to enhance disinfection efficacy. Among these are the use of nanoparticles, photodynamic treatment, and sonic or ultrasonic activation devices [17–21].

Additionally, there is an increase in scientific interest that encourages the use of natural disinfection agents to serve as potential alternative or complementary irrigants [22–24]. Although these methods have shown promising antimicrobial effects, limitations continue to impede clinical success [13].

Although several studies have assessed the efficacy of disinfection methods by quantifying microbial reduction, there is no agreement on the most potent disinfection approach that correlates with the eventual healing of AP [5, 6]. Accordingly, constant research and technological advancements continue to refine disinfection protocols.

In the analysis of microbial pathogens, different microbiological methods have been used, each with its own advantages and limitations. However, the selection of the appropriate method may considerably affect the detection and identification of specific microorganisms, which in turn may complicate the interpretation of findings across studies [25]. The consistency of findings is challenged due to heterogeneity in study designs, disinfection protocols, outcome assessment methods, and the lack of a clear correlation between the microbial threshold levels and the predictability of healing [5]. Consequently, this scoping review aimed to map the existing evidence regarding the role of disinfection strategies in microbial reduction during endodontic treatment and offer insight into optimal approaches of antimicrobial therapy.

### Methodology

The guidelines of the Preferred Reporting Items for Systematic reviews and Meta-Analyses extension for Scoping Reviews (PRISMA-ScR) [26] were followed to answer the following research question/objective: How effective are root canal disinfection therapies in reducing the microbial load in endodontic infections? `.

This scoping review followed a PCC acronym, where P (Population) represents patients undergoing non-surgical root canal treatment/retreatment in mature permanent teeth, C (Concept) represents microbial load reduction, and C (Context) represents root canal disinfection therapies.

### Search strategy

An electronic search was conducted in PubMed, Scopus, Web of Science, and Google Scholar databases, searched from inception to March 2025. The full search strategy is presented in Table 1.

### Eligibility criteria

This scoping review involved clinical studies in the English language with no restriction on age, sex, or time, conducted on mature permanent teeth with endodontic infections that were treated by primary and/or secondary root canal treatment, evaluating the antimicrobial effect of root canal disinfection therapy in-situ and reporting microbial reduction as an outcome. Accepted study designs included randomized controlled trials, cohort studies, and case-control studies. On the other hand, case reports, case series, reviews, pilot studies, preliminary reports, animal studies, in vitro studies, and studies involving surgical root canal retreatment were excluded. Moreover, studies that did not evaluate the antibacterial effectiveness as a primary outcome or involved unhealthy populations were also excluded.

### Identifying relevant studies

An electronic de-duplication method was implemented using EndNote X9 citation management system (Clarivate, Philadelphia, PA). All articles were exported to a Microsoft Excel spreadsheet. Pre-screening calibration exercises were performed by 5 reviewers (S.S., A.T., S.E.E., F.T.B. and R.H). A pilot screening of a random sample of records was independently conducted by the same reviewers. Any discrepancies were discussed to ensure consistency in the interpretation of inclusion and exclusion criteria.

Subsequently, the titles and abstracts of all records were screened independently by 5 reviewers (S.S., A.T., S.E.E., F.T.B. and R.H). Comprehensive full-text reading of the potentially eligible studies was carried out by the same evaluators to assess their eligibility. Disagreements between reviewers at either the title/abstract or full-text screening stages were initially addressed through discussion among the 5 reviewers. When consensus could not be reached, the disputed article was sent to two senior reviewers (R.E. and H.E.) for consultation. Only studies that met all eligibility criteria were included and processed for data extraction.

### Data collection and analysis

The data extraction was piloted by all reviewers on a small sample of the included studies to define the data to be extracted. Five reviewers (S.S., A.T., S.E.E., F.T.B. and R.H) independently extracted data from all the included studies using pre-designed data extraction tables. To ensure consistency across the five extractors, dual verification was performed so that data from every included study was independently extracted by one primary extractor, and a second extractor independently verified the extracted data for accuracy and completeness. Any disagreements were resolved through discussion, with the senior reviewers (R.E. and H.E.) acting as a final arbiter if needed. The following data were extracted: author, year, study design, sample size, patient characteristics, general findings (supplementary Table 1), geographic location, tooth type, groups, type of root canal treatment (RCT) (primary or secondary), apical size preparation, number of visits, type of disinfection therapy evaluated, tooth diagnosis, method of assessing the outcome, bacterial endotoxin detection, significant antibacterial reduction, percentage of antibacterial or endotoxin reduction (if present), and microbiological sampling of root canals was also recorded including: number and timing of bacteriologic samples, sampling collection source, sampling technique and sterility control sample (Supplementary Table 2). Descriptive statistics were performed using Microsoft Excel 2016 and IBM SPSS Statistics version 23 (Armonk, NY, USA). Frequencies and percentages of the included studies were calculated. The overall percentage reduction in toxin and microbial loads was calculated using the reported pre- and post-intervention values. When outcomes were reported as medians with minimum and maximum values, they were converted to means and standard deviations prior to analysis.

**Table 1:** Search Strategy employed

Data Base	Search Terms
Web of science	((((((((((((((((((((((ALL=(“Periapical Periodontitis”)) OR ALL=(“endodontic infection”)) OR ALL=(“residual infection”)) OR ALL=(“persistent root canal infection”)) OR ALL=(“post treatment apical periodontitis”)) OR ALL=(“secondary root canal infection”)) OR ALL=(endodontic retreatment))) OR ALL=(persistent endodontic infection))) OR ALL=(“Tooth, Nonvital”) OR ALL=(“root canal treat*”)) OR ALL=(“root canal therap*”)) OR ALL=(“endodontic treat*”)) OR ALL=(“pulpless t*”)) OR ALL=(“root-filled”)) OR ALL=(“Periapical heal*”)) OR ALL=(“periapical status”)) OR ALL=(“periapical pathosis”)) OR ALL=(“apical periodontitis”)) OR ALL=(“periapical radiolucency”)) OR ALL=(“periapical breakdown”)) OR ALL=(failed primary root canal treatment))) OR ALL=(secondary root canal treatment))) OR ALL=(refractory) AND (((((((((((ALL=(“Anti-Infective Agents”)) OR ALL=(“Anti-Infective Agents, Local”)) OR ALL=(“root canal medicament”)) OR ALL=(antibiofilm)) OR ALL=(“intracanal disinfection”)) OR ALL=(root canal disinfection))) OR ALL=(root canal antimicrobial therapy))) OR ALL=(antimicrobial)) OR ALL=(antibacterial)) OR ALL=(endodontic disinfection agents))) OR ALL=(root canal irrigation))) OR ALL=(intracanal medication))) OR ALL=(root canal medication)) AND (((((((((((ALL=((intracanal microbial load reduction))) OR ALL=((microbial load reduction))) OR ALL=((intracanal bacterial count reduction))) OR ALL=((root canal bacterial count reduction))) OR ALL=((root canal bacterial load reduction))) OR ALL=((intracanal bacterial load reduction))) OR ALL=((microbiome reduction))) OR ALL=((polymerase chain reaction))) OR ALL=(CFU)) OR ALL=((colony forming unit)) OR ALL=((microbial detection))) OR ALL=((molecular assay))
Scopus	root canal bacterial count OR root canal bacterial load OR microbial detection OR intracanal bacterial count AND root canal medicaments OR root canal disinfection OR root canal antimicrobial therapy OR endodontic disinfection agents OR root canal irrigation OR root canal medication OR root canal antibacterial AND periapical periodontitis OR endodontic infection OR residual infection OR persistent root canal infection OR post treatment apical periodontitis OR secondary root canal infection OR endodontic retreatment OR persistent endodontic infection
PubMed	“Periapical Periodontitis“[mh] OR “endodontic infection” OR “residual infection” OR “persistent root canal infection” OR “symptomatic apical periodontitis” OR “asymptomatic apical periodontitis” OR “post treatment apical periodontitis” OR “secondary root canal infection” OR (endodontic retreatment) OR (persistent endodontic infection) OR “Tooth, Nonvital”[mh] OR “root canal treat*” OR “root canal therap*”[mh] OR “endodontic treat*” OR “pulpless t*” OR “root-filled” OR “Periapical heal*” OR “periapical status” OR “periapical pathosis” OR “apical periodontitis” OR “periapical radiolucency” OR “periapical breakdown” OR (failed primary root canal treatment) OR (secondary root canal treatment) OR refractory AND “Anti-Infective Agents“[mh] OR “Anti-Infective Agents, Local“[mh] OR “root canal medicament” OR “intracanal disinfection” OR (root canal disinfection) OR (root canal antimicrobial therapy) OR antimicrobial OR antibacterial OR antibiofilm OR (endodontic disinfection agents) OR (root canal irrigation) OR (intracanal medication) OR (root canal medication) AND (intracanal microbial load reduction) OR (root canal microbial load reduction) OR (intracanal bacterial count reduction) OR (root canal bacterial count reduction) OR (root canal bacterial load reduction) OR (intracanal bacterial load reduction) OR (microbial load reduction) OR (microbiome reduction) OR PCR OR (polymerase chain reaction) OR CFU OR (colony forming unit) OR (microbial detection) OR (molecular assay)
Google scholar	root canal disinfection agents AND endodontic infection AND intracanal microbial load reduction AND clinical studies

## Results

### Selection of sources of evidence

**Fig. 1:**
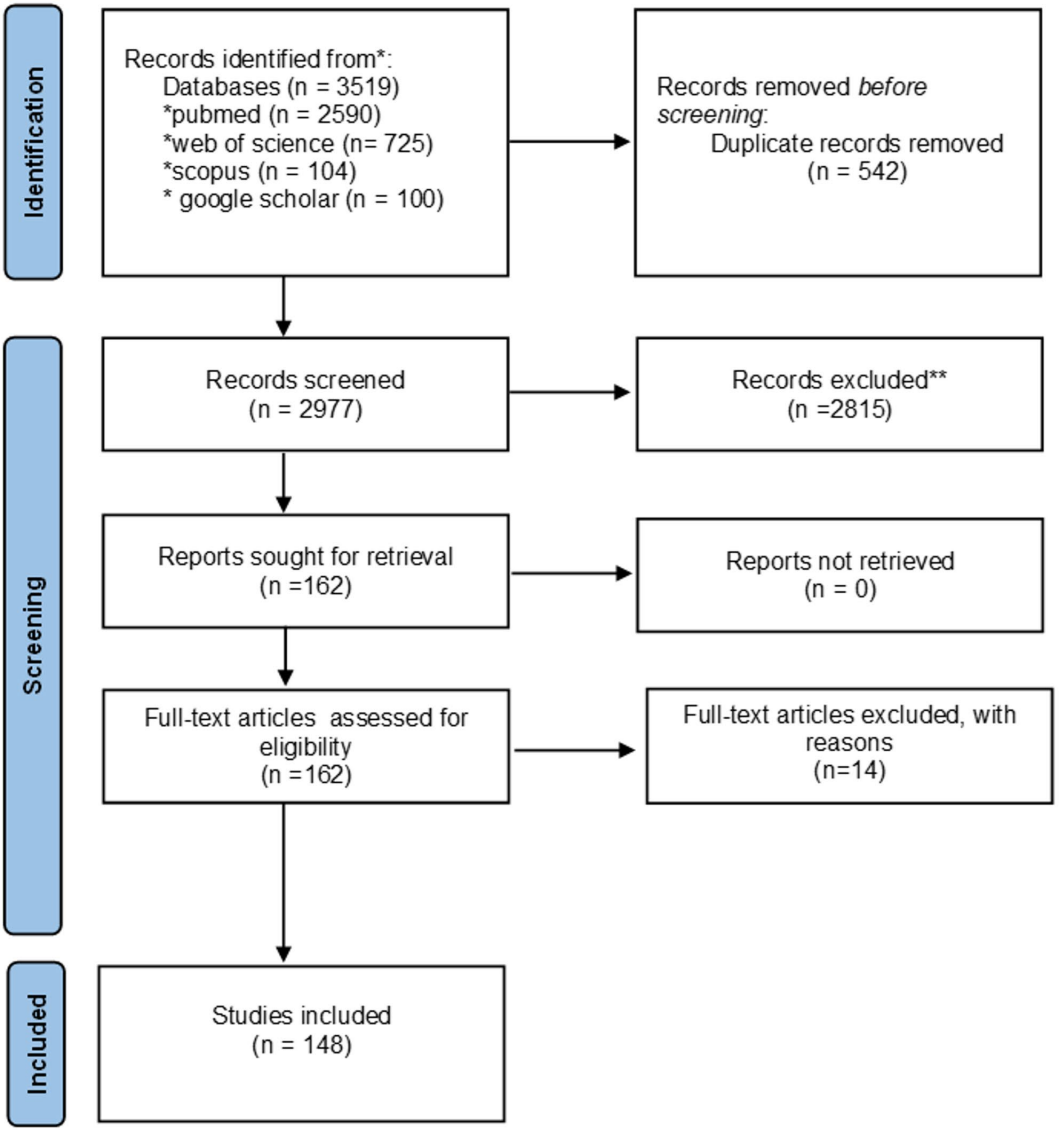
PRISMA Scr flowchart of the reviewing process

An initial 3519 records were retrieved from online databases search (PubMed = 2590, Scopus = 104, Web of Science = 725, Google Scholar = 100 [top 100 relevant studies). After duplicates removal, 2977 records were retained for screening. An initial screening resulted in exclusion of 2815 records based on titles and abstracts. A further 14 records were excluded following the full text screening, supplementary Table 3. Eventually, 148 studies met the inclusion criteria and were processed for data extraction and synthesis. The reviewing process is shown in the PRISMA Scr flowchart Figure 1 [26].

### Global distribution of the included studies

**Fig. 2:**
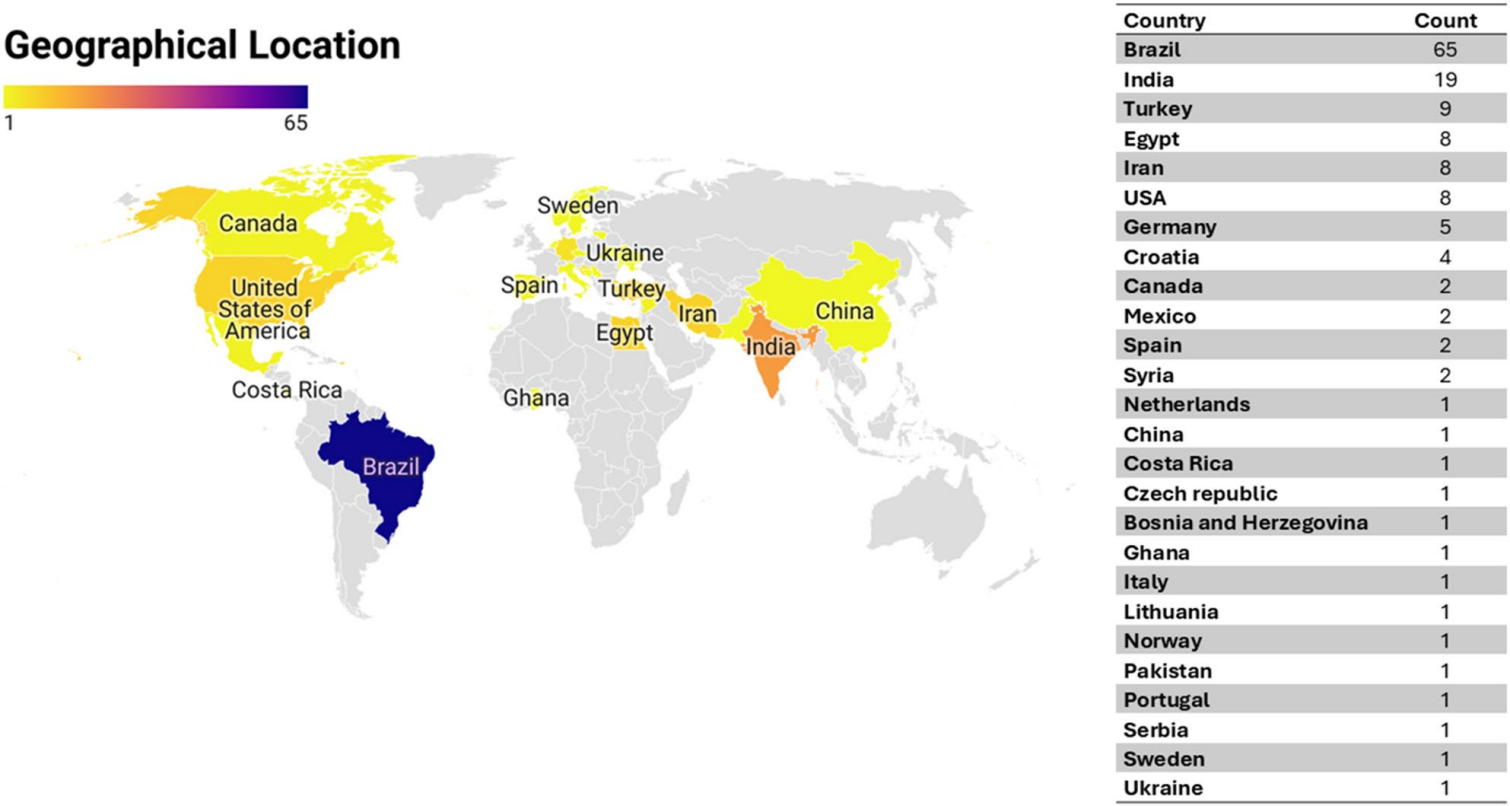
World map of the studies included in the scoping review

Concerning publishing countries, Brazil was found to have the highest total number of published articles (65 studies) [21, 27–90]. While most of the other studies were done in India [11, 15, 91–107], Turkey [12, 108–115], Egypt [116–123] Iran [20, 124–130] and USA [131–138] with a total number of published articles (19,9,8,8, and 8, respectively). Other studies were conducted in; Germany [139–143] (5 studies), Croatia [144–147] (4 studies), Canada [148, 149] (2 studies), Mexico [150, 151] (2 studies), Spain [152, 153] (2 studies), Syria [154, 155] (2 studies), Netherlands [156] (1 study), China [157] (1 study), Costa Rica [158] (1 study), Czech republic [159] (1 study), Bosnia and Herzegovina [160] (1 study), Ghana [161] (1 study), Italy [162] (1 study), Lithuania [163] (1 study), Norway [164] (1 study), Pakistan [165] (1 study), Portugal [166] (1 study), Serbia [167] (1 study), Sweden [168] (1 study) and Ukraine [169] (1 study). A world map of the global distribution of the included studies is shown in Figure 2.

### Time distribution of the included studies

**Fig. 3:**
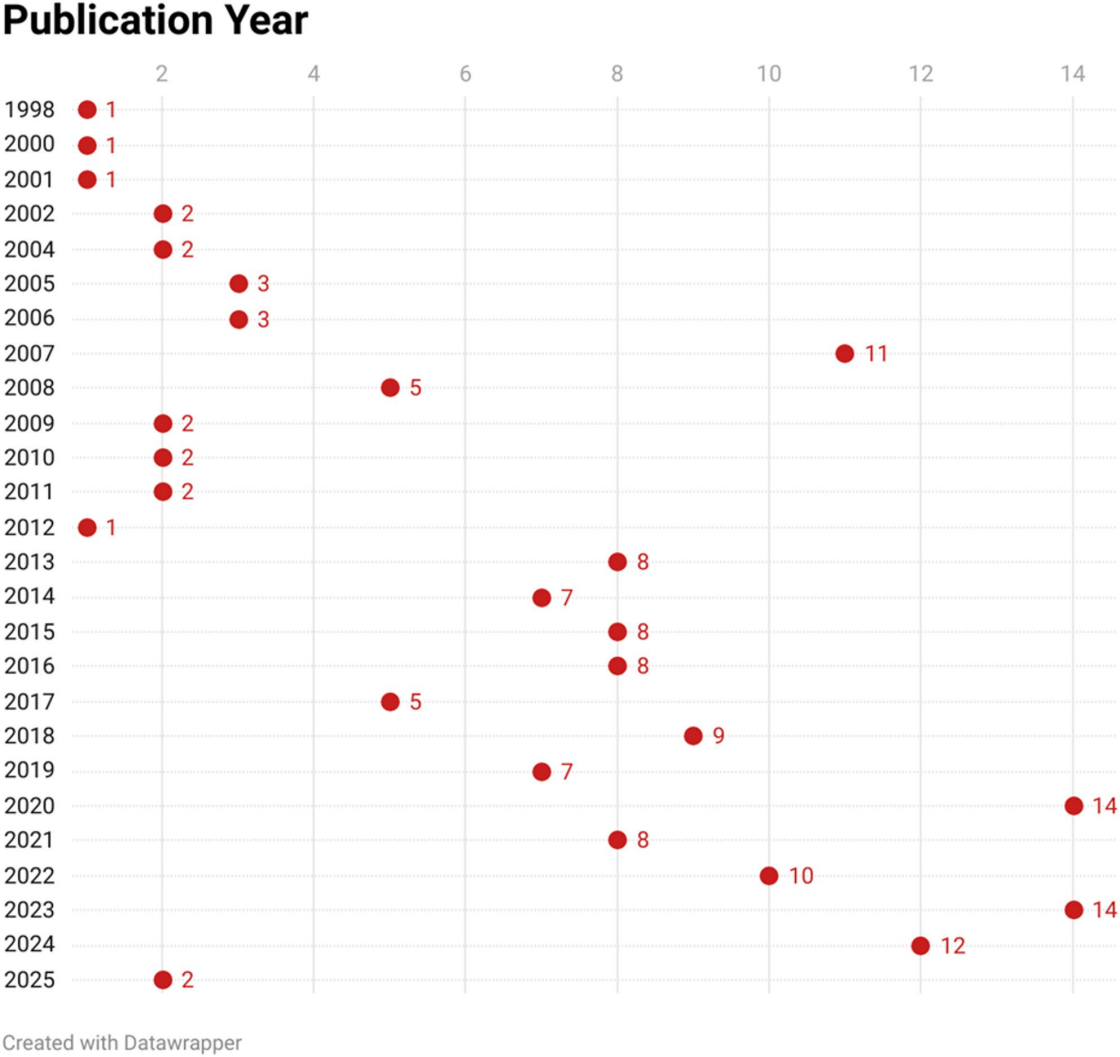
Time distribution by year of the studies included in this scoping review (Red dots represent number of studies)

The number of articles published generally increased over time. Sixty of the 148 included studies were conducted between 2020 and 2025. This indicates a growing interest in and expansion of the research field of disinfection efficacy of irrigants, as shown in Fig. 3.

### General characteristics of the included studies

Table 2 presents general details and characteristics of the included studies. All the studies included in the present review were clinical studies. One hundred and twenty-two (82.43%) studies included primary RCT, 23 (15.54%) studies included secondary RCT, while only 3 studies (2.03%) included both primary and secondary RCT [142, 146, 165]. Among the 148 included studies, 50 were identified as randomized controlled trials (RCTs) (33.8%). Eighty studies (54.1%) did not clearly specify their study design, although they were conducted as interventional clinical investigations with defined treatment protocols and outcome assessments. The remaining 18 studies (12.2%) were non-randomized clinical trials, including five described as cross-sectional and one as a cohort study. The included tooth type differed between the studies, ranging from single rooted teeth in 92 (62.16%) studies, followed by single and multi-rooted teeth in 34 (22.97%) studies, followed by multi-rooted teeth in 9 (6.08%) studies. On the other hand, 13 (8.78%) studies did not mention the tooth type.

There were variable diagnoses across the studies. Moreover, some studies included more than one diagnosis. However, the most prevalent one was pulp necrosis (PN) with apical periodontitis (AP) in 75 (50.68%) studies, AP in 20 (13.51%) studies, PN alone in 17 (11.49%) studies and previously treated (PT) with AP in 15 (10.14%) studies. It is worth mentioning that some studies only included irreversible pulpitis in the clinical trials, which was present in 5 (3.38%) studies [34, 37, 98, 139, 140].

In most of these clinical studies, root canal treatment was done in two visits (98; 66.22%), followed by single visit in 32 studies (21.62%), and three visits in 5 studies (3.38%). Moreover, some studies didn’t mention a specific number of visits. Furthermore, apical size of the preparation was also recorded i.e. the largest preparation size reached. Studies often used more than 1 apical preparation size. However, some papers (16.67%) did not mention the apical preparation size. The most used preparation size was ISO size 40 in 56 studies (21.71%), ISO size 50 in 37 studies (14.34%), and ISO size 30 in 29 studies (11.24%). The smallest reported ISO size was 25 in 23 studies (8.91%). On the contrary, the largest reported ISO size was 80, which was reported only in 1 study (0.39%). In the secondary treatment, the use of small ISO size 25 was reported in two studies [127, 145].

**Table 2:** General characteristics of the included studies

Parameters		Total Number of studies = 148 (Frequency (%))
Type of clinical trial	Primary	122 (82.43%)
	Secondary	23 (15.54%)
	Both primary and secondary	3 (2.03%)
Tooth type	Single-rooted	92 (62.16%)
	Single and multi-rooted	34 (22.97%)
	Multi-rooted	9 (6.08%)
	Not mentioned	13 (8.78%)
Tooth diagnosis	PN with AP	75 (50.68%)
	AP	20 (13.51%)
	PN	17 (11.49%)
	PT with AP	15 (10.14%)
	PT	5 (3.38%)
	IP	5 (3.38%)
	PN with AP, IP	3 (2.03%)
	Vital pulp and associated periodontal disease	1 (0.68%)
	PN only, PN with AP	1 (0.68%)
	PN, AP, IP	1 (0.68%)
	IP, AP, or trauma	1 (0.68%)
	PN with AA/AP	1 (0.68%)
	PN with AP/AAA	1 (0.68%)
	Generalized periodontitis	1 (0.68%)
	PN with/without AP	1 (0.68%)
Number of visits	One	32 (21.62%)
	Two	98 (66.22%)
	Three	5 (3.38%)
	One – Two	5 (3.38%)
	Two – Three	1 (0.68%)
	Not mentioned	7 (4.73%)
Apical size	25	23 (8.91%)
	30	29 (11.24%)
	35	15 (5.81%)
	37	1 (0.39%)
	40	56 (21.71%)
	45	12 (4.65%)
	50	37 (14.34%)
	55	16 (6.20%)
	60	20 (7.75%)
	70	5 (1.94%)
	80	1 (0.39%)
	Not mentioned	43 (16.67%)

*PN* Pulp Necrosis, *AP* Apical Periodontitis, *PT* Previously Treated, *IP* Irreversible Pulpitis, *AAA* Acute Apical Abscess, *AA* Apical Abscess

### Intervention and outcome characteristics of the included studies

Out of the 148 included trials, microbial load reduction was assessed quantitively in most of the studies. Twenty-one studies assessed endotoxin reduction besides microbial load reduction. Conversely, 2 studies assessed endotoxin reduction only [76, 129].

### Method of assessment of outcome

**Fig. 4:**
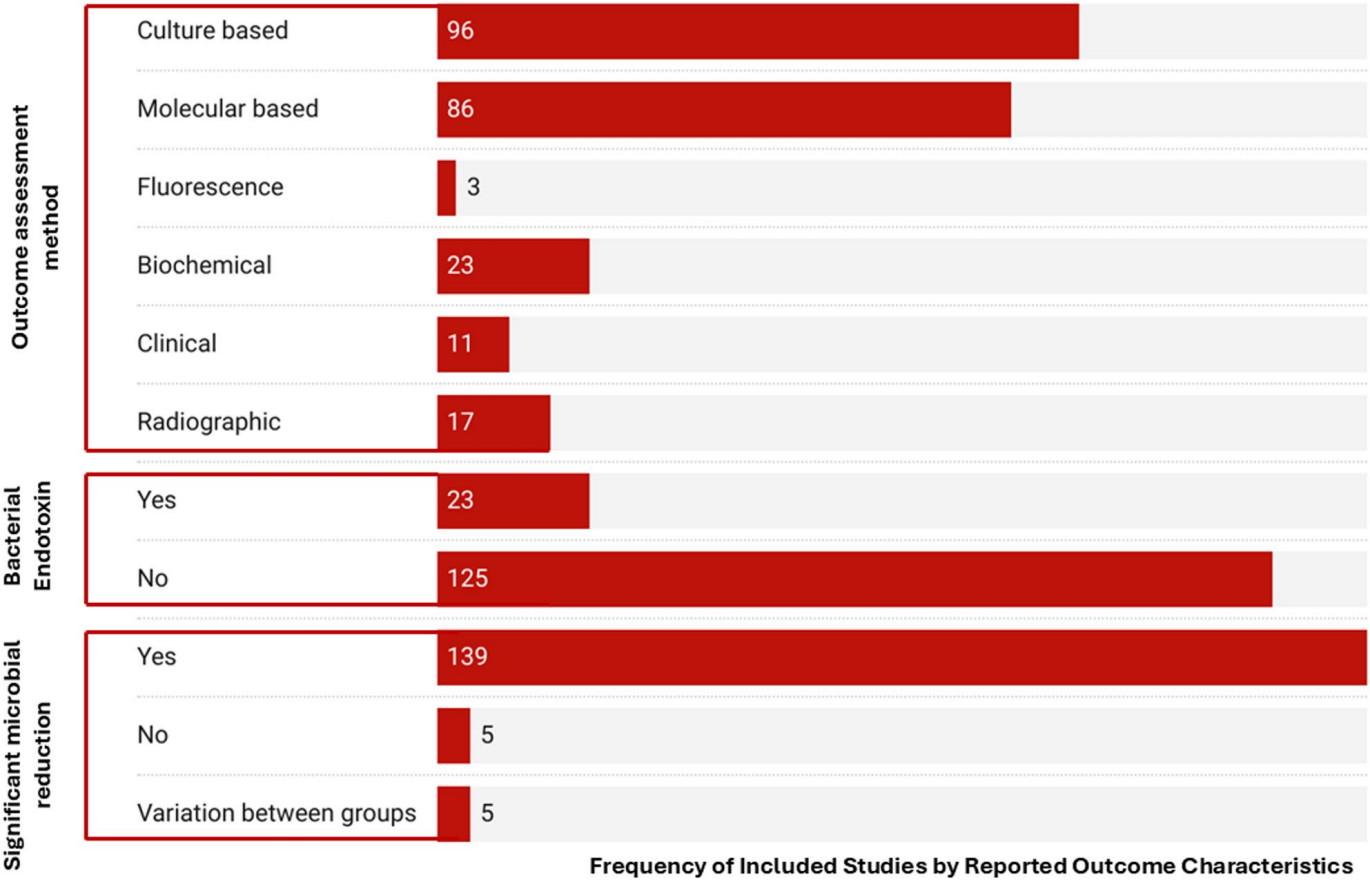
Outcome characteristics of the included studies

A multi-methodological assessment approach was adopted in several studies. The most prevalent assessment techniques were culture-based and molecular-based methods, utilized in 94.86% (*n* = 96) and 58.10% (*n* = 86) of the studies, respectively. Other modalities were less frequent, including biochemical assays (15.54%), radiographic assessment (11.49%), clinical evaluation (7.43%), and fluorescence-based techniques (2.03%). Out of all studies, 139 studies (93.92%) reported significant anti-microbial reduction outcome. On the other hand, 5 (3.38%) studies did not report significant antimicrobial reduction, as shown in Fig. 4.

### Disinfection protocols and microbial reduction

*Disinfection strategies*: The disinfection strategies differed greatly among the included studies, as shown in Table 3. The most used irrigation solutions were: [2.5% NaOCl (16.05%)], [2.5% NaOCl + 17% EDTA (11.11%)], [2% CHX (10.49%)], [5.25% NaOCl (8.64%)], or [6% NaOCl (7.41%)]. Natural irrigants were only mentioned in two studies: garlic-extract [101] and Calendula officinalis (C. officinalis) [100]. Regarding irrigant activation, passive ultrasonic irrigation (PUI) (12.35%), photodynamic therapy (PDT) (11.11%) and laser activated irrigation (LAI) (11,11%) were the most used methods, as shown in Table 4. As for ICM, out of 148 studies; 51 studies (31.48%) used Ca(OH)_2_ ICM, 17 studies (10.51%) used Ca(OH)₂ + 2%CHX ICM, and 12 studies (7.41%) used 2%CHX as an ICM, as shown in Table 5. Only 4 natural ICMs were used in the included studies: Bromelain Paste [96], garlic extract [120], glycyrrhizin [117], and chitosan (CS) [104].

Microbial load reduction: The mean percentage of microbial load reduction of studies addressing microbial load was (87.24 ± 21.86%).

**Table 3:** The most common irrigation solutions (≥ 2 studies) used in included studies, addressing microbial load from highest to lowest frequency %

Irrigation	Frequency	%
2.5% NaOCl	26	16.05
2.5% NaOCl, 17% EDTA	18	11.11
2% CHX	17	10.49
5.25% NaOCl	14	8.64
6% NaOCl	12	7.41
Saline	7	4.32
1% NaOCl	5	3.09
0.12% CHX	5	3.09
5.25% NaOCl, EDTA	4	2.47
3% NaOCl	3	1.85
0.5% NaOCl	3	1.85
2% CHX, 17% EDTA	3	1.85
NaOCl + Dual Rinse HEDP	2	1.23
2% CHX, 17% EDTA, saline	2	1.23
2% NaOCl	2	1.23
2% IKI	2	1.23
2.5% NaOCl with 17% EDTA with final flush using 2% CHX	2	1.23
6% NaOCl and 17% EDTA	2	1.23
2.5% NaOCl, 17% EDTA, saline	2	1.23

*NaOCl* Sodium hypochlorite, *EDTA* Ethylenediaminetetraacetic acid, *CHX* Chlorhexidine, *HEDP* Etidronic acid (1-hydroxyethane 1,1-diphosphonic acid, *IKI* Iodine-potassium Iodide

**Table 4:** Types of activation methods used in the included studies, addressing microbial load from highest to lowest frequency

Activation methods	Frequency	%
PUI	20	12.35
PDT	18	11.11
LAI	18	11.11
CNI	5	3.09
EA	2	1.23
XPF	2	1.23
EndoVac system	2	1.23
automated irrigation device	1	0.62
Manual dynamic agitation	1	0.62
Depotphoresis	1	0.62
F-file activation	1	0.62
EC	1	0.62
IRRI S^®^ files	1	0.62
SAF	1	0.62
PP irrigation	1	0.62

*PUI* Passive Ultrasonic Irrigation, *PDT* Photodynamic Therapy, *LAI* Laser Activated Irrigation, *CNI* Conventional Needle Irrigation, *EA* Endo-Activator, *XPF* XP-Endo Finisher, *EC* Easy Clean tip reciprocating activation, *SAF* Self Adjusting File, *PP irrigation* Positive Pressure Irrigation

**Table 5:** The most common intracanal medications (≥ 2 studies) used in the included studies, addressing microbial load from highest to lowest frequency

Medications	Frequency	%
Ca(OH)2	51	31.48
Ca(OH)2 + 2%CHX	17	10.51
2%CHX	12	7.41
Ca(OH)2 in CPMC and glycerin (CHPG)	6	3.71
DAP	3	1.85
Ca(OH)2/glycerin	3	1.86
Cupral paste	3	1.86
TAP	3	1.86
Ledermix	2	1.23

*Ca*(OH)_2_ Calcium Hydroxide, *CHX* Chlorhexidine, *CPMC* Camphorated ParaMonochloroPhenol, *CHPG* Ca(OH)_2_ in CPMC and glycerin, *DAP* Double Antibiotic Paste, *TAP* Triple Antibiotic Paste

### Disinfection protocol and endotoxin reduction

#### General characteristics

**Fig. 5:**
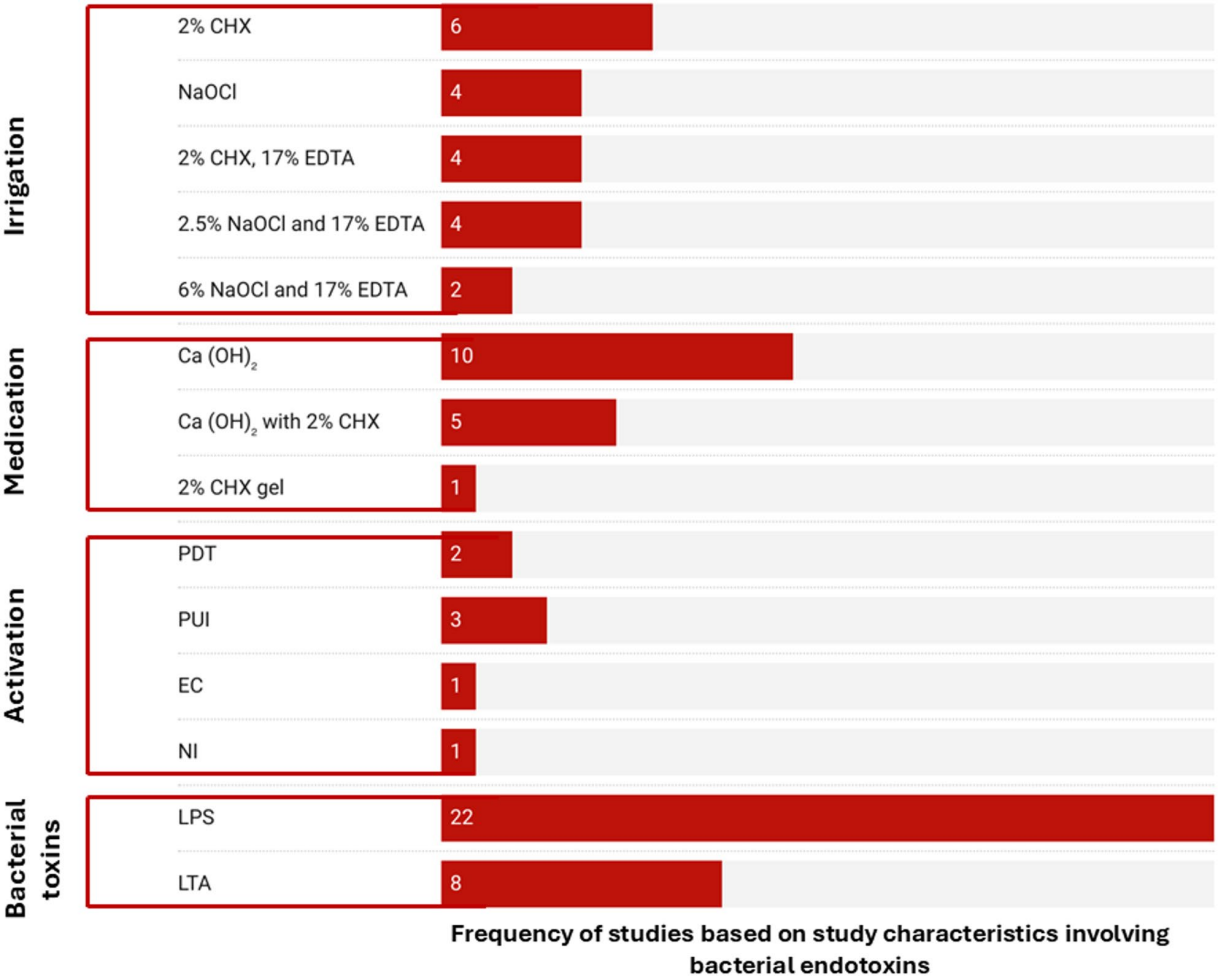
Characteristic of studies including bacterial endotoxins *CHX:* Chlorhexidine, *NaOCl:* Sodium hypochlorite, *EDTA:* Ethylenediaminetetraacetic acid,* Ca*(OH)_2_*:* Calcium Hydroxide, *PDT:* Photodynamic Therapy,* PUI:* Passive Ultrasonic Irrigation,* EC*: Easy Clean tip, *NI:* Needle Irrigation, *LPS: *lipopolysaccharides, *LTA:* Lipoteichoic acid

Endotoxin levels were evaluated in 23 of the included studies, with Lipopolysaccharides (LPS) serving as the primary biomarker in 95.7% (*n* = 22) of the studies. In contrast, Lipoteichoic acid (LTA) was reported in 34.8% (*n* = 8) of the studies.2% CHX irrigation (26.09%), PUI (13.04%), and Ca(OH)₂ ICM (43.48%) were the most common disinfection strategies used in the studies addressing endotoxin levels, as shown in Fig. 5.

#### Endotoxin reduction

Quantitative analysis revealed a significant disparity in endotoxin reduction efficiency based on the assessment marker; the mean reduction for LPS was (86.95 ± 16.40) %, whereas the mean reduction for LTA was substantially lower at (43.05 ± 11.89) %.

## Discussion

After root canal treatment, residual microbial entities may not prevent healing of apical periodontitis (AP), provided that the microbial load is reduced below a critical threshold that is controlled by the immune system and tissue repair processes [5]. This threshold can differ based on: the pathogenicity of bacterial species, the host’s immune capabilities, and the complexity of root canal anatomy [170]. It has been proposed that microbial levels under approximately 10³ CFU/mL may aid in achieving successful clinical and radiographic healing according to Siqueira et al. [170].

Indeed, despite the advancements in endodontic therapy and disinfection strategies, AP remains a clinical concern, particularly in cases with persistent lesions or delayed healing [171]. These outcomes highlight the importance of optimizing intracanal disinfection strategies to improve treatment success [172]. Current literature has revealed heterogeneity and diversity in disinfection protocols and methods of microbial assessment, leading to inconsistent findings and a lack of standardized guidelines [1, 173]. Considering this, the present scoping review aimed to thoroughly map current research on intracanal disinfection and how it influences microbial load reduction, which has a critical impact on the outcome of endodontic treatment.

This scoping review was executed following the PRISMA-ScR [26] to guarantee a systematic approach for data synthesis. One of the key strengths of the current review is that it focused exclusively on human clinical studies to support clinical relevance and ensure the applicability of the findings. However, case reports, case series, and preliminary studies were excluded as these designs lack generalizability [174–176]. Only English-language studies were included to prevent possible misinterpretation of data that could arise from translation. Studies that did not report microbial reduction as an outcome were excluded, as the main objective of this review is to evaluate the antimicrobial effect of root canal disinfection therapy in terms of in-situ microbial load reduction. As for the type of teeth, immature permanent teeth were excluded to avoid confounding variables associated with regenerative procedures and revascularization, which follow clinical pathways distinct from the conventional disinfection protocols applied to mature permanent teeth. Primary teeth were also excluded due to their anatomical differences from permanent teeth and because their inclusion was outside the scope of the present review [177, 178]. This selective inclusion represents a point of strength in this review as it improves the consistency of the findings.

Another significant strength of this review is that it included a large pool of data from different databases with no restriction on age, sex, country, or time. Accordingly, this scoping review provides a broad overview and could be considered as a comprehensive map of the available literature on endodontic disinfection strategies.

## Strategies for microbial reduction

### Conventional strategies

Although there are huge advancements in the techniques and materials used, conventional strategies remain indispensable due to their proven clinical effectiveness and crucial effect in controlling the microbial activity, which is essential for periapical healing and the long-term success of endodontic treatment [179].

The great majority of the 148 included studies used NaOCl as the main irrigant with various concentrations ranging from 0.5% to 6%, with different temperature values [179]. Our findings support the continued usage of NaOCl as a key component of endodontic disinfection and emphasize its impact as a root canal irrigant due to its broad-spectrum antimicrobial activity and tissue-dissolving capabilities [27, 180]. However, the variability in the utilized concentrations and combinations of NaOCl with other additions highlights a lack of consensus and the need to continue searching for more effective and comprehensive antimicrobial regimens [27–29].

The combination of EDTA and NaOCl is widely recognized as a standard procedure in endodontic irrigation for ensuring the enhancement of dentinal tubules’ permeability [1, 132]. Despite that, this combination was reported in only 11.11% of the included studies. The limited use of the EDTA and NaOCl combination in the reviewed studies may be due to variations in study focus, concerns over reduced NaOCl efficacy when used with EDTA, the chemical precipitate formed by irrigant interactions, and a growing interest in newer irrigation methods and agents. Additionally, many studies did not prioritize smear layer removal and were more concerned about the antibacterial effect of the used irrigants and chelating agents, which is the primary benefit of this combination, explaining its underrepresentation despite its clinical importance [1, 132].

In the present review, the disinfection efficacy of different concentrations of chlorhexidine (CHX) as an irrigant was evaluated, demonstrating a significant reduction in microbial load [134, 147]. One of its key advantages is its substantivity—its ability to adsorb onto dentin and exert a prolonged antimicrobial effect even after the irrigant is removed [148]. Additionally, CHX is effective against both gram-positive and gram-negative bacteria, as well as fungi such as *Candida albicans*, making it a valuable adjunct in endodontic disinfection, especially in cases where sodium hypochlorite cannot be used due to tissue toxicity concerns [148, 149]. Although CHX is considered an efficient disinfection agent and has demonstrated comparable results to NaOCl, its use as an additional irrigant following irrigation with NaOCl and EDTA has been questioned [143]. Specifically, one study reported that the adjunctive use of CHX did not have a statistically significant reduction in microbial load beyond that which was achieved with thorough irrigation using NaOCl and EDTA alone [143]. This suggests that, under conditions of copious and effective irrigation with NaOCl and EDTA, the subsequent application of CHX may be redundant and provide minimal additional antimicrobial benefit [143].

Although iodine potassium iodide (IKI) has long been considered a traditional endodontic irrigant, its clinical relevance has resurfaced in recent literature [130, 154, 163]. A 2025 study explored the renewed potential of IKI by comparing its antimicrobial efficacy at both conventional (2%) and elevated (5%) concentrations as a final irrigant in endodontic retreatment cases associated with chronic apical periodontitis (AP) [154]. The findings revealed that the 5% IKI (iodine potassium iodide) solution demonstrated a more pronounced microbial reduction. This effect is primarily attributed to its mechanism of action, which involves the oxidation of essential cellular components, leading to rapid bacterial cell death. IKI is particularly effective against a broad spectrum of microorganisms, including those within biofilms, making it a potentially valuable final irrigating agent, especially in complex clinical cases [130, 154].

Despite these advantages, IKI has not emerged as a widely adopted irrigant in contemporary endodontic practice due to several limitations. Its antimicrobial action is short-lived, as it becomes inactivated in the presence of organic matter. Concerns about potential tissue toxicity, unpleasant taste, staining of dentin, and allergic reactions further limit its routine use. Moreover, the limited number of high-quality clinical studies supporting its long-term efficacy justifies the need for further research to optimize its application and safety in endodontic therapy [154].

In the present review, Ca(OH)₂ was utilized as a sole ICM in approximately 31.48% of the included studies, where it consistently demonstrated effectiveness in significantly reducing the microbial load within the root canal system [181]. Its high pH environment disrupts bacterial cell membranes and denatures proteins, leading to the inactivation of a wide range of endodontic pathogens [172, 181]. However, despite its widespread use and proven efficacy, Ca(OH)₂ has notable limitations. It shows limited effectiveness against resistant species such as Enterococcus faecalis, which can survive in its high pH. Additionally, it has a limited ability to disrupt biofilms or penetrate deeply into dentinal tubules, reducing its antimicrobial action. Prolonged application may potentially compromise tooth integrity [121].

Ca(OH)₂ has been combined with other agents, such as CHX or silver nanoparticles, to enhance its antimicrobial effect [121]. Ca(OH)₂ + 2%CHX combination was used in 10.51% of the studies, which showed synergistic antibacterial activity, particularly in retreatment and persistent periapical lesions [172]. However, care must be taken when combining medicaments, as chemical interactions may alter their properties or produce cytotoxic byproducts [121].

CHX demonstrated a significant antimicrobial effect when used independently as an ICM, although it was used in only a few of the studies (7.41%). These findings supported the role of CHX as a valuable component in endodontic disinfection protocols, particularly in cases where extended antibacterial action is required [33, 37]. Despite the emergence of various novel materials and formulations, Ca(OH)₂ continues to be regarded as the gold standard in endodontic disinfection. Its long-standing clinical use is supported by substantial evidence demonstrating its disinfection efficacy, biocompatibility, and ability to promote periapical healing [140, 155, 181].

### Adjunctive strategies

#### Passive ultrasonic irrigation (PUI)

A widely used modality of ultrasonic-activated irrigation (UAI) is Passive Ultrasonic Irrigation (PUI), which involves the non-cutting activation of an ultrasonically oscillating file or tip within a previously shaped and filled canal space. PUI was the most used activation method in the included studies when it was documented (12.35%) and was found to significantly enhance disinfection outcomes and the disinfection efficacy of the irrigant [27, 95].

However, only two studies reported contrasting results. One study compared PUI with Conventional Needle Irrigation (CNI) and found no significant differences in microbial reduction, suggesting that PUI did not offer a measurable improvement over CNI in that specific context [38]. The second study compared PUI with the Self-Adjusting File (SAF) system and found that PUI yielded inferior disinfection outcomes. This discrepancy can be explained by the continuous irrigant flow with the SAF system, which highlights its effectiveness. These findings suggest the need for further comparative research to clarify the clinical significance and limitations of PUI under different treatment conditions. The findings from Rodrigues et al. [58] highlight the potential limitations of PUI in certain clinical scenarios, particularly in retreatment cases where anatomical complexities and persistent infections are more prevalent.

#### Photodynamic therapy (PDT)

It is a light-based disinfection strategy that uses a photosensitizing agent activated by a specific wavelength of light (commonly red or blue light) [155]. PDT has presented effective antimicrobial action against planktonic bacteria and biofilms, including highly resistant species like *Enterococcus faecalis*. Moreover, its non-thermal mechanism of action reduces the tissue damage and cytotoxicity [155]. PDT was used in 11.11% of the included studies as an adjunct to conventional irrigation protocols. The enhanced disinfection is mostly caused by the reactive oxygen species production that leads to oxidative damage of bacterial cells [125, 155]. However, the efficacy of PDT can be multifactorial due to the heterogeneity of treatment protocols and parameters. Furthermore, there are few randomized controlled clinical trials available that compare the adjunctive use of PDT and its effects on overall endodontic treatment outcome, thereby questioning its true applicability in everyday clinical practice [182].

#### Laser activated irrigation (LAI)

In endodontics, LAI has been considered as a valuable adjunct that provides enhanced disinfection efficacy, particularly in inaccessible areas to standard chemo mechanical preparation methods. Various laser systems, diode laser, Erbium: Yttrium-Aluminum-Garnet (Er: YAG) and Neodymium: Yttrium-Aluminum-Garnet (Nd: YAG) lasers have been extensively studied for their effectiveness in endodontic disinfection [183]. Moreover, newer Er: YAG-based technologies, such as Photon-Induced Photoacoustic Streaming (PIPS) and Shock Wave Enhanced Emission Photoacoustic Streaming (SWEEPS), have shown promising results in enhancing irrigation efficacy through photoacoustic streaming [184].

Most of the included studies evaluating the potency of LAI found that diode laser activation significantly enhanced antimicrobial action in comparison to the use of NaOCl alone [108]. These findings suggest that diode laser may increase the penetration depth and activation of irrigating solutions, hence offering more effective elimination of resistant microorganisms [108, 167, 184]. It is noteworthy that most of the studies evaluating the LAI concluded that diode laser activation demonstrated inferior disinfection effects when compared to PDT. This may be explained by the different mechanisms of action between the two modalities. Diode lasers depend on thermal effects while PDT depends on reactive oxygen production from activation of a photosensitizer. These differences could explain the observed variation in their efficacy. Nevertheless, while several studies support the potential of diode laser activation as a valuable adjunct in root canal irrigation, the overall evidence remains inconclusive. Variability in study design, laser parameters, and assessment methods has led to inconsistent findings, and some studies have reported no significant advantage over conventional irrigation techniques. Therefore, further high-quality, standardized clinical trials are necessary to clarify its efficacy and determine its long-term clinical benefits [20, 106, 162].

#### Biomimetics strategies

By simulating natural biological processes, biomimetic disinfection techniques in endodontics seek to effectively control microbes while maintaining the integrity of periapical tissues. These methods put an emphasis on biocompatibility and decreased cytotoxicity of disinfection agents [185]. Additionally, using naturally-derived materials offer ecofriendly and sustainable alternatives to conventional chemically-based agents.

According to one study, *Calendula officinalis (C. officinalis)* extract as irrigation could inhibit the growth of the frequent bacteria found in root canals, such as *Streptococcus mutans* and *Enterococcus faecalis. C. officinalis* is rich in flavonoids, triterpenoids, saponins, and essential oils, which have antimicrobial, anti-inflammatory, and antioxidant properties. These ingredients could disrupt microbial enzyme systems and damage bacterial cell walls. A significant reduction was observed with both irrigants, C. officinalis and NaOCl. However, no significant difference was found between the two in terms of antimicrobial efficacy [100].

Another study compared the antimicrobial efficacy of Garlic-Lemon extract with NaOCl and found that it was as effective as NaOCl, with a higher mean bacterial reduction percentage. The main active component of garlic is allicin, which destroys the cell wall and cell membrane of root canal bacteria and thus has been suggested as an irrigant alternative to NaOCl [186].

Four of the 148 included studies utilized the concept of natural ICM as Bromelain Paste [96], garlic extract [120], glycyrrhizin [117] and Chitosan (CS) [104]. Garlic is one of the most examined medicinal plants, which may be due to its natural phytochemicals that give it antibacterial, antifungal, and antiviral properties. A wide spectrum inhibitory effect on the growth of various types of Gram-positive and Gram-negative bacteria may be attributed to its extract [187].

On comparing the garlic extract or glycyrrhizin with the Ca (OH)2, they had comparable effects in microbial reduction; however, combining the garlic extract with Ca (OH)2 showed a reduced antibacterial activity for both of them [117, 120].

In a study that assessed the antibacterial effect of bromelain Paste and TAP, surprisingly, bromelain paste performed better regarding bacterial reduction after a period of 7 days of application [29]. Regarding the antimicrobial effect of chitosan, its positively charged amino groups interact with the negatively charged bacterial cell walls, causing disruption of the cell membrane’s integrity. It was found that its effect is enhanced when combined with 2% CHX, even more than the effect of CHX alone [104].

### Endotoxin reduction

Endotoxin is one of the virulent factors contributing to the development of periapical inflammation even in the absence of viable bacteria. Lipopolysaccharides (LPS) are the principal factors of Gram-negative bacteria in primary infections. They correlate directly with periapical inflammation and clinical symptoms such as pain, swelling, and sinus tracts. While Lipoteichoic Acid (LTA) is the relevant virulence factor of Gram-positive bacteria in persistent disease and chronic apical periodontitis [31]. In the current review, 23 studies measured endotoxin reduction and consistently showed that endotoxin elimination is limited and less successful than microbial reduction. There were two main biological reasons accounting for this discrepancy. First, LPS is a structurally stable molecule that retains its biological activity even after bacterial death; therefore, bacterial killing does not correlate to the canal detoxification and LPS resists the oxidative effect of NaOCl. Second, LPS has a high affinity for dentin, where it binds to calcium ions of root canal walls, thereby making it resistant to removal by irrigation only. These mechanisms highlight the importance of time-dependent medication is crucial [173, 188, 189]. Evidence suggests that the application of a chelating agent, such as EDTA, can disrupt this interaction and facilitate the removal of LPS from the root canal system [190]. Furthermore, the subsequent placement of ICM, like Ca(OH)₂, has been shown to enhance the inactivation of residual endotoxins, contributing to improved disinfection outcomes and promoting periapical healing [172]. PDT has emerged as one of the methods for efficient reduction of endotoxin, particularly in secondary AP. On the other hand, one study included in this review questioned PDT’s efficacy on endotoxin reduction. The limited impact of PDT on endotoxins may be explained by the inherent structural complexity and chemical resilience of LPS, which are not easily disrupted by oxidative stress alone. Furthermore, the penetration depth of both the photosensitizing agent and light energy used in PDT may not be sufficient to reach and neutralize endotoxins embedded deep within the dentinal tubules or biofilm matrix [53]. While PDT is a promising adjunctive method for microbial control, its role in endotoxin neutralization appears limited [182]. This highlights the need for combining PDT with other therapeutic strategies, such as Ca(OH)₂, which has shown greater efficacy in endotoxin reduction [183].

### Factors Influencing Microbial Reduction Outcomes (From Data Synthesis)

Notably, several variables that influence the rate and extent of microbial reduction, such as root canal anatomy, the composition and load of the microbial biofilm, host immune response, and the specific instrumentation and irrigation techniques used, also have a direct impact on both the prognosis and long-term survival of the endodontically treated tooth [191].

#### Tooth-related factors

The majority of studies included in this review primarily involved single-rooted teeth, which are characterized by simpler canal anatomies. Previous studies [191] have indicated that the primary factor influencing treatment outcomes between single- and multi-rooted teeth is the complexity of the canal system. However, in studies involving multi-rooted teeth [12, 15, 132], microbiological sampling was predominantly conducted in the largest canal, which may not accurately reflect the potential differences in microbial reduction between canals of varying complexity, thus potentially limiting the generalizability of these findings.

The scope of this review includes both primary and secondary endodontic treatments. Primary root canal treatment (RCT) addresses initial infections and is generally characterized by higher microbial diversity. In contrast, secondary RCT targets cases of prior treatment failure, which are often associated with persistent and more resistant biofilms within inadequately disinfected canal systems [1, 4]. Notably, most studies addressing secondary treatments did not state whether the infection was secondary or persistent. This may be due to overlapping clinical presentations, which frequently make the distinction challenging [170, 192].

As detailed in our results, 82.43% of included studies focused on primary RCT, 15.54% on secondary RCT, and 2.03% on both. This distribution reflects the predominance of primary cases in the existing literature; the inclusion of both treatment modalities was intentional, allowing a comprehensive evaluation of disinfection efficacy across different pulp–periapical disease conditions, particularly PN with AP.

Microbial communities within the root canal are uniquely shaped by the specific pulp and periapical conditions [5, 35]. However, some studies [12, 35] did not observe a significant reduction in microbial load, which may be due to the distinctive microbial composition associated with the underlying tooth diagnosis.

#### Technique-related factors

Apical preparation size influences the effectiveness of bacterial reduction and the overall outcome of root canal treatment [193]. The new era in endodontics has adopted the idea of minimally invasive approaches, which aim to preserve the natural anatomy of the root canal. This approach is made feasible by advances in antimicrobial techniques in the field of endodontics. For instance, Zhang et al. [194] demonstrated that even with a smaller preparation size, such as ISO 15, combined with the GentleWave system, substantial microbial reduction can be achieved. In this review of clinical studies, the most frequently utilized apical preparation size was ISO 40. However, some studies have adopted the principle of minimal preparation, with the smallest file employed being ISO 25 [95, 131, 144], which also yielded successful microbial reduction.

Most of the studies reviewed employed a multiple-visit protocol. Existing literature suggests that the number of visits may influence the reduction of both microbial populations and endotoxins [67]. When employing a multiple-visit strategy, the use of intracanal medicaments has been suggested to prevent bacterial recolonization. However, it is advisable to repeat the final irrigation procedure during the second visit, as samples obtained after the application of intracanal medication have been shown to exhibit increased microbial load again addressing the question of the need for multiple visits [77].

#### Assessment methods (Sampling and detection methods)

Microbial sampling and detection are essential steps in root canal treatment and may serve as reliable indicators of the healing process [195]. The majority of the studies included in this review employed paper points for microbial sampling, with the intracanal space being the most common source for sample collection. However, some studies also included samples obtained from periapical tissues and purulent exudates, all of which demonstrated significant bacterial reduction [31, 33, 69]. However, the lack of standardized microbial sampling among the included studies introduced considerable heterogeneity with different levels of sensitivity.

In terms of microbial detection, culture-based analysis was the most prevalent method, employed in 96 (64.86%) studies. While culture techniques remain the standard, they have inherent limitations, such as the potential for false-negative and false-positive results due to the unculturable bacteria present in the apical ramification or covered by remaining root canal filling material [78, 195]. The second most used method was molecular analysis in 86 (58.10%) studies and is recognized for its enhanced sensitivity and broader detection capabilities. Additionally, several studies have employed biochemical assessment methods (23 (15.54%) studies), such as quantification of endotoxin levels, to enhance the evaluation of microbial load. Interestingly, 2 out of 148 studies included in this review utilized next generation sequencing (NGS) to examine the endodontic bacteriome [114, 132]. NGS allows a deeper analysis of complex microbial systems with high accuracy and reduced errors, which may offer more valuable insight to design more targeted antimicrobial therapies in endodontics [132]. Consequently, the resulting microbial reduction should be interpreted cautiously.

### Limitations

The current review revealed several heterogeneities/inconsistencies in methodological approaches, microbial sampling techniques, and detection methods, which complicate the interpretation of results and the correlation between microbial reduction and treatment outcomes. These heterogeneities also precluded the quantitative analysis of microbial reduction outcome. Therefore, the findings of this review may represent a range of potential clinical results rather than promote a definitive single protocol.

1. Methodological Heterogeneity:
  The distribution of study designs highlights a temporal shift in research methodology. While 33.8% of the studies were RCTs, the majority (54.1%) lacked specific design categorization, a limitation largely attributable to older publications where structured abstract and method requirements were less rigorous than current standards. This variation in study designs among the included studies leads to differences and inconsistencies in outcome measures, which avert the formulation of clinical guidelines. As previously mentioned, differences in patient demographics, age, tooth types, treatment protocols including variation in canal shaping, irrigation solution, medicaments, and the choice between single-visit versus multiple-visit root canal treatments, can result in varying outcomes among studies, thus hindering the ability to synthesize evidence. Another limitation of the present review is that it focuses on systemically healthy patients with an assumed favorable healing response. However, the study by Ghoneim et al. [123] included patients with controlled diabetes and found that diabetic status did not significantly affect bacterial reduction outcomes. Additionally, the research by Thais M. Duque et al. [46] involved patients with primary periodontal disease, yet successful endotoxin reduction was still achieved, suggesting that the presence of certain controlled systemic or periodontal conditions may not necessarily impede the effectiveness of microbial reduction during root canal therapy.

2. Heterogeneity in outcome measurement; Microbial Sampling & Detection.
  The current review emphasized the lack of standardized outcome measurements. Regarding sampling technique, the use of sterile paper points is the most commonly used sampling technique. Nevertheless, paper points mainly gather planktonic bacteria and inadequately collect biofilms attached to the walls of the canal. It has been proposed that using paper points in conjunction with K-files could enhance the effectiveness of sampling [196].
  Regarding the detection methods, culture methods often fail to identify the presence of all microbial species, particularly those that are difficult to grow or cannot be cultured. The sensitivity of microbial reduction was enhanced by the advanced molecular techniques. However, qPCR can result in potential overestimation of the results as it can detect DNA from non-viable bacteria. NGS offers detailed microbial profiles but necessitates complicated data analysis and may reveal organisms of unclear clinical significance [196]. It is important that multiple detection techniques be used to allow a more comprehensive overview of existing pathogens in different endodontic infections.

3. Lack of Correlation Between Microbial Reduction and Clinical Outcomes.
  The majority of the included studies showed significant microbial reduction regardless of the disinfection therapy used. However, they didn’t correlate microbial reduction with the healing of AP. As a result, the most effective disinfection protocol that guarantee healing outcome aligns with microbial load reduction could not be reached. Assessment of treatment outcome should include evaluation of microbial reduction in conjunction with other clinical factors, like endotoxin level. Several studies have reported that a significant reduction of endotoxin level post-chemo-mechanical preparation could provide better outcomes even in the presence of viable bacteria [31, 43, 197, 198]. Consequently, Endotoxin could be correlated with healing outcome. Additionally, endotoxin level measurements, particularly LPS provide a more clinically significant measure of infection severity and healing capability compared to microbial load alone [197, 198].
  It is crucial to stress that one of the goals of this review was to compile and contrast the current data about the supplementary use of various disinfection modalities in endodontic irrigation rather than to identify a particular “superior” activation method. A straight ranking of PUI, PDT, and LAI is currently impractical due to the significant variation among studies in terms of methodology, clinical protocols, outcome measures, and assessment tools. As a result, this research highlights that each method has possible advantages and disadvantages depending on the clinical situation rather than concluding that one approach is superior to the others.

## Future Implications

Given that microbial reduction during RCT is a complex, multifactorial process, the complex root canal anatomy and the variety of endodontic infections, it is essential to develop a standardized treatment protocol applicable to all cases or an individualized approach tailored to the specific characteristics of each patient and tooth [191]. The lack of well-designed, high-quality randomized controlled clinical trials assessing adjunctive irrigation activation techniques can be considered as significant knowledge gap. The majority of the available evidence is derived from short-term clinical studies or in vitro research with heterogenous protocols, small sample sizes, and inconsistent outcome measures. Therefore, the current evidence is insufficient to draw firm conclusions about the actual clinical efficacy of PUI, PDT, and LAI. Future research should prioritize standardized, sufficiently powered RCTs with clear activation parameters and clinically relevant endpoints, to truly reflect the true value of these adjunctive techniques in routine endodontic practice. While standardized protocols offer ease and efficiency, they may not adequately address the biological and anatomical variations encountered in clinical practice. In contrast, personalized treatment strategies allow for a more meticulous approach, potentially leading to improved microbial reduction and better clinical outcomes. Several studies reported that endotoxin levels determine the adequacy of disinfection even if bacterial DNA is still detected [32, 197–199]. Additionally, their presence is correlated with symptoms, reflects inflammation potential, and could predict clinical outcomes [32, 197, 199]. Consequently, monitoring endotoxin level could allow the potential development of targeted personalized antimicrobial treatment of endodontic infection. Also, the incorporation of functional biomarker determination, like endotoxin, into endodontic practice could be the basis of the development of chair-side predictor tests. However, long term studies are needed to validate the correlation of this test with treatment outcomes.

## Conclusion

The current body of evidence highlights that no single disinfection strategy ensures complete microbial eradication and there is a lack of a consistent correlation between microbial reduction and periapical healing. In conclusion, a combination of advanced irrigation techniques, appropriate intracanal medication, and adjunctive disinfection activation techniques may provide the most effective approach for both microbial and endotoxin reduction. These findings remain exploratory rather than prescriptive, as the high degree of heterogeneity among the included studies prevents the prescription of a single superior protocol. Future research should focus on personalized treatment protocols based on patient and tooth specific considerations to optimize clinical outcomes.

## Supplementary Information

Supplementary Material 1

Supplementary Material 2

## Data Availability

The datasets used and/or analyzed during the current study are available from the corresponding author on reasonable request.

## Abbreviations

AP: Apical periodontitis
NaOCl: Sodium hypochlorite
CHX: Chlorhexidine
EDTA: Ehylenediaminetetraacetic acid
Ca(OH)₂: Calcium hydroxide
PN: Pulp necrosis
C. officinalis: Calendula officinalis
PUI: Passive ultrasonic irrigation
FMX: Ferumoxytol
H2O2: Hydrogen peroxide
HEDP: Etidronic acid (1-hydroxyethane 1,1-diphosphonic acid
PBS: Phosphate-buffered saline solution
SDS: Sodium dodecyl sulfate
PDT: Photodynamic therapy
LAI: Laser activated irrigation
IP: Irreversible pulpits
PT: Previously treated
AAA: Acute apical abscess
IKI: Iodine-potassium iodide
ICG: Indocyanine green
ICM: Intracanal medication
LPS: Lipopolysaccharides
CNI: Conventional Needle Irrigation
SAF: Self-Adjusting File
PDT: Photodynamic therapy
Er: YAG: Erbium: Yttrium-Aluminum-Garnet
EA: Endoactivator
XPF: XP-endo finisher
PP irrigation: Positive pressure irrigation
EC: Easy Clean tip
CHG: Ca (OH)2 in glycerin
CHPG: Ca (OH)2 in CPMC and glycerin
nano-Ag: Nano-silver
nano-CH: Nano-calcium hydroxide
CPMC: camphorated paramonochlorophenol
SE: Selenium
CS: Chitosan
TAS: Triple antibiotic solution
GP: Gutta percha
AA: Apical abscess
Nd: YAG: Neodymium: Yttrium-Aluminum-Garnet
TAP: Triple antibiotic paste
LTA: Lipoteichoic Acid
RCT: Root canal treatment
CFU: Colony forming units
