# Supplementary material for: The conundrum of current endodontic disinfection strategies in microbial load reduction: a scoping review
Source: BMC Oral Health. 2026 Mar 18;26:612. doi: 10.1186/s12903-026-07952-0 (PMC13063732; doi:10.1186/s12903-026-07952-0)
Supplement: Supplementary file 2 — Supplementary Material 2 [file 12903_2026_7952_MOESM2_ESM.docx]

| **Supplementary table 1: general characteristics of included studies** | | | | | | |
| --- | --- | --- | --- | --- | --- | --- |
| Author |  | Year | Study design | Sample size | Groups/ number | General findings |
|  |  |  |  |  |  |  |
| Sarkees et al. | (1) | 2025 | RCT | 30 patients | 2 groups (Group 1(n=15) : irrigated with 2% IKI , - Group 2( n=15): with 5% IK ) | 5% IKI solution as the final irrigating agent in endodontic retreatment cases with chronic AP significantly reduces bacterial counts compared to a 2% IKI solution. The 5% IKI solution exhibited a superior antibacterial effect. |
| Nogales et al, | (2) | 2025 | CT | 35 single-rooted teeth | Single arm | Ultrasonic irrigation improved the disinfection of root canals after CMP by reducing bacterial levels. The rRNA levels were signigicantly higher than DNA levels in DNA-positive samples. The rRNA to DNA ratio indicated bacterial activity in most samples, even after ultrasonic irrigation. |
| Kavalipurapu Venkata Teja | (3) | 2024 | Not mentioned | 45 patients | 3 groups (Group I (n=15): Disinfection was carried out completely using 5.25% NaOCl with an automated irrigation device at a constant rate of 6 ml/min. Respective irrigants were filled in separate syringe barrels and coupled to the automated device during the course of irrigation with a 30G closed‑ended lateral vented needles for 15 min. . Group II(n=15): During instrumentation, 5.25% sodium hypochlorite NaOCl was used with 30G open‑ended flat irrigation needles for 15 min,Group III(n-15): 5.25% NaOCl was used 1 mm short of the apex with 5 ml of a disposable plastic syringe with a 30G closed‑ended lateral vented tip for 15 min, | The automated irrigation technique was effective in reducing both aerobic and anaerobic CFU. |
| Shroff et al | (4) | 2024 | CT | 28 patients | 2 groups ( Group D(n=14): Double antibiotic paste (DAP) group and group S(n=14): Simvastatin.) | DAP and simvastatin were effective in the reduction of the bacterial count in single‑rooted teeth diagnosed with AP . However, DAP demonstrated a better performance in the reduction of bacterial counts. |
| Sesar et al | (5) | 2024 | RCT | 40 patients | 2 groups (Group 1: Reciprocating WOG instrumentation (n=20) & Group 2 Rotary PTN instrumentation (n=20)) | CMP with both instrumentation techniques and the final irrigation protocol significantly reduced the number of CFUs. There were no statistically significant differences in antibacterial efficacy between WOG and PTN groups . |
| Konadu et al | (6) | 2024 | Not mentioned | 60 teeth from 44 patients | 4 groups (based on NaOCl concentration (0.5%, 1.0%, 2.6%, 5.2%) with n=15 per group) | All concentrations of NaOCl showed a significant reduction in microbial counts post-treatment |
| Kesim et al | (7) | 2024 | Longitudinal Experimental | 3 patients | Single arm | Chemomechanical preparation and intracanal calcium hydroxide medication significantly reduced microbial diversity and highlighted resistant bacterial taxa |
| Hepsenoglu et al. | (8) | 2024 | RCT | 30 patients | 2 groups (SWEEPS mode of the Er: YAG laser(SL)(n=15) and passive ultrasonic irrigation(PUI)(n=15)) | Supplementary steps consisting of SWEEPS activation and PUI promoted further decrease in the bacterial bioburden to levels below those achieved by the chemomechanical procedures alone. SWEEPS activation and PUI were equivalent in terms of reduction in root canal bacteria including Streptococcus species and reduction of inflammation detected by IL-1β |
| Fahim et al. | (9) | 2024 | RCT | 30 patients | 3 groups (A:Conventional group: 2.5% Sodium Hypochlorite (NaOCl) and 17% EDTA solution NaOCl/EDTA,B: Dual laser group: 2780 nm Erbium, chromium: yttrium scandium-gallium-garnet (Er,Cr:YSGG) laser and 940 nm diode laser Er,CrYSGG/Diode, and C:Combined group: 17% EDTA and 940 nm diode laser EDTA/Diode.n=10 per groups ) | both dual laser Er,CrYSGG/Diode and combined laser EDTA/Diode groups provide superior bactericidal effect compared to the conventional NaOCl/EDTA group. |
| Eltantawi et al | (10) | 2024 | RCT | 36 patients | 3 groups (Group I (n = 12): Ca(OH)2 powder, Group II (n = 12): Ledermix paste and Group III (n = 12): Glycyrrhizin gel) | All tested ICM documented a significant reduction in the CFUs. There was no significant difference between Glycyrrhizin and Ledermix.Glycyrrhizin and Ledermix showed comparable antimicrobial effects that were better than Ca(OH)2. Glycyrrhizin may be a promising ICM. |
| Barazy et al | (11) | 2024 | RCT | 45 patients | 3 groups( n=15 canals): Group A:Ca(OH)2 . Group B: Passive Ultrasonic Activation (PUI). Group C: Antimicrobial Photodynamic Therapy (aPDT) | PDT showed no statistically significant difference when compared to PUI but showed higher and more promising results when compared to Ca(OH)2 .PDT can be considered an adjunct method for root canal disinfection with the same effectiveness as PUI. |
| Babeer et al | (12) | 2024 | CT | 44 patients | 3 groups (FMX/H2O2 (test group), saline (negative control), and NaOCl (positive control),n=16 per group) | Data show robust antibiofilm activity by a single application of FMX with H2O2 achieving results comparable to those seen with NaOCl without adverse effects and 99.9% reduction in bacterial counts, consistent with the data from the ex vivo biofilm model. FMX promotes growth of stem cells of the apical papilla (SCAPs) |
| Alquria et al. | (13) | 2024 | Cross-Sectional Interventional | 18 patients ( final analyzed number) | Single arm | The present study showed a quantitative and qualitative impact of CMP using 2.5% NaOCl in the bacteriome present in teeth with PEI with AP. The qPCR analysis showed a significant reduction in bacteria after treatment. Our findings revealed a distinct community composition and increased alpha diversity after CMP using 2.5% NaOCl, despite a dramatic decrease in bacterial abundance and intra-individual similarity in pre-and post-treatment bacteriome. Differential enrichment of Stenotrophomonas_unclassified, Enterococcus_unclassified, and Actinomyces_unclassified at s2 suggests lower effectiveness of CMP using 2.5% NaOCl against these specific taxa. |
| Abdel-Hamid et al.. | (14) | 2024 | RCT | 34 patients | 2 groups (intervention group; Dual Rinse + 2.5% NaOCl, control group; 2.5% NaOCl irrigation). dual rinse (9% Dual Rinse HEDP) composed of Etidronic acid, also known as HEDP or 1-hydroxyethane 1,1-diphosphonic acid) | no increase in the incidence of post-instrumentation pain by the addition of Dual Rinse to 2.5% NaOCl. The antimicrobial efficacy of the NaOCl was not affected, with no increase in periapical MMP-9 and no obvious periapical inflammation in the form of postoperative pain. Both irrigating solutions successfully reduced bacterial counts from primary infected root canals, without any significant difference between them. The mean % reduction for the total bacterial count was higher in Dual Rinse + NaOCl group than in the NaOCl group with no statistically significant difference between the two groups (p=0.759) |
| Wenzler et al. | (15) | 2023 | Not mentioned | 26 patients | Single arm | CMP with a 3% NaOCl rinsing solution significantly reduced the bacterial count in the root canal, as did additional rinsing with NaOCl after the root canal treatment. Temporary administration of Ca(OH)2 for 7 d did not prevent the bacterial count from returning to the level immediately after chemomechanical treatment. The study shows that bacterial recolonization should be expected when Ca(OH)2 paste is used for temporary medicinal treatment of the root canal. However, the recolonization can be significantly reduced again by additional rinsing with NaOCl |
| Rodríguez et al | (16) | 2023 | Not mentioned | 27 patients | Single arm | There was a significant bacterial decrease between the pre CMP, post CMP, and post ICM samples. A correlation was found between the initial size of the lesion and the number of bacteria. |
| Rôças et al | (17) | 2023 | Not mentioned | 90 root canals | 3 groups ( according to the type of ICM used: (1) Ca(OH)2 in glycerin (CHG); (2) Ca(OH)2 in CPMC and glycerin (CHPG); and (3) Ca(OH)2 in 2% CHX (CHCHX).n=10 per group ) | Comparison between the 3 Ca(OH)2 pastes showed no significant differences in antibacterial effectiveness in the main root canal. However, only the CHPG paste showed a significant reduction in bacterial counts. |
| Rajamanickam et al | (18) | 2023 | RCT | 32 patients | 2 groups (Group A used CNI:(n=16), while Group B used LAI:(n=16)) | LAI and CNI were effective in bacterial reduction. There was greater bacterial reduction with LAI (25.92%) than with the CNI (10.82%) in single-rooted teeth with pulpal necrosis with no significant difference. Er,Cr:YSGG LAI has shown to be effective in root canal disinfection. |
| Sadhana Rai | (19) | 2023 | RCT | 45 patients | 3 groups (group1(n=5)= NaOCl, group2(n=15)= diode laser and group3(n=15)= photodynamic therapy) | According to the results of this in vivo study, a single disinfection strategy could only reduce 60% of microbial load in comparison with preoperative samples. |
| Laís Lima Pelozo1 | (20) | 2023 | RCT | 30 patients | 2 groups (Group 1 (n=15): received the root canal retreatment (RCR) combined with 980-nm diode laser irradiation (LI). Group 2 (n=15): received the RCR with placebo irradiation (PI).) | The bacterial counts were lower after laser irradiation when compared with placebo irradiation, showing an antimicrobial effect before the biomechanical prepa ration. The laser facilitated the periapical repair from 3- to 12-month follow-ups and had 45% more healed cases than the placebo group at 1 year. |
| Revathi Palanisamy | (21) | 2023 | RCT | 80 Patients | 2 groups (Group A receiving side-vented needle irrigation, and Group B receiving passive ultrasonic irrigation using Irrisafe tips.) | Passive ultrasonic irrigation with Irrisafe tips may be more effective than side‑vented needle irrigation in reducing postoperative pain and intracanal bacterial load in patients undergoing endodontic treatment. |
| Nikhade et al | (22) | 2023 | Not mentioned | 30 patients | 2 groups (Group 1: Triple Antibiotic Paste (n=15) and Group 2: Bromelain Paste (n=15)) | No significant difference in E. faecalis reduction between groups before and after instrumentation. Significant reduction in E. faecalis for Bromelain group after 7 days |
| Leonardo et al | (23) | 2023 | Not mentioned | 60 patients | 3 groups ((n = 20 each), based on the final instrumentation size (25/04, 30/04, 35/04). In half of the patients (n=10) in each group, 2.5% sodium hypochlorite was used as an irrigating solution, and in the other half (n=10) saline solution was used. 1A: 25/04 + 0.9% saline, 1B: 25/04 + 2.5% NaOCl, 2A: 35/04 + 0.9% saline, 2B: 35/04 + 2.5% NaOCl, 3A: 30/04 + 0.9% saline, 3B: 35/04 + 2.5% NaOCl) | There was a significant reduction in cCFU from S1 to S2 in all treatment protocols. LA therapy with ICG further reduced the microbial counts significantly |
| Khandelwal et al. | (24) | 2023 | RCT | 42 patients | 2 groups (Group A(n=21):5.25% sodium hypochlorite gel. Group B(n=21): 5.25% sodium hypochlorite aqueous solution) | Both groups showed a significant reduction in CFUs post-treatment, indicating effective disinfection. The mean CFU count difference between the 5.25% NaOCl gel and aqueoussolution groups did not differ in a manner that was statistically significant |
| Karataş et al | (25) | 2023 | RCT | 45 patients | 3 groups (according to the ICM; Ca(OH)2, TAP and DAP.) | 15 of days ICM with TAP causes tetracycline resistance. In contrast, DAP does not cause tetracycline resistance and it has similar antibacterial effectiveness to TAP. The DAP would be the choice of medicament rather than TAP in clinical practice. |
| Hepsenoglu et al | (26) | 2023 | RCT | 20 patients | 2 groups ( according to the irrigation activation method used: XPF and EA group. (n=10 per group)) | There was no statistical difference between the XPF group and the EA group in terms of E. faecalis copy number. Although both the XPF and the EA optimised the antibacterial efficiency of chemomechanical preparation in previously root canal-treated teeth with AP, a lower total bacterial copy number was achieved with the EA application than the XPF application. |
| Seyda Ersahan | (27) | 2022 | RCT | 16 patients | 2 groups (according to the intracanal medicament used. group A: calcium hydroxide and group B : CHX) | CMP with NaOCl lead to a considrable reduction in bacterial load. For the CHX group, its placement in S3 did not provide any superiority in terms of total bacterial reduction during CMP. However, complementing conventional en dodontic treatment with CH(OH)2 appears to be very effective in reducing the complete microbiota |
| Rakasevic Dragana | (28) | 2023 | Not mentioned | 44 patients | 3 groups (The control group was subjected solely to the conventional endodontic treatment procedure. Group 2 : photodynamic therapy and group 3: high-power diode laser) | Concerning conventional treatment in all three experimental groups, it was successful in reducing number of isolates, but complete eradication was not observed for any of isolated species. within DL group, diode laser therapy also increased efficacy of overall endodontic treatment.It completely removed isolates of 6 species/genera, while all the others were reduced significantly more (up to 76% inhibition). The radiographic analysis performed six months after endodontic treatment revealed that PDT and DL induced significantly higher decrease of the diameter of periapical lesion, comparing to control. |
| Alves-Silva et al, | (29) | 2023 | CT | 24 patients | 2 Groups (conventional group, whose patients received conventional endodontic treatment with CMP alone (n = 12) and PDT group, whose patients received PDT after CMP (n = 12)) | PDT as an adjunct to CMP proved to be effective in improving the root canal disinfection and reducing the levels of LPS and LTA in teeth with primary endodontic infection. |
| Toia et al. | (30) | 2022 | RCT | 40 teeth | 2 groups ( Group 1: Rett in 1 visit I(n=20), - Group 2: Rett in 2 visit (n=20)) | Even with the remaining content of LPS and LTA bacteria, it was possible to observe a significant reduction in the volume of periapical lesions in both groups after 18 months of treatment with between teeth treated in 1 visit and those treated in 2 visits using Ca(OH)2 for 14 days. |
| Tandon et al. | (31) | 2022 | Not mentioned | 28 patients | 2 groups ( Group 1 (n=14)- Final irrigant as SmearOFF (Vista Dental Products, USA)+CHX (2% Gel, Cerkamed Medical Co. Poland) as intracanal medicament. b. Group 2 (n=14)- Final irrigant as 5.25% NaOCl (Coltene, Switzerland) followed by Calcium Hydroxide (Apexcal, IvoclarVivadent, Schaan, Liechtenstein) as intracanal medicament.) | significant reduction in bacterial load of Aerobic bacteria, Anaerobic bacteria, after chemomechanical preparation with reciprocating kinematics for GP removal combined along with irrigant activation with PUI device and this protocol may be recommended for retreatment cases. SmearOFF as the final irrigant caused significantly more reduction in bacterial load for E. faecalis and F. nucleatum in comparison to NaOCl. 2 % CHX gel may be advisable in retreatment cases as it was reported to besignificantly effective against E. faecalis. with supplemental instrumentation with XP Endo Finisher |
| Saber et al. | (32) | 2022 | RCT | 66 patients | 2 groups (WaveOne Gold (WO) used in reciprocation medium file (#35.06) or One Shape (OS) used in continuous rotation One Shape file (#37.06) (Micro-Mega), as a single-file.) | no difference in the bacterial reduction or incidence of post-operative pain and flare-ups when reciprocation or continuous rotation motions were used during root canal preparation of single-rooted premolars with asymptomatic apical periodontitis. that PCR analysis demonstrated significantly higher pre-instrumentation baseline bacterial count (p < .05). The comparison between counts before and after instrumentation showed a significant decrease in the percentage of bacterial reduction after using either rotation or reciprocation kinematics (p < .05). However, the difference between the WOG or OS files was statistically non-significant (p > .05). |
| Mishra et al | (33) | 2022 | CT | 45 patients | 3 groups ( Group I(n=15): Diode laser disinfection in continuous mode for 20 seconds. Group II(n=15): Diode laser disinfection in pulse mode for 20 seconds. Group III(n=15): Irrigation with 5.25% NaOCl for 5 minutes.) | All three groups showed significant differences and an overall reduction in the microbial count at post-biomechanical preparation (BMP) as compared to pre-BMP with the highest being in laser in continuous mode (group I) (91.9%), followed by 5.25% sodium hypochlorite (group III) (86.5%) and LASER in pulse mode (group II) (72.0%) the least. |
| Omer et al | (34) | 2022 | RCT | 66 patients | 3 groups (n = 22 each): Group I (A): Ca (OH), Group II (B): Garlic extract (Allium sativum), Group III (C): A combination of Ca (OH)2 powder and garlic extract | Significant reduction in bacterial load after mechanical instrumentation (S1 → S2)Garlic alone showed a significantly higher antibacterial effect against E. faecalis than other groups at 7 and 14 days. |
| Gabrielli et al | (35) | 2022 | Cross-Sectional | 20 patients | 2 groups based on clincal and radiographic disgnodis; G I(n=10) : symptomatic ,those with symptomatic pulpal necrosis with AAA and G II (n=10):asymptomatic .those with asymptomatic pulpal necrosis with Ap | CMP was able to reduce bacterial content and the levels of LPS, but not of LTA in the symptomatic group. Different species were detected in all stages of the endodontic treatment. Fusobacterium nucleatum and E. faecalis were frequently identified in both groups, alone or in combination. High levels of LPS were correlated with spontaneous pain and pain to percussion in the symptomatic group. |
| Fahim et al | (36) | 2022 | RCT | 69 patients | 3 groups ( according to the type of ICM used; Nano-Ag group,nano-CH group, CH group (n = 23) per group ) | The antibacterial effect of the nano-Ag and nano-CH was equivalent to that of CH. Post-operative pain was significantly reduced at the 48- and 72-h intervals after the application of nano-Ag and nano-CH only, with no significant difference between these two ICM. |
| V. Di Taranto | (37) | 2022 | CT | 50 patients | 2 groups( group1(diode laser) and group 2 (photodynamic therapy)) | The tested PDT shows positive and clinically relevant results when used as an adjuvant to traditional mechanical and chemical cleansing. There is a further reduction after a second treatment with PDT. The decrease in CFU post PDT is similar before and after dressing with Ca(OH)2. PDT as an adjuvant to traditional mechanical and chemical cleansing has shown greater efficacy in controlling the contamination than the treatment with diode laser. The first PDT causes a reduction in the microbial count, and the second, after intermediate dressing, is comparable to the first. This is probably due to a lower complexity of the biofilm created after the intermediate dressing with Ca(OH)2 compared to the initial intracanal biofilm. This therapeutic adjuvant is, therefore, more suitable in clinical situations where an intermediate dressing is necessary. |
| Rodrigo Arruda-Vasconcelos | (38) | 2022 | CT | 10 patients | Single arm | Bacteria were present in all samples. A total of 260 strains were detected at the baseline, and after CMP the number of detected strains was significantly reduced to 215 (P <.05). After ICM, 127 strains remained |
| Wenzler et al, | (39) | 2021 | RCT | 57 patients | 3 groups ( Group 1: 5 mL of 3% NaOCl rinsing alone for 1 min. Group 2: 445-nm diode laser irradiation in continuous-wave mode. Group 3: a combination of NaOCl rinsing and laser irradiation.) | At baseline and after CMP there were no statistically significant differences in the numbers of bacteria detectable in the root canals. Statistically significant reductions in the bacterial load were observed in all three groups. The largest reduction was observed in with the combination of NaOCl rinsing and laser irradiation. |
| Nasr et al. | (40) | 2021 | RCT | 60 patients | 4 groups (Group A: CNPs ( CHITOSAN NANO PARTICLES). Group B: CHX, Group C: CHX/ CNPs combination, and Group D: 5.25% NaOCl) | 3% CNPs and its combination with 2%CHX are significantly more effective than both 2% CHX and 5.25% NaOCl against anaerobic bacteriaand are not significantly different from each other. • The addition of 2%CHX did not cause a significant improvement to the antibacterial effectiveness of 3% CNPs. • Both 3% CNPs and its combination with 2% CHX were associated with lower postoperative pain scores than either 5.25% NaOCl or 2%CHX. • Post-operative pain values were highest in the first 24 h and gradually decreased over time until complete subsidence within a week. |
| Moreira et al | (41) | 2021 | Not mentioned | 50 patients | 2 groups (Group 1 (n=30): Endodontic Therapy with Mechanical Chemical Preparation (MPQ) and intracanal medication; Group 2 (n=20): Endodontic therapy with MPQ, intracanal medication, and 2 applications of aPDT.) | aPDT was not superior to the conventional therapy in eliminating microorganisms |
| Dalaei Moghadam et al | (42) | 2021 | RCT | 90 patients | 3 groups ( Depotphoresis Group (n=30), Diode Laser (DL) Group (n=30), and Control Group (n=30)) | A statistically significant reduction in the bacterial count was noted in the diode laser and depotphoresis groups, respectively. Post-treatment, all patients reported only mild pain across all time intervals. The size of the preoperative periapical lesions did not have a significant impact on the outcome of endodontic treatment. |
| KARATAŞ et al | (43) | 2021 | RCT | 45 patients | 3 groups ( according to the final irrigation selected: NaOCl 2ºC, NaOCl 25ºC and NaOCl 45ºC. n=15 per group) | Final irrigation with NaOCl at different temperatures results in similar antibacterial effectiveness. CMP and final irrigation with EDTA + NaOCl were highly effective in significantly reducing the bacterial counts, irrespective of the NaOCl temperature. Final irrigation with cold NaOCl (2˚ C) is better than NaOCl (45˚ C) when comparing postoperative pain levels. |
| Espaladori et al | (44) | 2021 | RCT | 60 patients | 4 groups (according to the ICM used; Se, Ca(OH)2, Ca(OH)2+ Se combination, and a control group that received no medication (n = 15) per group ) | A significant reduction in the microbial load was observed only in the groups that received ICM. The IFN-γ mRNA expression was reduced in the groups treated with the medications (Se, C.H., and C.H. + Se). |
| Bharti et al. | (45) | 2021 | Not mentioned | 54 patients | 3 groups ( G1:5%NaOCl/G2:2%CHX and G3:PDT) | For all groups, there was a statistically significant (P < .001) reduction in the number of CFUs from S1 to S2. The significant reduction of E. faecalis displayed that it was more immune to destruction when instrumentation using 2% CHO was employed as compared to 5% NaOCl. However, after the PDT procedure, maximum reduction was seen |
| Vasconcelos et al. | (46) | 2021 | Cross-sectional study | 10 patients | Single arm | Chemomechanical preparation was effective in significantly decreasing the levels of bacteria, LPS and LTA (P < 0.05). ICM did not provide additional reduction in the levels of bacteria and LPS (P > 0.05). However, a significant reduction in the levels of LTA was observed after ICM (P < 0.05). |
| Yalgi et al. | (47) | 2020 | RCT | 40 patients | 2 groups ( Group 1(n=20): Irrigating with NaOCl and Group 2(n=20): Irrigating using C. officinalis ) | There was a significant reduction in CFUs from S1 to S2 for both irrigation;NaOCl & C. officinalis, however no significant difference was found between them |
| Siddique et al. | (48) | 2020 | RCT | 30 patients | 2 groups ( Group 1(n=15): Irrigating using 3% NaOCl and Group 2 (n=15): Irrigating using 1.8% Garlic-Lemon) | The antimicrobial efficacy of Garlic-Lemon was found to be as effective as NaOCl, with a higher mean bacterial reduction percentage |
| Siddique et al. | (49) | 2020 | RCT | 60 patients | 3 groups (ProTaper Next (n = 20), ProTaper Gold (n = 20) and XP-endo Shaper (n = 20)) | XP-endo Shaper was effective in reducing total bacterial load in root canals when compared with ProTaper Gold pointing to the fact that instrument geometry plays a pivotal role in bacterial reduction. PTN showed better microbial reduction percentage than ProTaper Gold since it maintains a two-point contact with bigger radius on canal walls whereas ProTaper Gold maintains three-point contact with comparatively lesser radius on wall which interferes with removal of bacterial debris from root canal. |
| Orozco et al. | (50) | 2020 | RCT | 20 patients | 2 groups (cni conventional needle irrigation and PUI passive ultra sonic irrigation) | both treatments significantly decreased the number of bacterial species when compared with the initial sample. However, no statistical difference in the total microbial load between PUA and CNI groups was detected. The number of cultivable anaerobic bacteria significantly decreased using PUA; bacterial composition and number of bacterial species found after using CNI or PUA was similar. |
| Neves et al | (51) | 2020 | Not mentioned | 80 patients | 2 groups ( BioRaCe ( multifile system) or Reciproc instruments (single file system).n=40 per group) | Reciproc and BioRaCe groups were significantly effective in reducing intracanal bacterial load, with no statistically significant difference between them. no significant difference between the two systems in periapical healing. both instrumentation techniques showed high success rates |
| Mittal et al | (52) | 2020 | RCT | 48 patients | 3 groups (Group I: Conventional endodontic irrigation , Group II: Photoactivated disinfection (PAD) ,Group III: Conventional Endodontic Irrigation with PAD) (n = 16 per group)) | A statistically significant reduction was seen at S2 among photoactivated disinfection (PAD) (Group I), conventional irrigation (CEI) (Group II), and combination of both (Group III). Mean bacterial reduction in Group III was significantly lower than in the other groups. This difference was statistically significantly better between Group I and Group III; Group II, and Group III. However, no statistically significant difference was seen between Group I and Group II. |
| Louzada et al | (53) | 2020 | Cross-sectional | 10 patients | Single arm | The microbiota of PPs and RCs in teeth with vital pulp and associated periodontal disease is polymicrobial, with the presence of gram-negative, gram-positive, facultative, and strict anaerobes. CMP and Ca(OH)2 ICM allowed the reduction of infectious content in both sites. Levels of LPS and LTA were reduced after the endodontic procedures, although higher concentrations of both had been found in PPs compared with RCs. |
| Karatas et al | (54) | 2020 | RCT | 45 patients | 3 groups (according to the ICM; group 1: Ca(OH)2 , group 2: Ca(OH)2 + Ibuprofen, group 3: Ca(OH)2 + Ciprofloxacinl (n=15) per group) | There were significantly lower bacterial counts in the Ca(OH)2 + Ciprofloxacin group than the pure Ca(OH)2 and Ca(OH)2 + Ibuprofen groups at S3. The percentage reduction from S1 to S3 and from S2 to S3 was significantly greater in the Ca(OH)2 + Ciprofloxacin than the pure Ca(OH)2 and Ca(OH)2 + Ibuprofen groups. In the Ca(OH)2 +Ciprofloxacin group, there were significantly fewer positive cases (8/15) than the pure Ca(OH)2 (13/15) and Ca(OH)2 + Ibuprofen (13/15) groups. |
| Horlenko et al. | (55) | 2020 | Not mentioned | 64 patients | 2 groups ( In the main group the root canals of (n=36) were sonicated in combination with multicomponent antimicrobial gel, in the control (n=35 )teeth were treated by 2% CHX) | Significant reduction of microflora growth and destruction of microbial associations, good penetration of multicomponent antimicrobial gel into endodontic structures due to ultrasound compared with NaOCl were achieved. |
| Carvalho et al. | (56) | 2020 | Interventional trial | 20 patients | Single arm | Chemo-mechanical preparation promoted a drastic reduction in bacterial levels and activity, whereas the adjunctive procedures did not make a significant contribution to further disinfection. At the 2nd visit, bacteria were active after the use of ca(OH)2 medication; however, they were significantly reduced after a 2nd-visit preparation. Consequently, the lowest bacterial levels were found at the end of the treatment. Which highlights the importance of chemomechanical preparation than the ICM |
| Barbosa et al. | (57) | 2020 | Not mentioned | 20 patients | Single arm | After CMP, a significant reduction in the microbial levels by 99.4% was observed. After the use of ICM, the microbial reduction was 99.5% compared to S1. No significant reduction was observed from samples collected after CMP and after ICM. Enterococcus faecalis and Porphyromonas gingivalis were significantly reduced by 10% and Fusobacterium nucleatum by 64.7% after CMP. On the other hand, ICM had no additional effect on the reduction of the bacterial species. The endodontic procedures were effective in reducing the levels of bacteria from teeth presenting with persistent/secondary endodontic infection |
| Ballal et al. | (58) | 2020 | RCT | 80 patients | 4 groups (based on the irrigation activation system either PUI, F-file activation, XP-endo Finisher file activation or needle irrigation after CMP of the canal ) | XP-endo Finisher file activation and PUI were equivalent in terms of reduction in CFUs. F-file activation did not reduce the CFUs to the same extent as the XP-endo Finisher file and ultrasonic activation but reduced the CFUs more than needle irrigation. |
| Aveiro et al. | (59) | 2020 | Not mentioned | 24 patients | 3 groups (control group without activation (WA, n = 8), reciprocating activation group using Easy Clean tip (EC, n = 8) and ultrasonic activation group using Irrisonic insert (US, n = 8).) | PUI was the most effective technique in reducing the concentration and microbial diversity within root canals, followed by reciprocating activation. Both LPS and LTA were found in all initial specimens, but with a significant reduction after chemo-mechanical canal preparation despite the lack of significant difference between the activation protocols. |
| Amaral et al. | (60) | 2020 | Not mentioned | 28 teeth | 2 groups ( group 1, RB (n = 14) and group 2, XP-endo (n = 14).) | XP-endo Shaper and Reciproc Blue systems sharply reduced the bacterial load in ovalshaped root canals of teeth with primary apical periodontitis. The XP-endo Finisher used as a supplementary instrument in the chemomechanical preparation promoted a significantly higher bacterial reduction. |
| Zorita-García et al | (61) | 2019 | Not mentioned | 42 teeth from 33 patients | single arm | Photodynamic therapy as an adjunct to root cananal therapy produced significant reduction in E feaclis bacterial load |
| Savitha et al | (62) | 2019 | RCT | 45 teeth from 28 patients | 3 groups (GP1: 2% CHX gel, GP2: 2% CS gel, GP3: 2% CHX with CS (n=15) per group ) | Maximum reduction in bacterial mean CFU counts was observed between S1 and S2; S2 and S3; S1 and S3 in 2% CHX with CS group compared to other groups. Percentage reduction in CFU counts at different time intervals (S1-S2; S2-S3; S1-S3) was maximum for the 2% CHX with the CS group. Comparison of the mean CFU count within the 2% CHX gel group and the 2% CHX with CS group at different time intervals (S1, S2, S3) was found to be highly significant; whereas in 2% CS, it was significant. 2% CHX with CS group showed the highest microbial reduction against E.faecalis during retreatment of failed endodontic cases. |
| MACHADO et al | (63) | 2019 | RCT | 24 patients | 2 groups (HCM (n = 12); and PTN (n = 12)) | Both HCM and PTN systems were effective in reducing root canal bacteria and endotoxins with primary endodontic infection and that there was no statistical difference between them. However, no system was able to eliminate 100% of the bacteria and their by-products. |
| Thais M. Duque et al | (64) | 2018 | Not mentioned | 10 patients | Single arm | The use of a calcium hydroxide-based ICM showed positive effects for periodontal treatment prognosis, as it reduced LPS, cytokine, and MMP levels in periodontal pockets. |
| Khan Dall at. | (65) | 2019 | Cross-sectional | 30 teeth | 3 groups (Group A(n=10): CH paste (Control Group), Group B(n=10):riple antibiotic paste, and Group C(n=10): double antibiotic paste.) | TAP and DAP not only decreased the bacterial load considerably in infected pulps but also prevented the growth of bacteria in root canal system. An additional benefit of DAP showed a significantly longer residual antibacterial effect compared with the same concentrations of TAP. This property of DAP increases the efficacy of medicament in the infected root canals. |
| Barbosa et al. | (66) | 2019 | Not mentioned | 20 patients | 2 groups (according to the chemical substances used; Group 1 (n = 10): 2% (CHX) & Group 2 (n = 10): 6% (NaOCl). ) | Ca(OH)2 (ICM) has had a positive effect on the microbial reduction by decreasing the levels of PICs and MMPs. Both auxiliary chemical substances (i.e., 2% CHX and 6% NaOCl) presented similar effects when Ca(OH)2 was used as (ICM). |
| Ballal et al. | (67) | 2019 | RCT | 60 patients | 2 groups ( based on irrigation regime. GP1(n=30): 2.5% NaOCl solution only, GP2(n=30): 2.5% NaOCl /9% Dual Rinse HEDP.) | No adverse effects of Rinse HEDP to a2.5% NaOCl solution were detected. The antimicrobial effect of the NaOCl was maintained, whilst no apparent inflammatory effects to the periapical tissues in the form of postoperative pain or an increase in MMP- 9 were added. |
| Sonarkar et al. | (68) | 2018 | RCT | 32 patients | 4 groups (photoactivated disinfection [PAD], diode laser, 5% sodium hypochlorite [NaOCl], and normal saline) | for Aerobic Bacteria, Pad Was More Effective Than 5% NaOClWhereas Diode Laser Was Least Effective. Pad and 5% Naocl Were More Antibacterial Than Diode Laser for Anaerobic Bacteria. |
| Pourhajibagher et al. | (69) | 2018 | Not mentioned | 36 patients | Single arm (TBO-PAD Photo-activated disinfection (PAD) with toluidine blue O (TBO), in combination with diode laser) | Due to the substantial reduction in the microbial diversity and count, following TBO-PAD in root canal infections, our findings suggest that PAD represents an approach with high potential for significant reduction in pathogens involved in the primary endodontic infection. |
| Nakamura et al | (70) | 2018 | RCT | 50 patients | 2 groups (Group UI ultrasonic irrigation (n = 25) and Group NI– needle irrigation (n = 25)) | A significant difference in bacterial levels after each step of the treatment. UI group had lower bacteria levels than NI in S3 samples, whereas no significant differences were found at baseline (S1) and after root canal preparation (S2). After the use of Ca(OH)2, the bacterial counts in S4 became similar in both groups. A significant difference was observed between each step of the treatment for endotoxin levels in both groups. No significant difference was observed regarding endotoxin levels in the intergroup analysis at any step of the treatment |
| Martinho et al | (71) | 2018 | Not mentioned | 72 patients | 6 groups (7-day groups = G1, Ca(OH)2 +saline solution (SSL), G2, Ca(OH)2 + 2% chlorhexidine (CHX) gel, and G3, 2% CHX gel  14-day groups = G4, Ca(OH)2 + SSL, G5, Ca(OH)2 + 2% CHX gel, and G6, 2% CHX gel (n=12) per group ) | All the 7- and 14-day intracanal medications (Ca(OH)₂+ saline solution, Ca(OH)₂+ CHX , CHX gel alone) were effective in reducing bacteria and endotoxins as well as in lowering the levels of inflammatory cytokines, with CHX showing limited effectiveness against endotoxins |
| Khedmat et al | (72) | 2018 | Not mentioned | 40 patients | 4 groups (according to the irrigant used :GP1: 5.25% (NaOCl), GP2: Hypoclean, GP3: 2% CHX and GP4: CHX-Plus. (n=10 ) per group ) | All tested irrigants including 5.25% NaOCl, Hypoclean, 2% CHX and CHX-Plus significantly reduced the number of bacterial colonies in primary endodontic infections. |
| E. Iriboz et al | (73) | 2018 | Not mentioned | 20 patients | Single arm | There were 40.0–99.9% decreases in the numbers of bacteria for all teeth after treatment. The combination of NGS and qPCR techniques resulted in detection of previously unknown components of the oral microbiome. The bacterial community profile before treatment correlated significantly with bacterial reduction, which was unrelated to the community profile after treatment. |
| Miranda et al. | (74) | 2018 | RCT | 32 patients | 2 groups (control(n=16): CMD + intracanal medication and PDT geoup (n=16): CMD + PDT+intracanal medication) | Teeth with apical periodontitis treated with PDT adjunct to conventional chemomechanical preparation would demonstrate superior healing and reduction of microorganisms |
| Silva et al. | (75) | 2018 | Not mentioned | 9 patients | 2 groups (CONTROL GROUP (N=4) 2.5%NaOCl and test group (N=6) PDT) | aPDT may be used as an effective adjunct therapy in the endodontic treatment of permanent teeth, resulting in a signifi cant reduction in the incidence of E. faecalis before root canal obturation at the second session in teeth with primary endodontic infections. |
| Arruda et al. | (76) | 2018 | RCT | 48 patients | 2 groups (according to the ICM used; GP1: TAS at 1 mg/mL ,GP2: Ca(OH)2 in 2% CHX. (n=24) per group ) | ICM with TAP at the concentration of 1 mg/mL significantly improved root canal disinfection, and its effects were at least comparable with the Ca(OH)2 / CHX paste. |
| Rodrigues et al. | (77) | 2017 | Not mentioned | 43 patients | 2 groups (according to the irrigant used (2.5% NaOCl, n = 22; saline, n = 21). | Irrespective of the type of irrigant, an increase in the apical preparation size significantly enhanced root canal disinfection. The disinfecting benefit of NaOCl over saline was significant at large apical preparation sizes. |
| Rabello et al. | (78) | 2017 | RCT | 24 patients | 2 groups ( application of PDT after chemo-mechanical preparation (CMP) in the 1-visit group or the placement of Ca(OH)2 medication in the 2-visit group.) | PDT optimized the disinfection of bacteria from root canals in one-visit but not for two visit treatment modality with the accomplishment of Ca(OH)2 medication. Despite the treatment modality, the supplemental PDT was not effective against endotoxins. |
| Kist et al. | (79) | 2017 | RCT | 60 teeth in 57 patients | 2 groups ( OZONE GROUP AND NaOCL ) | ozone gas (32 g m−3 for 120 s) and the used NaOCl (15 min)/CHX protocols showed no difference in bacterial reduction in the sampled areas within the treatment of apical periodontitis. No differences in the radiographic and clinical outcomes were revealed in the 1-year short-term observation period. |
| Cavalli et al | (80) | 2017 | Not mentioned | 30 patients | 3 groups (according to the instrumentation system (n=10): Rotary Mtwo instruments (n=10), Reciproc reciprocating system (n=10) with a single file, and Genius hybrid instruments with 3 files (1 rotary and 2 reciprocating files)) | The Rotary Mtwo system (8 files), the Reciproc system (single file), and the Genius hybrid system (3 files: 1 rotary and 2 reciprocating) were all significantly effective in reducing both microbial load and endotoxin levels in root canals with primary endodontic infections. However, no statistically significant differences were observed among the three instrumentation systems |
| Asnaashari et al | (81) | 2017 | RCT | 20 patients | 2 groups ( Group 1: Photodynamic therapy( PDT), Group 2: Calcium hydroxide) | A significant difference was observed between the results of pre- and post-treatment in calcium hydroxide therapy group and Photodynamic therapy group. The post-treatment comparison showed that photodynamic therapy was significantly more effective. |
| Zandi et al | (82) | 2016 | RCT | 49 patients | 2 groups. (NaOCl, n = 20; CHX, n = 29). | No significant difference between NaOCl and CHX was found.NaOCl and CHX both reduced bacterial counts and the number of infected canals. Ca(OH)2 ICM reduced the number of canals with persistent infection but resulted in overall larger bacterial counts in the cases positive for bacteria. |
| Isabela N. R^oc et al | (83) | 2016 | RCT | 50 patients | 2 Groups (2.5% NaOCl (n = 25) or 2% CHX (n = 25)) | After CMP using either 2.5% NaOCl or 2% CHX, 44% and 40% of the root canals still had detectable bacteria, respectively. The differences in both the presence/absence and quantitative data were not statistically significant. Both irrigation protocols were highly effective in reducing the levels of Streptococcus species. No significant difference was observed for the clinical antibacterial effectiveness of CMP using either 2.5% NaOCl or 2% CHX as the main irrigant. |
| Romano et al. | (84) | 2016 | RCT | 120 patients | 4 groups (Group A: 5.25% sodium hypochlorite (NaOCl) + EndoActivator®; Group B: 5.25% NaOCl + IRRI S® files; Group C: 2% chlorhexidine gluconate (CHX) + EndoActivator®; Group D: 2% CHX + IRRI S® files.) | No significant differences were observed between NaOCl and CHX in the reduction of CFU; in fact, reduction was > 93% for the two irrigants. Conversely, statistically significant differences were found between the two activation techniques (sonic and ultrasonic) in the reduction of Enterococcus faecalis (EF). Thus, the effectiveness of ultrasonic activation was significantly higher (> 93%; p=0.012) as compared to sonic activation. Following the combination of the two irrigants with the two activation techniques (groups A, B, C and D), significant differences were observed between group A and B (p=0.025) in the reduction of EF populations, reaching up to 94%. |
| Neves et al. | (85) | 2016 | Not mentioned | 59 teeth | 2 groups(group 1 Reciproc group(n=29)and group 2 biorace (n=30)) | After preparation with Recip roc and BioRaCe, 16 (55%) and 15 (50%) root canals still had detectable bacteria with median counts of 7.05 102 and 6.03 101, respectively. Both systems were highly effective in reducing the total bacterial counts (P < .001), and there were no significant differ ences between them (P > .05). Streptococci were highly frequent, and both systems succeeded in significantly reducing their levels (P < .001). |
| Ghoneim et al. | (86) | 2016 | Not mentioned | 40 patients | 2 groups (according to health condition being systemically normal (Group A) or diabetic (Group B), then they were subdivided according to irrigation methods used during CMP to conventional syringe groups (A1 & B1) and EndoVac groups (A2 & B2) ) | The EndoVac irrigation system was effective in reducing bacteria, especially Peptostreptococcus micros in the diabetic group when compared to conventional irrigation technique with a statistically significant difference. |
| Donyavi et al. | (87) | 2016 | RCT | 30 teeth | 2 groups (GP 1: Root canal was irrigated with 2 mL of 6% NaOCl (single visit), GP 2: a mixture of 0.2% CHX and Ca(OH)2 powder as an ICM for two weeks (two visit), (n=15) per group) | The percentage of reduction in E. faecalis count was not significantly different between the single and two visit but the percentage of reduction in aerobes and anaerobes was significantly higher in the two visit group (CHX+ Ca(OH)2) compared to the single visit group. |
| Ribeiro et al. | (88) | 2016 | Not mentioned | 20 patients | 2 groups (according to the chemical substance used for CMP: chlorhexidine (CHX) group, 2% CHX gel, and the sodium hypochlorite (NaOCl) group, 6% NaOCl, (n = 10 per group)) | The reduction rates of bacteria were higher than the LTA. Moreover, gram-positive microorganisms were present in all phases of the endodontic retreatment. |
| Asnaashari et al | (89) | 2016 | CT | 20 patients | 2 groups (Group 1: Photodynamic Therapy (PDT) (n=10) Group 2: Diode Laser (n=10)) | CFU/mL amounts showed a statistically significant reduction within the PDT and Diode laser groups. No statistically significant difference was found when comparing CFU/mL values between the two groups. |
| Zeledón et al | (90) | 2015 | Not mentioned | 56 patients | Single arm | Endodontic protocol treatment significantly reduced the number of cases with positive results for the studied bacteria, The most frequently detected bacterial species after chemomechanical preparation, in descending order of prevalence, were Actinomyces israelii, Enterococcus faecalis, Fusobacterium nucleatum, Prevotella nigrescens, and Porphyromonas endodontalis. 6 unsuccessful cases showed the persistence of bacteria in S4 samples, proving the influence of residual bacteria on the outcome of endodontic treatment. At the 18-month radiographic follow-up, the overall success rate of root canal–treated teeth was 88%, based on Periapical Index (PAI) scores of 1 or 2. |
| Rodrigues et al | (91) | 2015 | Not mentioned | 43 patients | 2 groups (according to the instrumentation system used; GP 1: SAF (n=21), GP2: TFA with or without PUI (n=22)) | Both SAF and TFA instrumentation protocols showed a highly significant intracanal bacterial reduction. Intergroup quantitative comparisons disclosed no significant differences between TFA with or without PUI and SAF. PUI did not result in significant improvement in disinfection. The mean total bacterial reduction was 83.7% for SAF, 94.8% for TFA, and 96.9% for TFA + PUI. |
| Provenzano et al | (92) | 2015 | Not mentioned | 18 patients | 2 groups ( 2.5% NaOCl (9 teeth) / 2% CHX (9 teeth)) | all S1 samples were positive for total bacteria. Overall, 12 of 17 S2 samples (71%) and 8 of 17 S3 samples (47%) still had detectable bacterial levels. In NaOCl group, 6 of 9 S2 samples (67%) and 4 of 9 S3 samples (44%) exhibited positive PCR results for bacteria, whereas respective figures for the CHX group were 6 of 8 S2 samples (75%) and 4 of 8 S3 samples (50%). Butyric acid was the most common fatty acid in S1, followed by propionic acid. Both molecules were also found in S2 and S3 from both NaOCl and CHX irrigation. Lactic acid was not present in detectable levels in S1, but it occurred in 1 post CMP sample and in 9 samples taken after Ca(OH)2 ICM. Of the target taxa, Fusobacterium nucleatum was the most prevalent in S1 (76%), followed by members of the Actinobacteria phylum (71%), Streptococcus species (59%), and Parvimonas micra (53%). Gram-positive taxa, especially streptococci, were the most prevalent bacteria in S2 and S3. |
| Martinho et al. | (93) | 2015 | Not mentioned | 30 patients | 3 groups (according to the intracanal medication selected: CHX, 2% CHX gel; Ca(OH)2/SSL, Ca(OH)2 + SSL; and Ca(OH)2/CHX, Ca(OH)2 + 2% CHX gel (n = 10 per group ).) | all intracanal medication protocols wereeffective in reducing bacterial load and lowering the levels of Th1-type cytokines. Thus, the use of Ca(OH)2 medications contributed to the increase in the Th2-type cytokine response in apical periodontitis. |
| Martinho et al. | (94) | 2015 | Not mentioned | 30 teeth | 3 groups (according to the system used: WaveOne (n=10),Reciprocinstrument(n=10), and ProTaper Universal Retreatment system (n=10).) | After CMP, no differences were found in the median percent age values of endotoxin reduction achieved with reciprocating systems—WaveOne [94.11 %] and Reciproc [93.29 %] and with rotary systems—ProTaper [94.98 %] (P>0.05). Both single-file reciprocating systems [WaveOne (98.27 %) and Reciproc (99.54 %)] and rotary system [ProTaper (98.73 %)] were effective in reducing bacterial load (P>0.05). Moreover, no differences were found among the systems tested. |
| Marinho et al. | (95) | 2015 | Not mentioned | 30 patients | 3 groups (according to the irrigant used for root canal preparation: GI: 2.5% NaOCl (n = 10), GII: 2% CHX gel (n = 10), and GIII (control group): saline solution (n = 10).) | Regardless of the use of NaOCl or CHX, the greatest endotoxin reduction occurs after CMP. Increasing steps of root canal therapy associated with ICM enhances endotoxin reduction, leading to a progressively lower activation of proinflammatory cells such as macrophages. |
| Ferreira et al. | (96) | 2015 | Not mentioned | 20 patients | 2 groups (according to the intracanal medications: calcium hydroxide (Ca[OH]2) or Ca(OH)2 + chlorhexidine (CHX)) | No differences were found between the Ca(OH)2 (99.98%) and Ca(OH)2 + CHX groups (99.76%) regarding the median percentage values for the reduction of cultivable bacteria. The most frequently detected species were Capnocytophaga ochracea (70%) and Fusobacterium nucleatum ssp. vincentii (70%) in the initial samples. After instrumentation, the most frequently detected species were E. faecium (60%). After root canal treatments using either Ca(OH)2 or Ca(OH)2 + CHX as intracanal medications, the most frequently detected species were F. nucleatum ssp. vincentii (90%) and Enterococcus faecium (40%), respectively. Both treatments significantly decreased the number of bacterial species compared with the initial sample. However, this reduction was significantly greater in the Ca(OH)2 + CHX group (P < .05). This difference was also observed when evaluating the total bacterial load (P < .05). Conclusions: The use of Ca(OH)2 associated with CHX as an intracanal medication showed better results by acting on grampositive and gram-negative microorganisms although such an action to eradicate enterococci should also be sought. |
| Adl et al. | (97) | 2015 | Not mentioned | 25 patients | Single arm | endotoxin level in the infected root canals was reduced significantly after root canal preparation and further reduced after 7 days of dressing of canals with Ca(OH); however, these procedures were not able to render the canals free of LPS, and relatively high values of endotoxin remained in the root canals. |
| Telesa et al | (98) | 2014 | RCT | 69 patients | 2 groups ( Group 1: Calcium hydroxide paste (Ca(OH)₂), (n = 35) Group 2: 2% Chlorhexidine gel (CHX), (n = 34)) | CMP was highly effective in reducing the bacterial load within necrotic root canals. Ca(OH)₂ showed superior outcome compared to 2% chlorhexidine gel (CHX). CHX was associated with a significant increase in bacterial load across all atmospheric conditions. This finding was pronounced in teeth diagnosed with apical periodontitis, where Ca(OH)₂ significantly reduced bacterial levels from S1 to S3, whereas CHX allowed bacterial regrowth. At the final sampling point (S3), Ca(OH)₂-treated canals had significantly lower bacterial counts than those treated with CHX. |
| Stojanović et al | (99) | 2014 | Not mentioned | 51 patients | 3 groups (according to the ICM t used: Ca(OH)2, Ca(OH)2-GP and CHX-GP group (n=17 per group)) | All tested ICM showed significant difference in the number of PCR positive samples between S1 and S2, S1 and S3, but not between S2 and S3 samples. E. faecalis is more prevalent than P. gingivalis in primary endodontic infection. ICM in conduction with CMP efficiently eliminates E. faecalis and P.gingivalis from infected root canals. |
| Isabela N. R^oc et al | (100) | 2014 | Not mentioned | 28 teeth from 23 patients | Single arm | CMP promoted a highly statistically significant reduction in total bacterial counts, but 64% of the canals were still positive for bacterial presence. Of the target taxa, only Bacteroidaceae sp. HOT-272 and F. fastidiosum were detected in S2. CMP was highly effective in substantially reducing their numbers. |
| Neves et al. | (101) | 2014 | Not mentioned | 44 teeth | 2 groups (G1:SAF and G2 :2.5%NaOCl) | SAF significantly reduced the total bacterial counts (P < 0.001). Quantitatively, the 99.9% reduction in total bacterial counts associated with the SAF system was significantly superior to the 95.1% reduction obtained by hand instrumentation (P < 0.001). Qualitatively, SAF resulted in significantly more cases with negative PCR results for bacteria (54.5%) than hand instrumentation (4.5%) (P < 0.001). The SAF system succeeded in sig nificantly reducing the streptococcal levels, but four cases still harboured these bacteria in S2. Checkerboard analysis revealed that not only streptococci but also some anaerobic and even as-yet-uncultivated bacteria may resist the effects of chemomechanical procedures. |
| Mashalkar et al. | (102) | 2014 | Not mentioned | 60 teeth | 2 groups (Group A: In this group, laser was used to disinfect  the canal followed to conventional biomechanical preparation using normal saline as irrigant (n=30)  Group B: These teeth were disinfected with the  conventional technique using NaOCl and H2 O2 as irrigating solutions (n=30)) | Conventional method by using NaOCl and H2O2as irrigating solutions is highly effective in disinfecting the root canal. Lasers when used can also reduce the bacterial load of the infected root canal.O22 |
| Martinho et al | (103) | 2014 | Not mentioned | 48 patients | 4 groups (WaveOne (n = 12), Reciproc (n = 12), ProTaper (n = 12), and Mtwo (n = 12). ) | Both single-file reciprocating systems (ie, WaveOne and Reciproc instruments) and rotary systems (ie, ProTaper and Mtwo instruments) showed similar effectiveness in reducing endotoxins and cultivable bacteria from primarily infected root canals, but they were not able to eliminate them from all root canals analyzed. |
| Juric et al. | (104) | 2014 | Not mentioned | 21 teeth | Single arm | Fourteen bacteria species were isolated from the root canals initially. Although endodontic re-treatment alone produced a significant reduction in the number of bacteria species, the combination of endodontic treatment and PDT was statistically more effective. |
| Xavier et al. | (105) | 2013 | Not mentioned | 48 patients | 4 groups ( G1, 1% NaOCl (n = 12); G2, 2% CHX gel (n = 12); G3, 1% NaOCl + calcium hydroxide [Ca(OH)2] (n = 12) medication; and G4, 2% CHX gel + [Ca(OH)2] medication (n = 12), with the first 2 groups involving 1-visit treatment and the latter 2 involving 2-visit treatment.) | both 1-visit and 2-visit root canal treatment protocols were effective in reducing bacteria and endotoxins, but they were not able to eliminate them in all root canals analyzed. Furthermore, 2-visit root canal treatment protocols were more effective in reducing endotoxins than 1-visit root canal treatment protocols. |
| Rôças et al. | (106) | 2013 | Not mentioned | 40 patients | 2 groups (hand files (n = 20) or with rotary NiTi instruments (n = 20).) | no significant difference in bacterial reduction in infected canals after instrumentation using hand or rotary NiTi instruments, provided canal enlargement and irrigation parameters are similar. In terms of incidence of positive results for bacteria, culture also showed no significant differences between the groups, but the rotary NiTi instrumentation resulted in more negative results in the more sensitive qPCR analysis |
| Provenzano et al | (107) | 2013 | Not mentioned | 12 teeth with asymptomatic apical periodontitis, and 2 cases of acute apical abscess | 2 groups (2.5% NaOCl (6 teeth), 2% CHX (6 teeth), 2 cases of acute apical abscesses (analyzed individually)) | All S1 samples (collected from root canals of teeth with AAP) and abscess samples tested positive for bacteria, confirming widespread bacterial presence before treatment. In the post-instrumentation (S2) samples, 4/6 canals irrigated with 2.5% sodium hypochlorite (NaOCl) and 5/6 canals irrigated with 2% chlorhexidine (CHX) still showed positive PCR results, S2 samples showed a decrease in the number of identified proteins, but an increase in the NaOCL group. A total of 308 microbial proteins were identified in the study. A larger number of proteins were identified in abscess samples (173 proteins) compared to S1 samples from teeth with AAP (88 proteins). |
| Paiva et al | (108) | 2013 | Not mentioned | 10 patients | single arm | The supplementary PUI approach did not succeed in significantly enhancing disinfection. Several bacterial species/phylotypes were identified in post-treatment samples that were positive for bacteria |
| Paiva et al | (109) | 2013 | Not mentioned | 14 patients | Single arm | All S1 samples were positive for bacteria. CMP promoted a decrease in microbial diversity and significantly reduced the incidence of positive results and the bacterial counts. Supplementary steps consisting of a final rinse with CHX followed by Ca(OH)2 ICM promoted further decrease in the bacterial bioburden to levels significantly below those achieved by the CMP alone. |
| Halbauer et al. | (110) | 2013 | Not mentioned | 37 root canals from 23 teeth in 20 patients | Single arm | the decrease in number of bacteria after the treatment with ozone was highly statistically significant: p<0.001 for aerobic, and p<0.001 for anaerobic bacteria. A decrease in the total number of bacteria of 82% (Z=–4.826, p<0.001) was found: 67% of aerobic, or 93% of anaerobic bacteria respectively. |
| M. S. Endo et al. | (111) | 2013 | Not mentioned | 15 patients | 3 groups (according ICM; Group 1 (n = 5): Ca (OH) 2 + 2% CHX gel Re‑accessed after 14 days, Group 2 (n = 5): Ca (OH) 2 + sterile physiological solution (0.9% NaCl) Re‑accessed after 14 days and Group 3 (n = 5): 2% CHX gel Re‑accessed after 7 days.) | The great majority of taxa found in post‑treatment samples were Gram‑positive bacteria, although Gram‑negative bacteria were found by molecular methods. Moreover, results showed that gutta‑percha removal and CMP are effective for root canal disinfection, whereas additional ICM did not improve disinfection. |
| Cohenca et al. | (112) | 2013 | RCT | 32 patients | 4 groups (based on instrumentation and irrigation technique as follows; GP 1: LightSpeed LSX 0.02 (non-tapered) and ANP irrigation, GP 2: LightSpeed LSX 0.02 (non-tapered) and PP irrigation, GP 3: ProTaper (tapered) and ANP irrigation, GP 4: ProTaper (tapered) and PP irrigation.(n=8) per group ) | Taper and apical size failed to demonstrate a difference in microbiological reduction of cultivable bacteria. On the other hand, ANP revealed a significant difference when compared to PP irrigation. |
| Endo et al. | (113) | 2012 | Not mentioned | 15 Patients | Single arm | levels of endotoxin found in infected root canals were related to a larger size of the radiolucent area in the periapical region. Moreover, chemomechanical preparation with 2% CHX+17 % EDTA was effective in reducing both bacterial load and endotoxin contents in the post-treatment apical periodontitis. |
| Rôças et al | (114) | 2011 | Not mentioned | 50 patients | 2groups (2.5% NaOCl solution (30 canals) , 0.12% CHX solution (20 canals)) | Both NaOCl- and CHX-based protocols were significantly effective in reducing the bacterial levels and number of taxa. No significant differences were observed between them in all tested parameters, including the incidence of negative PCR results in S2, reduction in the number of taxa per canal, and reduction in the bacterial levels. The most prevalent taxa in S2 samples from the NaOCl group were Propionibacterium acnes, Streptococcus species, Porphyromonas endodontalis, and Selenomonas sputigena. In the CHX group, the most prevalent taxa in S2 were Dialister invisus, Actinomyces israelii, Prevotella baroniae, Propionibacterium acidi faciens, and Streptococcus species. |
| Rôças et al | (115) | 2011 | Not mentioned | 27 patients | 2 groups (CHG group: Calcium hydroxide in glycerin (n = 12), CHPG group: Calcium hydroxide with camphorated paramonochlorophenol/ glycerin (n = 12)) | In the CHG group, 50% of canals were bacteria-free after cleaning, increasing slightly to 58% after medication. In the CHPG group, bacterial clearance improved from 42% to 67%. Although both protocols effectively reduced bacterial presence, the difference between them was not statistically significant. Propionibacterium acnes and Streptococcus species were the most commonly detected bacteria after treatment. |
| Martinho et al | (116) | 2010 | Not mentioned | 21 patients | 2 groups according to irrigation: control group (sterile saline) & test group (2.5% NaOCl, 17% EDTA) | CMP with 2.5% NaOCl + 17% EDTA and rotary NiTi files was effective in reducing endotoxin load in the root canal infection from primarily infected teeth with AP. |
| Abbaszadegan et al. | (117) | 2010 | Not mentioned | 30 teeth | 2 groups ( G1:2.5%NaOCl and G2: saline) | Though the initial CFU count of canals which were irrigated with NaOCl had decreased about by 90%; viable microorganisms were still detectable by culturing in post instrumentation samples. In IKI group, only 15% CFU reduction compared to the first sample was achieved after instrumentation and irrigation. There were no bacteria free cultures in S2. |
| Malkhassian et al. | (118) | 2009 | RCT | 30 teeth | 2 groups (according to the final rinse of the canal MTAD or saline ) | The vital bacterial counts (both CFU and EFM values) decreased substantially from 1A to 1B samples. In both samples 1A and1B, the counts did not differ significantly between the MTAD and Control groups. The EFM-based density of live bacteria at the end of the second treatment session, after additional irrigation with 1.3% NaOCl (2B samples), was not significantly different from that in the end of the first treatment session. The results suggested that the second treatment session did not improve the overall antibacterial effectiveness beyond what was achieved in the first treatment session. |
| Bebek et al. | (119) | 2009 | Cohort | 44 patients | 2 groups (the experimental group: irrigation with 0.2% CHX (n=25) and the control group: irrigation with saline (n=19).) | There was statistically significant reduction of microorganisms (65.46%) after irrigation with 0.2% CHX. In the control group there was insignificant reduction of microorganisms. At the second appointment (48h) ,the experimental group yielded 65% positive subjects as compared with 89% positive subjects in the control group. |
| Vianna et al. | (120) | 2008 | Not mentioned | 24 teeth | 3 groups ( ACCORDING TO INTRACANAL MEDICATION : [M1: Ca(OH)2 paste; M2: 2% CHX gel; and M3: Ca(OH)2 paste plus 2% CHX gel]) | The antibacterial effect of the mechanical preparation supplemented by the use of an antibacterial auxiliary substance largely reduced the microorganisms in the main root canal. The highest bacterial amount was found in the initial samples (S1). After chemomechanical preparation using 2% CHX gel as auxiliary substance (S2), a major reduction in bacteria was achieved. On the other hand, the use of an intracanal dressing for 7 days did not improve the bacterial reduction achieved by chemo-mechanical preparation. Moreover, it contributed to a slight increase in bacterial numbers and in the number of positive cases. |
| Martinho et al. | (121) | 2008 | Not mentioned | 24 teeth | Single arm | Even though the present study demonstrated that chemomechanical preparation with 2.5% NaOCl was able to reduce LPS content by only 59.99%, a successful endodontic treatment can still be achieved in clinical practice. However, high contents of LPS might play an important role in the development and the persistence of endodontic symptoms after root canal treatment. chemomechanical preparation with 2.5% NaOCl was moderately effective against bacteria but less effective against endotoxins in root canal infection. Furthermore, a statistically significant association was found between higher levels and clinical symptomatology. |
| Garcez et al | (122) | 2008 | Not mentioned | 20 root canals | Single arm | The combination with PDT significantly enhanced the reduction. The second endodontic session gave a similar diminution to the first, and the second PDT was significantly more effective than the first. The second total reduction was significantly higher than the second endodontic therapy. The total first + second reduction was significantly different from the first combination. |
| Garcez et al | (123) | 2008 | Not mentioned | 15 patients | 2 groups (Group 1: (N=5) received endodontic treatment, and Group 2: (n=10) received endodontic treatment associated with PDT) | both conventional endodontic therapy and its combination with photodynamic therapy (PDT) significantly reduced microbial load and contributed to the healing of periapical lesions. Conventional endodontic treatment alone achieved an 87% decrease in CFUs, whereas the combination with PDT resulted in a significantly greater reduction of 95%. Radiographic follow-up at 6 months demonstrated a 46% reduction in lesion size for the conventional group and a 68% reduction in the PDT group. |
| Blome B et al | (124) | 2008 | Not mentioned | 40 patients | 2 groups (according to the clinical diagnosis; GP1: primary infection- 20 untreated teeth with AAP, GP2: secondary infection – 20 root-filled teeth with AAP ) | Root canals with primary infections harbored significantly more bacteria than teeth with secondary infections. CMP significantly reduced bacterial counts and their detection frequency in both primary and secondary infections. The use of CA(OH)2 ICM for 14 days did not lead to a further reduction in the total bacteria counts, although the detection frequency of individual bacterial species was further reduced. |
| Ching S et al | (125) | 2007 | Not mentioned | 43 patients (test subjects), 4 patients (negative control subjects) | Single arm | 2% CHX gel showed significant difference in the percentage of positive culture between S1 and S2 but not between S2 and S3. Ca(OH)2/2% CHX gel ICM for at least 2 weeks rendered 91.7% of canals bacteria free in teeth with AP. |
| Vianna ME | (126) | 2007 | Not mentioned | 24 teeth | 3 groups (according to the type of ICM [CaOH2 paste; 2% CHX gel; and CaOH2 + 2% CHX gel].) | Relatively high values of endotoxin were still present in the root canal after chemo-mechanical preparation with 2% CHX,although the majority of bacteria were eliminated. No improvement was achieved by 7 days of 3 different ICM techniques; Ca(OH)2 paste; 2% CHX gel; and Ca(OH)2 + 2% CHX gel. |
| Siqueira et al | (127) | 2007 | CT | 32 teeth | 2 groups ( according to the type of irrigant G1:2.5%NaOCl and G2:.0.12%CHX) | bothNaOCl and CHX were significantly effective in reducing bacterial populations within the canals (P .001 for both groups). Intergroup analysis of quantitative data from S2 samples showed no significant difference between NaOCl and chlorhexidine. Comparison of the number of cases yielding negative cultures in the 2 groups did not revealasignificant difference either |
| Siqueira et al. | (128) | 2007 | Not mentioned | 13 teeth | Single arm | CMP with 0.12% CHX solution as an irrigant significantly reduced the number of intracanal bacteria but failed to render the canal free of cultivable bacteria in about one half of the cases. Application of a 7-day intracanal dressing with Ca(OH)2/CHX paste further increased significantly the number of cases yielding negative cultures. |
| Karen et al. | (129) | 2007 | Not mentioned | 11 teeth | Single arm | CMP with 2.5% NaOCl as an irrigant significantly reduced the number of bacteria in the canal but failed to render the canal free of cultivable bacteria in more than one-half of the cases. A 7-day intracanal dressing with Ca(OH)2/CPMC paste further significantly increased the number of culture-negative |
| Siqueira Jr et al. | (130) | 2007 | Not mentioned | 12 teeth | Single arm | a statistically significant reduction in the number of cultivable bacteria from infected root canals was obtained following the protocol used. No cultivable bacteria were isolated from 54.5% of the canals after instrumentation with hand NiTi files and irrigation with 2.5% NaOCl. The number of cases showing negative cultures further increased to 81.8% after smear layer removal and a 7-day intracanal medication with calcium hydroxide in an inert vehicle (glycerin). Although this difference did not reach statistical significance, it appears that an increase of almost 30% in the number of culture-negative cases would be reasonable to justify the use of calcium hydroxide medication. Even so, the fact that some cases still harbored bacteria after the whole treatment protocol points to the need to develop more effective strategies to predictably render canals bacteria free. |
| Schirrmeister et al. | (131) | 2007 | Not mentioned | 20 patients | Single arm | Because all samples were negative after irrigation with NaOCl and EDTA in the present study, CHX could not show an additional effect. Under the conditions of the present study, it may be stated that CHX as an additional irrigant is not necessary after copious irrigation using NaOCl and EDTA. Because no microorganisms were detected at the end of the first appointment, no conclusion about the effectiveness of Ca(OH)2 can be drawn. However, at the second appointment, two teeth (10%) showed positive culture. This fact may be explained by a leaking restoration after placement of the root canal dressing. Although the temporary restoration was placed using composite in combination with an adhesive, a reinfection appeared. The polymerization shrinkage of the composite might have caused partial debonding, and thereby leakage occurred. proper removal of the root canal filling material, proper root canal preparation, and irrigation using a copious amount of NaOCl and EDTA are sufficient for decontamination of the root canal system during endodontic retreatment. However, care should be taken when interpreting the results because a negative culture or PCR result does not say that there are no microorganisms present. They may be present in the apical ramification and be covered by remaining root canal filling material, not be soaked up by the paper point and thereby not be detected, or they may even be unculturable. |
| Sakamoto et al | (132) | 2007 | Not mentioned | 18 teeth | single arm | Following chemomechanical preparation with 2.5% sodium hypochlorite (S2), the mean bacterial load decreased significantly compared to pre-instrumentation levels (S1). After one week of intracanal medication with calcium hydroxide, camphorated paramonochlorophenol (CMCP), or glycerin (S3), the bacterial load showed a further significant reduction compared to baseline. However, the difference between post-instrumentation (S2) and post-medication (S3) bacterial levels was not statistically significant. |
| Paquette et al | (133) | 2007 | Not mentioned | 22 teeth | single arm | Following chemomechanical preparation with 2.5% sodium hypochlorite (sample 1B), a statistically significant reduction in bacterial counts was observed compared to the initial baseline levels (sample 1A). However, after 7 to 15 days of intracanal medication using 2% chlorhexidine (CHX) solution (sample 2A), bacterial counts increased significantly relative to the post-instrumentation levels, indicating bacterial regrowth during the medication period. A subsequent disinfection step (sample 2B), which involved final irrigation with sodium hypochlorite, sodium thiosulfate, and saline, resulted in a reduction of bacterial counts. These levels were not significantly different from those achieved immediately after chemomechanical preparation (sample 1B). |
| Manzur et al | (134) | 2007 | RCT | 33 patients | 3 groups (according to ICM used , 11 in each, Gp1 : Ca(OH)2, GP2: 2% CHX gel, GP3: Ca(OH)2 / 2% CHX.) | Bacterial growth and CFU counts decreased significantly from S1 to S2. Differences in growth and counts between S2 to S3 were not statistically significant for all three ICMs. |
| Carver et al | (135) | 2007 | Not mentioned | 31 patients | 2 groups ( GP1(n=16) prepared with a manual hand-file/rotary CMP but with no ultrasonic irrigation. GP2 : (n=15)prepared with manual hand-file/rotary CMP , followed by 1 minute of ultrasonic irrigation per canal with 6.0% NaOCl.) | Hand/rotary CMP significantly reduced the number of bacteria from initial counts. The addition of 1 minute of ultrasonic irrigation resulted in significant reduction in CFU count and positive cultures. This system was shown to remove vital tissue from canals and significantly better than hand and rotary CMP alone |
| Vianna et al. | (136) | 2006 | Not mentioned | 32 patients | 2 groups (according to the irrigant type either NaOCl or CHX) | As measured by RTQ-PCR, the bacterial reduction in the NaOCl-group was significantly greater than in the CHX-group. According to CFU 75% of cases were free of bacteria after chemo-mechanical preparation in the NaOCl-group, whilst 50% of cases were bacteria free in the CHX-group. |
| Soares et al. | (137) | 2006 | CT | 93 teeth from 83 patients | 3 groups ( I (n=39): 1%NaOCl, II (n=36):2.5%NaOCl and III (n=36): 5% NaOCl, with 31, 32 and 30 teeth) | All teeth with 2 root canals and/or associated fistulas were microbiologically negative after BMP, regardless of irrigant concentration. After irrigation with 5% NaOCl, only structural arrangements consisting of Gram-positive cocci and bacilli persisted. Thus, BMP plus 5% NaOCl offered the best antiseptic potential because in the few positive cultures a significant reduction in the number of microbiological morphotypes was also shown. |
| Chu et al. | (138) | 2006 | Not mentioned | 88 canals in 87 patients | 2 groups (45 teeth with exposed pulp space, and 43 teeth with unexposed pulp space.Canals in each group were randomly divided into three sub-groups for standard CMP, irrigation, and dressing with one of the following: Ledermix, Septomixine forte, or Calasept.) | Endodontic treatment with different medicaments could markedly affect the diversity and quantity of cultivable microorganisms in infected canals, with some groups of microorganisms more resistant to treatment than others. It was also noted that CMP with the use of the antibiotic/steroid combinations or Ca(OH)2 as ICM produced similar microbiological outcomes. |
| McGurkin-Smith et al. | (139) | 2005 | Not mentioned | 31 test subjects and 4 negative control subjects | Single arm | GT protocol significantly reduced the number of bacteria in the canal but failed to render the canal bacteria free in more than half of the cases Ca(OH)2 application significantly further reduced bacteria. Lastly, large apical instrumentation removed more bacteria than small apical instrumentation. |
| Ferrari et al. | (140) | 2005 | Not mentioned | 25 patients | Single arm ( three visit treatment with 0.5% sodium hypochlorite solution, 10 mL of SDS-EDTA solution, 7 days with temporary without intracanal medication, then paramonochlorophenol [PRP (2.0 g), Rinosoro and polyethylene glycol (400 equal parts up to 100 mL)] as an intracanal dressing for 7 days) | Microorganisms were isolated from 92% of the samples following intracoronal access, 22% were enterococci, enteric bacteria or yeast species. After biomechanical preparation, these species were no longer detected. After 7 days without intracanal dressing, 100% of the canals contained microorganisms, 52% of which were target species. However, after using paramonochlorophenol [PRP (2.0 g), Rinosoro and polyethylene glycol (400 equal parts up to 100 mL)] as an intracanal dressing for 7 days, enteric bacteria and yeasts were not detected; only enterococci were still present. All strains of enterococci were susceptible to ampicillin, but exhibited variable susceptibility to rifampin and ciprofloxacin.Enterococci, enteric bacteria and yeasts were present in primary endodontic infections; enterococci were the most frequently isolated. Enterococci, particularly E. faecalis and E. faecium, were more resistant to root canal preparation and intracanal dressing |
| Souza et al. | (141) | 2005 | Not mentioned | 12 patients | Single arm | great microbial diversity in cases of pulp necrosis with periradicular lesions, confirming the polymicrobial cause of these infections. Fastidious species not previously related to these infections, such as P. gingivalis, T. forsythensis, and T. denticola were observed in high prevalence and levels. In addition, calcium hydroxide proved to be an effective adjunctive in mechanical endodontic therapy. Nevertheless, its use is limited, given that it decreases but does not completely eliminate the microorganisms from the root canal system. Future investigations should be conducted to define the role of specific species in the development of infections of endodontic origin, as well as to search for new adjunctive medications in endodontic treatment. |
| Kvist et al | (142) | 2004 | RCT | 96 teeth in 85 patients | 2 groups (One Visit (n=52), Two visits(n=44)) | Both the one-visit group (treated with a 10-minute IPI ) and the two-visit group (treated with Ca(OH)2 for one week) showed a reduction in bacterial counts, with no significant difference between them overall. |
| Ercan et al | (143) | 2004 | Not mentioned | 30 canals in 20 patients | 2 groups (Group 1 (n = 15): 2% chlorhexidine gluconate, Group 2 (n = 15): 5.25% sodium hypochlorite) | CHXwas significantly more effective than NaOCl for both sampling periods, immediate post-instrumentation and 48-hour post-instrumentation time points |
| Peters et al | (144) | 2002 | Not mentioned | 43 patients | 2 groups (GP 1 one visit and GP two visit with Ca(OH)2 ICM ) | Although a Ca(OH)2 ICM was placed in the prepared canals, the number of positive canals had increased in the period between visits. However, the number of microorganisms had only increased to 0.93% of the original number of CFU (sample 1). Ca(OH)2 and sterile saline slurry limits but does not totally prevent regrowth of endodontic bacteria. |
| TATJANADOSTÁLOVÁ et al. | (145) | 2002 | Not mentioned | 44 patients | 2 groups (G1: 10 teeth were treated with Ca(OH)2 paste, and G2 (n=22) irradiated by ER-YAG LASER) | Classical enlargement and shaping of the root canal, including chemical disinfections, was effective in 60% of premolars and molars. Application of Ca(OH)2 can prepare sterile root canal in 80% of teeth. Application of Er:YAG laser radiation through a movable waveguide appeared to be effective for root canal residual disinfections. With the application of a 30-pulse dose with a mean energy of 100 mJ (repetition rate 4 Hz) a 100% sterilization was reached. |
| Peciuliene et al. | (146) | 2001 | Not mentioned | 40 patients | 2 groups (group A the canals were filled with Ca(OH)2 for 10–14 days after CMP (n=20) ; in group B the canals were irrigated with IKI for 5 min after CMP (n=20).) | The high prevalence of enteric bacteria and yeasts in root-filled teeth with chronic AP was established. IKI improved the antimicrobial effect of the treatment. |
| Shuping et al. | (147) | 2000 | Not mentioned | 42 Teeth | 2 groups ( group 1 0.04 NiTi rotary instrumentation with 1.25% NaOCl irrigation compared with group 2 saline irrigation). | statistically significant difference in bacterial reduction between the initial sample and the progressive filing to final instrumentation with 0.04 NiTi rotary files and NaOCl irrigation. Instrumentation with NaOCl irrigation was superior in bacterial reduction to instrumentation with sterile saline and resulted in 61.9% of canals becoming free of bacteria. An increase in file size was shown to be important in allowing the NaOCl to be an effective antibacterial irrigant. The addition of Ca(OH)2 as an ICM for at least 1 week produced 92.5% of canals void of bacteria. There was a statistically significant decrease in bacterial numbers between the final instrumentation samples and the samples taken after calcium hydroxide therapy. |
| Dalton et al. | (148) | 1998 | Not mentioned | 48 test subjects and 5 negative control subjects | 2 groups ( NiTi rotary files (ProFile .04) and a stainless-steel K-files with a step-back technique.) | NiTi rotary and stainless-steel hand K-file step-back instrumentation techniques were not significantly different in their ability to reduce intracanal bacteria. All bacterial samples were significantly lower than the initial sample, regardless of file type. |

| **Supplementary table 2: Microbiological sampling of root canals** | | | | | | | | | |
| --- | --- | --- | --- | --- | --- | --- | --- | --- | --- |
| Author | Year | Sampling Technique | Sampling Collection Source | S1 | S2 | S3 | S4 | S5 | Sterility control sample (YES/NO) |
|  |  |  |  |  |  |  |  |  |  |
| Sarkees et al | 2025 | Paper points | Intracanal | Before CMP | After CMP | After final flush, with either;2 % IKI or with 5% IKI | NA | NA | NO |
| Nogales et al | 2025 | H-file & paper points | Intracanal | Before CMP | After CMP | After ultrasonic irrigation | NA | NA | YES |
| Kavalipurapu et al | 2024 | Paper points | Intracanal | Before CMP | After irrigation protocol | NA | NA | NA | NO |
| Shroff et al | 2024 | Paper points | Intracanal | Before CMP | After ICM | NA | NA | NA | NO |
| Sesar et al | 2024 | H-file & paper points | Intracanal | Before CMP | After CMP | After the final irrigation protocol | NA | NA | YES |
| Konadu et al | 2024 | Paper points. | Intracanal | Before CMP | After CMP and irrigation | NA | NA | NA | NO |
| Kesim et al | 2024 | Paper points | Intracanal | Before CMP | After CMP | After ICM | NA | NA | YES |
| Hepsenoglu et al. | 2024 | Paper points | Intracanal | Before CMP | After CMP | After final irrigation activation | NA | NA | YES |
| Fahim et al. | 2024 | Paper points | Intracanal | Before CMP | After CMP and disifection protocol | NA | NA | NA | NO |
| Eltantawi et al | 2024 | Paper points | Intracanal | Before CMP | After 7 days ICM | NA | NA | NA | NO |
| Barazy et al | 2024 | H- file & paper points | Intracanal | Before CMP | After disinfection protocols | NA | NA | NA | NO |
| Babeer et al | 2024 | H- file & paper points | Intracanal | Before CMP | After CMP | NA | NA | NA | NO |
| Alquria et al. | 2024 | Paper points | Intracanal | Before CMP | After CMP | NA | NA | NA | YES |
| Abdel-Hamid et al.. | 2024 | Paper points | Intracanal | Before CMP | After CMP | NA | NA | NA | NO |
| Wenzler et al. | 2023 | Paper points | Intracanal | Before CMP | After CMP | After additional rinsing with NaOCl | NA | NA | NO |
| Rodríguez et al | 2023 | Sterile Capillary Tip (0.035mm) connected to a 5 mL hypodermic syringe | Intracanal | Before CMP | After CMP | After ICM | NA | NA | NO |
| Rôças et al | 2023 | K-file & paper points | Intracanal | Before CMP | After CMP | After 7 days ICM with either; (CHG),(CHPG),(CHCHX) | NA | NA | YES |
| Rajamanickam et al | 2023 | Paper points | Intracanal | Before CMP | After CMP wth irrigation protocols | NA | NA | NA | NO |
| Sadhana Rai et al | 2023 | Paper points | Intracanal | Before CMP | After CMP | NA | NA | NA | NO |
| Laís Lima Pelozo et al | 2023 | K- file & paper points | Intracanal | After removal of the root filling | After diode laser or placebo irradiation | NA | NA | NA | NO |
| Revathi Palanisamy et al | 2023 | Paper points | Intracanal | Before CMP | After the final iriigation protocol | NA | NA | NA | YES |
| Nikhade et al | 2023 | Paper points | Intracanal | Before CMP | After CMP | After 7 days ICM | NA | NA | NO |
| Leonardo et al | 2023 | Paper points | Intracanal | Before CMP | After CMP | After the application of ICG , laser activation, and 10 mL saline solution | NA | NA | NO |
| Khandelwal et al. | 2023 | Paper points | Intracanal | Before CMP | After CMP and irrigation with either NaOCl gel or aqueous solution | NA | NA | NA | NO |
| Karataş et al | 2023 | Paper points | Intracanal | Before CMP | After CMP | After 15 days ICM | NA | NA | YES |
| Hepsenoglu et al | 2023 | Paper points | Intracanal | Before CMP | After CMP | After final irrigation activation | NA | NA | YES |
| Seyda Ersahan et al | 2022 | K- file & paper points | Intracanal | Before CMP | After CMP | After 7 days ICM | NA | NA | YES |
| Rakasevic Dragana et al | 2023 | Paper points | Intracanal | Before CMP | After CMP | After the application of diode laser and PDT | NA | NA | NO |
| Alves-Silva et al | 2023 | Paper points | Intracanal & from periapical tissue | Before CMP | After CMP | After PDT | NA | NA | YES |
| Toia et al | 2022 | Paper points | Intracanal | Before removal of the root filling | After retreatment protocol | After ICM in two visit group only | NA | NA | YES |
| Tandon et al. | 2022 | Paper points | Intracanal | After removal of the root filling | After retreatment protocol | After ICM | NA | NA | YES |
| Saber et al. | 2022 | Paper points | Intracanal | Before CMP | After CMP | NA | NA | NA | YES |
| Mishra et al | 2022 | Paper points | Intracanal | Before CMP | After CMP | NA | NA | NA | NO |
| Omer et al | 2022 | Paper points | Intracanal | Before CMP | After CMP | After 7 days ICM | NA | NA | YES |
| Gabrielli et al | 2022 | Paper points | Intracanal & abscess collection | Before CMP | After CMP | After 30 days ICM | NA | NA | YES |
| Fahim et al | 2022 | Paper points | Intracanal | After removal of the root filling | After retreatment protocol | After 7 days ICM | NA | NA | NO |
| V. Di Taranto et al | 2022 | Paper points | Intracanal | Before CMP | After CMP | After PDT and diode laser | After 7 days ICM | After the second application of diode laser or PDT | NO |
| Rodrigo Arruda-Vasconcelos et al | 2022 | Paper points | Intracanal | Before CMP | After CMP | After 30 days ICM | NA | NA | YES |
| Wenzler et al | 2021 | Paper points | Intracanal | After emergency treatment | After CMP | After rinsing with 3% NaOCl along with one of three adjuvant protocols | NA | NA | NO |
| Nasr et al. | 2021 | Paper points | Intracanal | Before CMP | After final flush of either; CNPs, CHX, CHX/ CNPs combination, 5.25% NaOCl | NA | NA | NA | NO |
| Moreira et al | 2021 | Paper points | Intracanal | Before CMP | After CMP in GP 1, after PDT in GP 2 | After 15 days ICM in GP1, after PDT in GP2 | NA | NA | NO |
| Dalaei Moghadam et al | 2021 | Paper points | Intracanal | Before CMP | After CMP | After 7 days ICM | NA | NA | NO |
| KARATAŞ et al | 2021 | K-file & paper points | Intracanal | Before CMP | After CMP | NA | NA | NA | YES |
| Espaladori et al | 2021 | Paper points | Intracanal | Before CMP | After CMP | After15 days ICM | NA | NA | NO |
| Ramesh Bharti et al | 2021 | Paper points | Intracanal | After removal of the root filling | After retreatment protocol | NA | NA | NA | NO |
| R. Arruda-Vasconcelos et al | 2021 | Paper points | Intracanal | Before CMP | After CMP | After 30 days ICM | NA | NA | YES |
| Yalgi et al | 2020 | Paper points | Intracanal | Before CMP | After irrigation with either; NaOCl or C. officinalis | NA | NA | NA | NO |
| Siddique et al. | 2020 | Paper points | Intracanal | Before CMP | After CMP | NA | NA | NA | YES |
| Siddique et al. | 2020 | Paper points | Intracanal | Before CMP | After CMP | NA | NA | NA | YES |
| Orozco et al. | 2020 | Paper points | Intracanal | Before CMP | After CMP and NaOCl irrigation | After EDTA irrigation | NA | NA | NO |
| Neves et al | 2020 | Paper points | Intracanal | Before CMP | After CMP | After 7-10 days ICM | NA | NA | NO |
| Mittal et al | 2020 | Paper points | Intracanal | Before CMP | After CMP | NA | NA | NA | NO |
| Louzada et al | 2020 | Paper points | Intracanal and Periodontal pockets | Before CMP | After CMP | After 30 days ICM | NA | NA | YES |
| Karatas et al | 2020 | Paper points | Intracanal | Before CMP | After CMP | After 7 days ICM | NA | NA | YES |
| Irina M. Horlenko et al | 2020 | Paper points | Intracanal | Before CMP | After the second application of the root canal irrigants in the 2nd visit | NA | NA | NA | NO |
| Alexandre P. L. Carvalho et al | 2020 | H-file & paper points | Intracanal | Before CMP | After an initial flush with NaOCl without activation | **S3a**: After XPF instrument. **S3b:** after ultrsonic activation | After 14 days ICM | After final irrigation in the 2nd visit | YES |
| Barbosa-Ribeiro et al | 2020 | Paper points | Intracanal | After removal of the root filling | After retreatment protocol | After 30 days of Ca(OH)2 ICM | NA | NA | YES |
| Ballal et al | 2020 | Paper points | Intracanal | Before CMP | After CMP | After irrigant activation | NA | NA | YES |
| Aveiro et al. | 2020 | Paper points | Intracanal | Before CMP | After CMP and activation | NA | NA | NA | YES |
| Amaral et al. | 2020 | Paper points | Intracanal | Before CMP | After CMP | After XP-F | NA | NA | YES |
| Zorita-García et al | 2019 | Paper points | Intracanal | Before CMP | After CMP | After PDT | NA | NA | NO |
| Savitha et al | 2019 | H-file & paper points | Intracanal | After removal of the root filling | After retreatment protocol | After 7 days ICM | NA | NA | NO |
| Dias MACHADO et al | 2019 | Paper points | Intracanal | Before CMP | After CMP | NA | NA | NA | YES |
| Thais M. Duque et al | 2018 | Paper points | Intracanal & from peridontal pockets | Before CMP | After 30 days of ICM | NA | NA | NA | YES |
| Abdul Qadir Khan Dall et al | 2019 | Paper points | Intracanal | After CMP | After ICM | NA | NA | NA | NO |
| Barbosa-Ribeiro et al. | 2019 | Paper points | Intracanal | Before CMP | After 30 days of Ca(OH)2 ICM | NA | NA | NA | YES |
| Ballal et al | 2019 | Paper points | Intracanal | Before CMP | After CMP | After 7 days | NA | NA | NO |
| Sonarkar et al. | 2018 | Paper points | Intracanal | Before CMP | After CMP | After 7 days ICM and another application of either; PAD, diode laser, 5% NaOCl or normal saline | NA | NA | NO |
| Pourhajibagher et al. | 2018 | Paper points | Intracanal | Before BO-PAD combination | After BO-PAD combination | NA | NA | NA | YES |
| Nakamura et al | 2017 | Paper points | Intracanal | Before CMP | After CMP | After irrigation protocol | After ICM | NA | YES |
| Martinho et al | 2017 | Paper points | Intracanal | Before CMP | After CMP | After ICM | NA | NA | YES |
| Khedmat et al | 2018 | Paper points | Intracanal | Before CMP | After CMP | NA | NA | NA | NO |
| E. Iriboz et al | 2018 | Paper points | Intracanal | Before CMP | After CMP and 7 days ICM | NA | NA | NA | NO |
| Rachel Garcia de Miranda and AnaPaulaVieira Colomb | 2018 | Paper points | Intracanal | Before CMP | After CMP and final irrigation in the control group, and after CMP, final irrigation and PDT in the PDT group | After ICM and final irrigation | NA | NA | NO |
| Caroline C. da Silva et al | 2018 | Paper points | Intracanal | Before CMP | After CMP | After PDT for test group only | Before obturation | NA | YES |
| Arruda et al | 2018 | Paper points | Intracanal | Before CMP | After CMP | After ICM | NA | NA | YES |
| Rodrigues et al | 2017 | Paper points | Intracanal | Before CMP | After using the first instrument of the TFA system | After using the third instrument of the TFA system | After final irrigation with 1% NaOCl in the saline group only | NA | YES |
| Rabello et al. | 2017 | Paper points | Intracanal | Before CMP | After CMP | After PDT in GP 1, after 14-days ICM in GP 2 | After PDT in GP 2 only | NA | YES |
| Kist et al. | 2017 | Paper points | Intracanal | Before CMP | After CMP | After 7 days ICM | NA | NA | YES |
| Cavalli et al | 2017 | Paper points | Intracanal | Before CMP | After CMP | NA | NA | NA | NO |
| Asnaashari et al | 2017 | Paper points | Intracanal | After removal of the root filling and CMP | After disinfection therapy either with; PDT or Ca(OH)2 ICM | NA | NA | NA | NO |
| Zandi et al | 2016 | Paper points | Intracanal | Before CMP | After CMP using either NaOCl or CHX irrigation | After Ca(OH)2 ICM | NA | NA | YES |
| Isabela N. R^oc et al | 2016 | Paper points | Intracanal | Before CMP | After CMP | NA | NA | NA | YES |
| Cristina Rico-Romano et al | 2016 | Paper points | Intracanal | Before CMP | After CMP | NA | NA | NA | NO |
| Monica A.S. Neves et al | 2016 | Paper points | Intracanal | Before CMP | After CMP | NA | NA | NA | YES |
| Ghoneim et al. | 2016 | Paper points | Intracanal | Before CMP | After CMP | NA | NA | NA | NO |
| Donyavi et al | 2016 | Paper points | Intracanal | Before CMP | After CMP ;In gp 1: After irrigation with NaOCl, while in GP 2 after ICM | NA | NA | NA | NO |
| Ribeiro et al. | 2016 | Paper points | Intracanal | Before CMP | After CMP | After 30 days of ICM | NA | NA | YES |
| Asnaashari et al | 2016 | Paper points | Intracanal | Before CMP | After CMP | NA | NA | NA | NO |
| Zeledón et al | 2015 | Paper points | Intracanal | Before CMP | After initial disinfectation with 5% NaOCL and and neutralization with sodium thiosulfate | After CMP | After ICM | NA | YES |
| Rodrigues et al | 2015 | Paper points | Intracanal | Before CMP | After CMP with either SAF or TFA. S2b was taken in TFA group after PUI | NA | NA | NA | YES |
| Provenzano et al | 2015 | Sterile disposable syringe | Intracanal | Before CMP | After CMP with either NaOCl or CHX | After ICM | NA | NA | YES |
| Frederico C. Martinho et al | 2015 | Paper points | Intracanal | Before CMP | After CMP | After 14 days ICM | NA | NA | YES |
| Frederico C. Martinho & Lilian F. Freitas & Gustavo G. Nascimento & Aleteia M Fernandes & Fabio R. M. Leite & Ana P. M. Gomes & Izabel C G Camões | 2015 | Paper points | Intracanal | Before removal of the root filling | After retreatment protocol | NA | NA | NA | NO |
| Marinho et al | 2015 | Paper points | Intracanal | Before CMP | After CMP | After irrigation with 17% EDTA | After 30 days ICM | Before obturation | NO |
| Ferreira et al. | 2015 | Paper points | Intracanal | Before CMP | After CMP | After 14 days Ca(OH)2 ICM | NA | NA | YES |
| Adl et al. | 2015 | Paper points | Intracanal | Before CMP | After CMP | After 7 days Ca(OH)2 ICM | NA | NA | YES |
| Telesa et al | 2014 | Paper points | Intracanal | Before CMP | After CMP | After 14 daysICM | NA | NA | YES |
| Stojanović et al | 2014 | Paper points | Intracanal | Before CMP | After CMP | After 15day ICM | NA | NA | YES |
| Isabela N. R^oc et al | 2014 | Paper points | Intracanal | Before CMP | After CMP | NA | NA | NA | YES |
| M. A. S. Neves et al | 2014 | Paper points | Intracanal | Before CMP | After CMP | NA | NA | NA | YES |
| Shailendra Mashalkar et al | 2014 | Paper points | Intracanal | Before CMP | After diode laser irradiation in GP 1, and after CMP with NaOCl and H2O2 in GP 2 | NA | NA | NA | NO |
| Martinho et al | 2014 | Paper points | Intracanal | Before CMP | After CMP | NA | NA | NA | YES |
| Juric et al. | 2014 | Paper points | Intracanal | After removal of the root filling | After retreatment protocol | After PDT | NA | NA | NO |
| Xavier et al. | 2013 | Paper points | Intracanal | Before CMP | After CMP in GP 1 , After Ca(OH)2 ICM in GP 2 | NA | NA | NA | YES |
| Rôças et al. | 2013 | Paper points | intracanal | Before CMP | After CMP | NA | NA | NA | YES |
| Provenzano et al | 2013 | Sterile disposable syringe | Intracanal and apical abscess | Before CMP, abscess sample: collected at the time of clinical presentation | After CMP | NA | NA | NA | NO |
| Paiva et al | 2013 | Paper points | Intracanal | Before CMP | After CMP | After ultrasoic activation with NaOCl | NA | NA | YES |
| Paiva et al | 2013 | Paper points | Intracanal | Before CMP | After CMP with NaOCl | After final rinse with CHX | After 7 days Ca(OH)2/CHX ICM | NA | YES |
| Kristina Halbauer et al | 2013 | Paper points | Intracanal | After CMP | After the ozone treatment | NA | NA | NA | NO |
| Endo et al | 2013 | Paper points | Intracanal | After removal root canal filling | After CMP | After 7-14 days ICM | NA | NA | YES |
| Cohenca et al | 2013 | Paper points | Intracanal | Before CMP | After CMP | After 7 days ICM | NA | NA | YES |
| Endo et al. | 2012 | Paper points | Intracanal | After removal of the root filling | After retreatment protocol | NA | NA | NA | YES |
| Rôças et al | 2011 | Paper points | Intracanal | Before CMP | After CMP | NA | NA | NA | YES |
| Rôças et al | 2011 | Paper points | Intracanal | Before CMP | After CMP | After ICM | NA | NA | YES |
| Martinho et al | 2010 | Paper points | Intracanal | Before CMP | After CMP | NA | NA | NA | YES |
| Abbas Abbaszadegan et al | 2010 | Paper points | Intracanal | Before CMP | After CMP either with; 2.5%NaOCl or 2% IKI | NA | NA | NA | NO |
| Gevik Malkhassian et al | 2009 | Paper points | Intracanal | Before CMP | After CMP using NaOCl | After final rinse either with MTAD or saline | After 2%CHX gel ICM in 2nd visit | After final rinse with NaOCl in 2nd visit | YES |
| Bebek et al | 2009 | Paper points | Intracanal | Before CMP | After 48h, at 2nd visit | NA | NA | NA | NO |
| Vianna et al. | 2008 | Paper points | Intracanal | Before CMP | After CMP | After 7 days ICM | NA | NA | YES |
| Martinho et al. | 2008 | Paper points. | Intracanal | Before CMP | After CMP | NA | NA | NA | YES |
| Garcez et al | 2008 | Paper points | Intracanal | Before CMP | After CMP | After the first PDT. | After Ca(OH)2 ICM | After second session of PDT | NO |
| Garcez et al | 2008 | Paper points | Intracanal | Before CMP | After CMP | After PDT in group 2 only. | NA | NA | NO |
| Blome B et al | 2008 | Paper points | Intracanal | Before CMP | After CMP | After 14 days Ca(OH)2 ICM | NA | NA | YES |
| Wang et al | 2007 | K-file & paper points | Intracanal | Before CMP | After CMP | After 14 days ICM | NA | NA | YES |
| Vianna ME et al | 2007 | Paper points | Intracanal | Before CMP | After CMP | After ICM | NA | NA | YES |
| José F. Siqueira et al | 2007 | Paper points | Intracanal | Before CMP | After CMP either with; CHX, NaOCl | NA | NA | NA | YES |
| Siqueira et al | 2007 | Paper points | Intracanal | Before CMP | After CMP | After 7 days ICM | NA | NA | YES |
| Karen et al. | 2007 | Paper points | Intracanal | Before CMP | After CMP | After 7 days ICM | NA | NA | YES |
| Siqueira Jr et al. | 2007 | Paper points | Intracanal | Before CMP | After CMP without EDTA irrigation | After 7days Ca(OH)2 ICM + EDTA irrigation | NA | NA | NO |
| Schirrmeister et al. | 2007 | Paper points | Intracanal | After removal of the root filling | After CMP (NaOCl and EDTA irrigation) | After CHX irrgation | After Ca(OH)2 ICM | NA | NO |
| Sakamoto et al | 2007 | Paper points | Intracanal | Before CMP | After CMP | After 7 days ICM | NA | NA | YES |
| Paquette et al | 2007 | Sterile endodontic syringe and a 27-gauge needle | Intracanal | Before CMP | After CMP | After 7-15 days ICM | After final irrigation at 2nd visit | NA | YES |
| Manzur et al | 2007 | Paper points | Intracanal | Before CMP | After CMP | After 7 days ICM | NA | NA | YES |
| Carver et al | 2007 | K- file & paper points. | Intracanal | Before CMP | After CMP | After ultrasonic irrigation | NA | NA | NO |
| M.E. Vianna et al | 2006 | Paper points | Intracanal | Before CMP | After CMP either with; CHX, NaOCl | NA | NA | NA | YES |
| Janir Alves SOARES et al | 2006 | Paper points | Intracanal | Before CMP | After CMP | NA | NA | NA | NO |
| Chu et al. | 2006 | Paper points | Intracanal | Before CMP | After 7 days ICM | NA | NA | NA | YES |
| McGurkin-Smith et al | 2005 | Paper points | Intracanal | Before CMP | After CMP | After >1 week of Ca(OH)2 ICM . | NA | NA | NO |
| Ferrari et al. | 2005 | Paper points | Intracanal | Before CMP | After CMP | At the 2nd visit ( after 7 days) | After PRP at the 3rd visit (after 7 days) | NA | NO |
| Souza et al. | 2005 | Paper points | Intracanal | Before CMP | After 14 days Ca(OH)2 ICM | NA | NA | NA | NO |
| Kvist et al | 2004 | Charcoal points | Intracanal | Before CMP | After CMP | After either 5% IKI in the One-Visit GP, or after 7 days Ca(OH)2 ICM in the Two-Visit GP | NA | NA | YES |
| Ercan et al | 2004 | Paper points | Intracanal | Before CMP | After CMP | After 48-hr Post-CMP | NA | NA | NO |
| Peters et al | 2002 | Paper points | Intracanal | Before CMP in both groups | After CMP in both groups | After 4 weeks Ca(OH)2 ICM in 2nd visit only | After sodium thiosulphate flush in 2nd visit only | NA | YES |
| TATJANADOSTÁLOVÁ et al | 2002 | Not mentioned | Intracanal | Before CMP | After either ; Ca(OH)2 ICM / laser irradiation. | NA | NA | NA | NO |
| Peciuliene et al | 2001 | Paper points | Intracanal | Before CMP | After CMP | After IKI irrigation in group B only | NA | NA | NO |
| George B. Shuping et al. | 2000 | Paper points | Intracanal | Before CMP | After initial CMP | During CMP | After final CMP | After ICM | NO |
| Dalton et al | 1998 | Paper points | Intracanal | Before CMP | During CMP;after final frist file | During CMP;after final second file | After CMP; after the third final file | NA | NO |

**Supplementary table 3: reasons for exclusion at full text screening stage**

| Author | Year | Reason of exclusion |
| --- | --- | --- |
| Alves et al. | 2024 | Study Protocol |
| Zahran et al. | 2021 | Not reporting antibacterial effect of root canal disinfection |
| Motiwala et al. | 2021 | Protocol |
| Zandi et al. | 2019 | Same population previously examined |
| Asnaashari et al. | 2016 | pilot study |
| Garcez et al. | 2015 | Not reporting non-surgical RCT |
| Teles et al. | 2014 | same population previously examined |
| Mitić et al. | 2013 | Not in English |
| Lima et al. | 2013 | Not reporting permenant teeth |
| Paiva et al. | 2012 | same population previously examined |
| Garcez et al. | 2010 | Study report ( pilot study) |
| Jiang et al. | 2009 | Not in English |
| Reit et al. | 1999 | Not reporting the antibacterial effect of root canal disinfection |
| Orstavik et al. | 1991 | Pilot study |

**References**

1. Sarkees M, Alafif H, Alsalameh SA, Achour H. Efficacy of Two Different Concentrations of Iodine-potassium Iodide Solution in Endodontic Retreatment: A Randomised Double-blinded Clinical Trial. European Endodontic Journal. 2025;10(1):27.

2. Nogales CG, Cazares RXR, Nardello LCL, Mayer MPA, Gavini G, Zehnder M, et al. Evaluating the impact of ultrasonic irrigation on bacterial levels and activity following chemomechanical procedures. Journal of Endodontics. 2025;51(2):118-23.

3. Teja KV, Ramesh S, Srivastava KC, Ehsan A, Choudhari S, Shrivastava D. Comparative Evaluation of Novel Automated Irrigation Devices with Side-Vented Needle Irrigation on Bacterial CFU Counts in Nonvital Teeth. Journal of Pharmacy and Bioallied Sciences. 2024;16(Suppl 5):S4811-S5.

4. Shroff M, Brave D, Rathore VPS, Sharma V, Mehta J, Thakkar SJ. Antimicrobial Efficacy of Simvastatin and Double Antibiotic Paste as Intracanal Medicaments: A Randomised Clinical Study. Advances in Human Biology. 2024;14(2):158-62.

5. Sesar A, Budimir A, Anić I, Petričević GK, Bago I. Antibacterial Efficacy of Rotary and Reciprocating Instrumentation Techniques. Acta stomatologica Croatica. 2024;58(3):245.

6. Konadu AB, Ampofo PC, Akyeh ML, Hewlett SA, Osei-Tutu K, Nyako EA. Comparative evaluation of selected concentrations of sodium hypochlorite on the outcome of endodontic therapy among Ghanaians. Plos one. 2024;19(7):e0306693.

7. Kesim B, Tezcan Ülger S, Aslan G, Üstün Y, Avcı AT, Küçük MÖ. Effects of Sequential Antimicrobial Phases on Root Canal Microbiome Dynamics in Two-Visit Treatment of Primary Apical Periodontitis: A Longitudinal Experimental Study. Life. 2024;14(12):1696.

8. Hepsenoglu YE, Ersahan S, Erkan E, Gundogar M, Ozcelik F. Is SWEEPS better than PUI in reducing intracanal bacteria and inflammation in cases of apical periodontitis? Lasers in Medical Science. 2024;39(1):182.

9. Fahim SZ, Ghali RM, Hashem AA, Farid MM. The efficacy of 2780 nm er, Cr; YSGG and 940 nm Diode Laser in root canal disinfection: a randomized clinical trial. Clinical Oral Investigations. 2024;28(3):175.

10. Eltantawi AR, Abdel-Razik GM, Elhawary YM, Badr AE. Efficacy of Glycyrrhizin as an Intracanal Medicament on Bacterial Load Reduction in Primary Infected Root Canals: A Randomized Clinical Trial. The Journal of Contemporary Dental Practice. 2024;25(6):540-6.

11. Barazy R, Alafif H, Achour H, Al-Aloul A, Alsayed Tolibah Y. Can antimicrobial photodynamic therapy serve as an effective adjunct protocol for disinfecting the necrotic root canal system? A randomized controlled study. BDJ open. 2024;10(1):53.

12. Babeer A, Liu Y, Ren Z, Xiang Z, Oh MJ, Pandey NK, et al. Ferumoxytol nanozymes effectively target chronic biofilm infections in apical periodontitis. The Journal of Clinical Investigation. 2025;135(3).

13. Alquria TA, Acharya A, Tordik P, Griffin I, Martinho FC. Impact of root canal disinfection on the bacteriome present in primary endodontic infection: A next generation sequencing study. International Endodontic Journal. 2024;57(8):1124-35.

14. Abdel-Hamid LZI, El Boghdadi RM, Gawdat SI, Salem ST, Soliman NS. Assessment of Dual Rinse Combined with Sodium Hypochlorite Irrigating Solution on Post-Instrumentation Pain and Bacterial Load Reduction: A Randomized Clinical Trial. Brazilian Dental Science. 2024;27(4).

15. Wenzler J-S, Falk W, Frankenberger R, Braun A. Temporary Root Canal Obturation with a Calcium Hydroxide-Based Dressing: A Randomized Controlled Clinical Trial. Antibiotics. 2023;12(12):1663.

16. Oliva Rodríguez R, Rangel Galván GY, González Amaro AM, Gutiérrez Cantú FJ, Muñoz Ruiz AI, García Cortés JO, et al. Correlation between bacterial type/bacterial quantity and bone loss detected by cone beam computed tomography (CBCT) in primary endodontic infections. Investigación Clínica. 2023;64(1):5-14.

17. Rôças IN, Provenzano JC, Neves MS, Alves FR, Gonçalves LS, Siqueira Jr JF. Effects of calcium hydroxide paste in different vehicles on bacterial reduction during treatment of teeth with apical periodontitis. Journal of Endodontics. 2023;49(1):55-61.

18. Rajamanickam K, Raghu S, Priyadharsini JV, Antony DP, Sureshbabu NM, Priyadharsini VJ, et al. Comparative evaluation of bacterial reduction by laser-activated irrigation technique (LAI) with conventional needle irrigation (CNI) in single-rooted teeth with pulpal necrosis: a single-blinded randomized controlled trial. Cureus. 2023;15(12).

19. Rai S, Sundaramoorthy N, Mahalaxmi S, Purushothaman PV. Bioburden assessment of necrotic teeth disinfected with sodium hypochlorite, diode laser, and photodynamic therapy using flow cytometry—a randomized double-blinded clinical trial. Lasers in Medical Science. 2023;38(1):216.

20. Pelozo LL, Silva-Neto RD, Salvador SL, Sousa-Neto MD, Souza-Gabriel AE. Adjuvant therapy with a 980-nm diode laser in root canal retreatment: randomized clinical trial with 1-year follow-up. Lasers in Medical Science. 2023;38(1):77.

21. Palanisamy R, Anirudhan S, Roja RJS, Koshy M. Comparison of ultrasonic versus side-vented needle irrigation for reductions in bacterial growth and postoperative pain: A randomized controlled trial. Journal of Conservative Dentistry and Endodontics. 2023;26(6):616-20.

22. Nikhade P, Agrawal P, Mahapatra J, Suryawanshi T, Bhopatkar J, Umate L, et al. Efficacy of triple antibiotic paste and bromelain paste as intracanal medicament against Enterococcus faecalis: an in-vivo study. Cureus. 2023;15(11).

23. de Toledo Leonardo R, Puente CG, Berbert FLCV, Faria G, Nishiyama CK, Orosco FA, et al. Clinical study of antimicrobial efficacy of laser ablation therapy with indocyanine green in root canal treatment. Journal of Endodontics. 2023;49(8):990-4.

24. Kotecha N, Shah NC, Doshi RJ, Kishan KV, Luke AM, Shetty KP, et al. Microbiological effectiveness of sodium hypochlorite gel and aqueous solution when implemented for root canal disinfection in multirooted teeth: A randomized clinical study. Journal of Functional Biomaterials. 2023;14(5):240.

25. Karataş E, Kırmızıbekmez Ö, Baltacı MÖ, Adıgüzel A. Effect of triple antibiotic paste, double antibiotic paste, and calcium hydroxide on antibiotic resistance of tet repressor proteins, tetracycline resistance gene W, and tetracycline resistance gene Q: a randomized controlled clinical trial. Journal of Dentistry Indonesia. 2023;30(3):180-9.

26. Hepsenoglu YE, Ersahan S. Is XP‐endo Finisher a better treatment option for its efficacy against intracanal bacteria for post‐treatment apical periodontitis cases than E ndo A ctivator? Australian Endodontic Journal. 2023;49:399-412.

27. Ersahan S, Hepsenoglu YE. Microbial analysis of endodontic infections in teeth with post‐treatment apical periodontitis before and after medication. Australian Endodontic Journal. 2023;49(1):75-86.

28. Dragana R, Jelena M, Jovan M, Biljana N, Dejan M. Antibacterial efficiency of adjuvant photodynamic therapy and high-power diode laser in the treatment of young permanent teeth with chronic periapical periodontitis. A prospective clinical study. Photodiagnosis and Photodynamic Therapy. 2023;41:103129.

29. Alves-Silva EG, Arruda-Vasconcelos R, Louzada LM, de-Jesus-Soares A, Ferraz CCR, Almeida JFA, et al. Effect of antimicrobial photodynamic therapy on the reduction of bacteria and virulence factors in teeth with primary endodontic infection. Photodiagnosis and photodynamic therapy. 2023;41:103292.

30. Toia CC, Khoury RD, Corazza BJM, Orozco EIF, Valera MC. Effectiveness of 1-visit and 2-visit endodontic retreatment of teeth with persistent/secondary endodontic infection: a randomized clinical trial with 18 months of follow-up. Journal of Endodontics. 2022;48(1):4-14.

31. Tandon VKBJ, Taneja S. Evaluation of bacterial reduction at various stages of endodontic retreatment after use of different disinfection regimens: an in vivo study. European Endodontic Journal. 2022;7(3):210.

32. Saber SM, Alfadag AMA, Nawar NN, Plotino G, Hassanien EES. Instrumentation kinematics does not affect bacterial reduction, post‐operative pain, and flare‐ups: A randomized clinical trial. International Endodontic Journal. 2022;55(5):405-15.

33. Mishra A, Koul M, Abdullah A, Khan N, Dhawan P, Bhat A. Comparative evaluation of antimicrobial efficacy of diode laser (Continuous Mode), diode laser (Pulse Mode), and 5.25% of Sodium hypochlorite in disinfection of root canal: A short study. International Journal of Clinical Pediatric Dentistry. 2022;15(5):579.

34. Mahfouz Omer SM, Mohamed DAA, Ali Abdel Latif RM. Comparative evaluation of the antibacterial effect of Allium sativum, calcium hydroxide and their combination as intracanal medicaments in infected mature anterior teeth: A randomized clinical trial. International Endodontic Journal. 2022;55(10):1010-25.

35. Gabrielli ES, Lima AR, Francisco PA, Herrera DR, de-Jesus-Soares A, Ferraz CC, et al. Comparative analysis of bacterial content, levels of lipopolysaccharides and lipoteichoic acid in symptomatic and asymptomatic endodontic infections at different stages of endodontic treatment. Clinical Oral Investigations. 2022:1-16.

36. Fahim MM, Saber SEM, Elkhatib WF, Nagy MM, Schafer E. The antibacterial effect and the incidence of post-operative pain after the application of nano-based intracanal medications during endodontic retreatment: a randomized controlled clinical trial. Clinical oral investigations. 2022:1-9.

37. Di Taranto V, Libonati A, Montemurro E, Gallusi G, Campanella V. Antimicrobial effects of photodynamic and high-power laser endodontic therapy on patients with necrotic pulp and periapical lesion. JOURNAL OF BIOLOGICAL REGULATORS & HOMEOSTATIC AGENTS. 2022;36:41-8.

38. Arruda-Vasconcelos R, Barbosa-Ribeiro M, Louzada LM, Lemos BI, de-Jesus-Soares A, Ferraz CC, et al. Efficacy of 6% sodium hypochlorite on infectious content of teeth with symptomatic irreversible pulpitis. Journal of Endodontics. 2022;48(2):179-89.

39. Wenzler J-S, Falk W, Frankenberger R, Braun A. Impact of adjunctive laser irradiation on the bacterial load of dental root canals: A randomized controlled clinical trial. Antibiotics. 2021;10(12):1557.

40. Nasr M, Diab A, Roshdy N, Hussein A. Assessment of antimicrobial efficacy of nano chitosan, chlorhexidine, chlorhexidine/nano chitosan combination versus sodium hypochlorite irrigation in patients with necrotic mandibular premolars: a randomized clinical trial. Open Access Macedonian Journal of Medical Sciences. 2021;9(D):235-42.

41. de Azevedo Moreira S, Nunes JB, Colombo FA, Fonseca NdSM, Viola NV. Radiographic and antimicrobial evaluation of enterococcus Faecalis and Actinomyces Israelii micro-organisms after photodynamic therapy (aPDT). Photodiagnosis and Photodynamic Therapy. 2021;35:102433.

42. Moghadam MD, Saberi EA, Molashahi NF, Ebrahimi HS. Comparative efficacy of depotphoresis and diode laser for reduction of microbial load and postoperative pain, and healing of periapical lesions: a randomized clinical trial. Giornale Italiano di Endodonzia. 2021;35(2).

43. Karataş E, Ayaz N, UlukÖylÜ E, Baltaci MÖ, Adigüzel A. Effect of final irrigation with sodium hypochlorite at different temperatures on postoperative pain level and antibacterial activity: a randomized controlled clinical study. Journal of Applied Oral Science. 2021;29:e20200502.

44. Espaladori MC, Diniz JMB, de Brito LCN, Tavares WLF, Kawai T, Vieira LQ, et al. Selenium intracanal dressing: Effects on the periapical immune response. Clinical Oral Investigations. 2021;25:2951-8.

45. Bharti R, Tikku AP, Chandra A, Gupta P. Antimicrobial effectiveness of photodynamic therapy, 5% sodium hypochlorite and 2% chlorhexidine gluconate in root canal treated teeth: a clinical study. Journal of Advanced Oral Research. 2021;12(2):193-9.

46. Arruda-Vasconcelos R, Louzada LM, Feres M, Tomson PL, Cooper PR, Gomes BPFA. Investigation of microbial profile, levels of endotoxin and lipoteichoic acid in teeth with symptomatic irreversible pulpitis: a clinical study. International Endodontic Journal. 2021;54(1):46-60.

47. Yalgi VS, Bhat KG. Compare and Evaluate the Antibacterial Efficacy of Sodium Hypochlorite and Calendula Officinalis against Streptococcus Mutans as a Root Canal Irrigating Solution: An: In vivo: Study. Journal of International Oral Health. 2020;12(1):74-9.

48. Siddique R, Ranjan M, Jose J, Srivastav A, Rajakeerthi R, Kamath A. Clinical Quantitative Antibacterial Potency of Garlic-Lemon Against Sodium Hypochlorite in Infected Root Canals: A Double-blinded, Randomized, Controlled Clinical Trial. Journal of International Society of Preventive and Community Dentistry. 2020;10(6):771-8.

49. Siddique R, Nivedhitha MS, Ranjan M, Jacob B, Solete P. Comparison of antibacterial effectiveness of three rotary file system with different geometry in infected root canals before and after instrumentation–a double-blinded randomized controlled clinical trial. BDJ Open. 2020;6(1):8.

50. Orozco EIF, Toia CC, Cavalli D, Khoury RD, Cardoso F, Bresciani E, et al. Effect of passive ultrasonic activation on microorganisms in primary root canal infection: a randomized clinical trial. J Appl Oral Sci. 2020;28:e20190100.

51. Neves MAS, Provenzano JC, Fonseca SCL, Rodrigues RCV, Gonçalves LS, Siqueira JF, Jr., et al. Disinfection and outcome of root canal treatment using single-file or multifile systems and Ca(OH)2 medication. Braz Dent J. 2020;31(5):493-8.

52. Mittal R, Tandan M, Jain V. A randomized clinical trial of antimicrobial efficacy of photoactivated disinfection, conventional endodontic irrigation and their combination in primary endodontic infections. International Journal of Oral Health Sciences. 2020;10(2):94-101.

53. Louzada LM, Arruda-Vasconcelos R, Duque TM, Casarin RCV, Feres M, Gomes BPFA. Clinical Investigation of Microbial Profile and Levels of Endotoxins and Lipoteichoic Acid at Different Phases of the Endodontic Treatment in Teeth with Vital Pulp and Associated Periodontal Disease. Journal of Endodontics. 2020;46(6):736-47.

54. Karataş E, Baltacı MÖ, Uluköylü E, Adıgüzel A. Antibacterial effectiveness of calcium hydroxide alone or in combination with Ibuprofen and Ciprofloxacin in teeth with asymptomatic apical periodontitis: a randomized controlled clinical study. International Endodontic Journal. 2020;53(6):742-53.

55. Horlenko IM, Gadzhula NG, Cherepakha OL, Kurdysh LF, Pylypiuk OY. Clinical and microbiological assessment of root canal decontamination in chronic apical periodontitis using the ultrasound. Wiad Lek. 2020;73(6):1119-23.

56. Carvalho APL, Nardello LCL, Fernandes FS, Bruno FP, Paz LR, Iglecias EF, et al. Effects of Contemporary Irrigant Activation Schemes and Subsequent Placement of an Interim Dressing on Bacterial Presence and Activity in Root Canals Associated with Asymptomatic Apical Periodontitis. Journal of Clinical Medicine. 2020;9(3):854.

57. Barbosa-Ribeiro M, Arruda-Vasconcelos R, Mendes Louzada L, Rodrigues Lima A, Marciano M, Affonso de Almeida JF, et al. Microbiological Investigation in Teeth with Persistent/Secondary Endodontic Infection in Different Stages of Root Canal Retreatment. Eur Endod J. 2020;5(3):219-25.

58. Ballal NV, Gandhi P, Shenoy PA, Dummer PMH. Evaluation of various irrigation activation systems to eliminate bacteria from the root canal system: A randomized controlled single blinded trial. J Dent. 2020;99:103412.

59. Aveiro E, Chiarelli-Neto VM, de-Jesus-Soares A, Zaia AA, Ferraz CCR, Almeida JFA, et al. Efficacy of reciprocating and ultrasonic activation of 6% sodium hypochlorite in the reduction of microbial content and virulence factors in teeth with primary endodontic infection. Int Endod J. 2020;53(5):604-18.

60. Amaral RR, Guimarães Oliveira AG, Braga T, Reher P, de Macêdo Farias L, Magalhães PP, et al. Quantitative Assessment of the Efficacy of Two Different Single-file Systems in Reducing the Bacterial load in Oval-Shaped Canals: A Clinical Study. J Endod. 2020;46(9):1228-34.

61. Zorita-García M, Alonso-Ezpeleta L, Cobo M, Del Campo R, Rico-Romano C, Mena-Álvarez J, et al. Photodynamic therapy in endodontic root canal treatment significantly increases bacterial clearance, preventing apical periodontitis. Quintessence Int. 2019;50(10):782-9.

62. Savitha A, SriRekha A, Vijay R, Ashwija, Champa C, Jaykumar T. An in vivo comparative evaluation of antimicrobial efficacy of chitosan, chlorhexidine gluconate gel and their combination as an intracanal medicament against Enterococcus faecalis in failed endodontic cases using real time polymerase chain reaction (qPCR). Saudi Dent J. 2019;31(3):360-6.

63. Machado CAD, Souza ACA, Loureiro C, Martinho FC, Cintra L, Dezan Junior E, et al. Comparison of two rotary systems in bacteria/lps removal from endodontic infections: randomized clinical trial. Braz Oral Res. 2019;33:e039.

64. Duque TM, Prado M, Herrera DR, Gomes B. Periodontal and endodontic infectious/inflammatory profile in primary periodontal lesions with secondary endodontic involvement after a calcium hydroxide-based intracanal medication. Clin Oral Investig. 2019;23(1):53-63.

65. Dall AQ, Bibi B, Babikar A, Qureshi S. Reduction of Pain and Bacterial Presence by using Double and Triple Antibiotic Pastes in Symptomatic Apical Periodontitis. 2019.

66. Barbosa-Ribeiro M, Arruda-Vasconcelos R, de-Jesus-Soares A, Zaia AA, Ferraz CCR, de Almeida JFA, et al. Effectiveness of calcium hydroxide-based intracanal medication on infectious/inflammatory contents in teeth with post-treatment apical periodontitis. Clin Oral Investig. 2019;23(6):2759-66.

67. Ballal NV, Gandhi P, Shenoy PA, Shenoy Belle V, Bhat V, Rechenberg DK, et al. Safety assessment of an etidronate in a sodium hypochlorite solution: randomized double-blind trial. Int Endod J. 2019;52(9):1274-82.

68. Sonarkar SS, Singh S, Podar R, Kulkarni G, Purba R. An in vivo comparison of the antibacterial efficacy of photoactivated disinfection, diode laser, and 5% sodium hypochlorite in root canal disinfection. J Conserv Dent. 2018;21(2):205-9.

69. Pourhajibagher M, Bahador A. An in vivo evaluation of microbial diversity before and after the photo-activated disinfection in primary endodontic infections: Traditional phenotypic and molecular approaches. Photodiagnosis Photodyn Ther. 2018;22:19-25.

70. Nakamura VC, Pinheiro ET, Prado LC, Silveira AC, Carvalho APL, Mayer MPA, et al. Effect of ultrasonic activation on the reduction of bacteria and endotoxins in root canals: a randomized clinical trial. Int Endod J. 2018;51 Suppl 1:e12-e22.

71. Martinho FC, Gomes CC, Nascimento GG, Gomes APM, Leite FRM. Clinical comparison of the effectiveness of 7- and 14-day intracanal medications in root canal disinfection and inflammatory cytokines. Clin Oral Investig. 2018;22(1):523-30.

72. Khedmat S, Fakhari N, Emaneini M, Beigverdi R. Comparison of Antibacterial Effect of Four Irrigation Solutions in Primary Root Canal Infections: A Clinical Study. Iran Endod J. 2018;13(4):534-9.

73. İriboz E, Arıcan Öztürk B, Kolukırık M, Karacan I, Sazak Öveçoğlu H. Detection of the unknown components of the oral microflora of teeth with periapical radiolucencies in a Turkish population using next-generation sequencing techniques. Int Endod J. 2018;51(12):1349-57.

74. de Miranda RG, Colombo APV. Clinical and microbiological effectiveness of photodynamic therapy on primary endodontic infections: a 6-month randomized clinical trial. Clin Oral Investig. 2018;22(4):1751-61.

75. da Silva CC, Chaves Júnior SP, Pereira GLD, Fontes KBFdC, Antunes LAA, Póvoa HCC, et al. Antimicrobial Photodynamic Therapy Associated with Conventional Endodontic Treatment: A Clinical and Molecular Microbiological Study. Photochemistry and Photobiology. 2018;94(2):351-6.

76. Arruda MEF, Neves MAS, Diogenes A, Mdala I, Guilherme BPS, Siqueira JF, Jr., et al. Infection Control in Teeth with Apical Periodontitis Using a Triple Antibiotic Solution or Calcium Hydroxide with Chlorhexidine: A Randomized Clinical Trial. J Endod. 2018;44(10):1474-9.

77. Rodrigues RCV, Zandi H, Kristoffersen AK, Enersen M, Mdala I, Ørstavik D, et al. Influence of the Apical Preparation Size and the Irrigant Type on Bacterial Reduction in Root Canal-treated Teeth with Apical Periodontitis. J Endod. 2017;43(7):1058-63.

78. Rabello DGD, Corazza BJM, Ferreira LL, Santamaria MP, Gomes APM, Martinho FC. Does supplemental photodynamic therapy optimize the disinfection of bacteria and endotoxins in one-visit and two-visit root canal therapy? A randomized clinical trial. Photodiagnosis and Photodynamic Therapy. 2017;19:205-11.

79. Kist S, Kollmuss M, Jung J, Schubert S, Hickel R, Huth KC. Comparison of ozone gas and sodium hypochlorite/chlorhexidine two-visit disinfection protocols in treating apical periodontitis: a randomized controlled clinical trial. Clin Oral Investig. 2017;21(4):995-1005.

80. Cavalli D, Toia CC, Flores Orozco EI, Khoury RD, Cardoso F, Alves MC, et al. Effectiveness in the Removal of Endotoxins and Microbiological Profile in Primary Endodontic Infections Using 3 Different Instrumentation Systems: A Randomized Clinical Study. J Endod. 2017;43(8):1237-45.

81. Asnaashari M, Ashraf H, Rahmati A, Amini N. A comparison between effect of photodynamic therapy by LED and calcium hydroxide therapy for root canal disinfection against Enterococcus faecalis: A randomized controlled trial. Photodiagnosis and Photodynamic Therapy. 2016;17.

82. Zandi H, Rodrigues RC, Kristoffersen AK, Enersen M, Mdala I, Ørstavik D, et al. Antibacterial Effectiveness of 2 Root Canal Irrigants in Root-filled Teeth with Infection: A Randomized Clinical Trial. J Endod. 2016;42(9):1307-13.

83. Rôças IN, Provenzano JC, Neves MA, Siqueira JF, Jr. Disinfecting Effects of Rotary Instrumentation with Either 2.5% Sodium Hypochlorite or 2% Chlorhexidine as the Main Irrigant: A Randomized Clinical Study. J Endod. 2016;42(6):943-7.

84. Rico-Romano C, Zubizarreta-Macho Á, Baquero-Artigao MR, Mena-Álvarez J. An analysis in vivo of intracanal bacterial load before and after chemo-mechanical preparation: A comparative analysis of two irrigants and two activation techniques. J Clin Exp Dent. 2016;8(1):e9-e13.

85. Neves MA, Provenzano JC, Rôças IN, Siqueira JF, Jr. Clinical Antibacterial Effectiveness of Root Canal Preparation with Reciprocating Single-instrument or Continuously Rotating Multi-instrument Systems. J Endod. 2016;42(1):25-9.

86. Ghoneim M, Saber SE, El-Badry T, Obeid M, Hassib N. The Use of Different Irrigation Techniques to Decrease Bacterial Loads in Healthy and Diabetic Patients with Asymptomatic Apical Periodontitis. Open Access Maced J Med Sci. 2016;4(4):714-9.

87. Donyavi Z, Ghahari P, Esmaeilzadeh M, Kharazifard M, Yousefi-Mashouf R. Antibacterial Efficacy of Calcium Hydroxide and Chlorhexidine Mixture for Treatment of Teeth with Primary Endodontic Lesions: A Randomized Clinical Trial. Iran Endod J. 2016;11(4):255-60.

88. Barbosa-Ribeiro M, De-Jesus-Soares A, Zaia AA, Ferraz CC, Almeida JF, Gomes BP. Quantification of Lipoteichoic Acid Contents and Cultivable Bacteria at the Different Phases of the Endodontic Retreatment. J Endod. 2016;42(4):552-6.

89. Asnaashari M, Godiny M, Azari-Marhabi S, Tabatabaei FS, Barati M. Comparison of the Antibacterial Effect of 810 nm Diode Laser and Photodynamic Therapy in Reducing the Microbial Flora of Root Canal in Endodontic Retreatment in Patients With Periradicular Lesions. J Lasers Med Sci. 2016;7(2):99-104.

90. Dds E, Ballestero C, Esp E, Ramirez-Mora T, Dds E, Dds E, et al. Efficacy of Endodontic Treatment on the Persistence of Selected Endodontic Pathogens and on Radiographical Periapical Healing. Odovtos - International Journal of Dental Sciences. 2015;17:43.

91. Rodrigues RC, Antunes HS, Neves MA, Siqueira JF, Jr., Rôças IN. Infection Control in Retreatment Cases: In Vivo Antibacterial Effects of 2 Instrumentation Systems. J Endod. 2015;41(10):1600-5.

92. Provenzano JC, Rôças IN, Tavares LFD, Neves BC, Siqueira JF. Short-chain Fatty Acids in Infected Root Canals of Teeth with Apical Periodontitis before and after Treatment. Journal of Endodontics. 2015;41(6):831-5.

93. Martinho FC, Nascimento GG, Leite FR, Gomes AP, Freitas LF, Camões IC. Clinical influence of different intracanal medications on Th1-type and Th2-type cytokine responses in apical periodontitis. J Endod. 2015;41(2):169-75.

94. Martinho FC, Freitas LF, Nascimento GG, Fernandes AM, Leite FR, Gomes AP, et al. Endodontic retreatment: clinical comparison of reciprocating systems versus rotary system in disinfecting root canals. Clin Oral Investig. 2015;19(6):1411-7.

95. Marinho AC, Martinho FC, Leite FR, Nascimento GG, Gomes BP. Proinflammatory Activity of Primarily Infected Endodontic Content against Macrophages after Different Phases of the Root Canal Therapy. J Endod. 2015;41(6):817-23.

96. Ferreira NS, Martinho FC, Cardoso FG, Nascimento GG, Carvalho CA, Valera MC. Microbiological profile resistant to different intracanal medications in primary endodontic infections. J Endod. 2015;41(6):824-30.

97. Adl A, Motamedifar M, Shams MS, Mirzaie A. Clinical investigation of the effect of calcium hydroxide intracanal dressing on bacterial lipopolysaccharide reduction from infected root canals. Aust Endod J. 2015;41(1):12-6.

98. Teles AM, Conceição Manso M, Pina C, Cabeda J. In vivo evaluation of microbial reduction after chemo-mechanical preparation of necrotic root canals with or without apical periodontitis. Revista Portuguesa de Estomatologia, Medicina Dentária e Cirurgia Maxilofacial. 2014;55(2):89-96.

99. Stojanović N, Krunić J, Popović B, Stojičić S, Zivković S. Prevalence of Enterococcus faecalis and Porphyromonas gingivalis in infected root canals and their susceptibility to endodontic treatment procedures: a molecular study. Srp Arh Celok Lek. 2014;142(9-10):535-41.

100. Rôças IN, Neves MA, Provenzano JC, Siqueira JF, Jr. Susceptibility of as-yet-uncultivated and difficult-to-culture bacteria to chemomechanical procedures. J Endod. 2014;40(1):33-7.

101. Neves MA, Rôças IN, Siqueira JF, Jr. Clinical antibacterial effectiveness of the self-adjusting file system. Int Endod J. 2014;47(4):356-65.

102. Mashalkar S, Pawar MG, Kolhe S, Jain DT. Comparative evaluation of root canal disinfection by conventional method and laser: an in vivo study. Niger J Clin Pract. 2014;17(1):67-74.

103. Martinho F, Gomes AP, Fernandes A, Ferreira NDs, Endo M, Freitas L, et al. Clinical Comparison of the Effectiveness of Single-file Reciprocating Systems and Rotary Systems for Removal of Endotoxins and Cultivable Bacteria from Primarily Infected Root Canals. Journal of endodontics. 2014;40:625-9.

104. Jurič IB, Plečko V, Pandurić DG, Anić I. The antimicrobial effectiveness of photodynamic therapy used as an addition to the conventional endodontic re-treatment: a clinical study. Photodiagnosis Photodyn Ther. 2014;11(4):549-55.

105. Xavier AC, Martinho FC, Chung A, Oliveira LD, Jorge AO, Valera MC, et al. One-visit versus two-visit root canal treatment: effectiveness in the removal of endotoxins and cultivable bacteria. J Endod. 2013;39(8):959-64.

106. Rôças IN, Lima KC, Siqueira JF, Jr. Reduction in bacterial counts in infected root canals after rotary or hand nickel-titanium instrumentation--a clinical study. Int Endod J. 2013;46(7):681-7.

107. Provenzano JC, Siqueira JF, Jr., Rôças IN, Domingues RR, Paes Leme AF, Silva MR. Metaproteome analysis of endodontic infections in association with different clinical conditions. PLoS One. 2013;8(10):e76108.

108. Paiva SS, Siqueira JF, Jr., Rôças IN, Carmo FL, Leite DC, Ferreira DC, et al. Molecular microbiological evaluation of passive ultrasonic activation as a supplementary disinfecting step: a clinical study. J Endod. 2013;39(2):190-4.

109. Paiva SS, Siqueira JF, Jr., Rôças IN, Carmo FL, Leite DC, Ferreira DC, et al. Clinical antimicrobial efficacy of NiTi rotary instrumentation with NaOCl irrigation, final rinse with chlorhexidine and interappointment medication: a molecular study. Int Endod J. 2013;46(3):225-33.

110. Halbauer K, Prskalo K, Janković B, Tarle Z, Pandurić V, Kalenić S. Efficacy of ozone on microorganisms in the tooth root canal. Coll Antropol. 2013;37(1):101-7.

111. Endo MS, Ferraz CCR, Zaia AA, Almeida JFA, Gomes BPFA. Quantitative and qualitative analysis of microorganisms in root-filled teeth with persistent infection: Monitoring of the endodontic retreatment. Eur J Dent. 2013;07(03):302-9.

112. Cohenca N, Paranjpe A, Heilborn C, Johnson JD. Antimicrobial efficacy of two irrigation techniques in tapered and non-tapered canal preparations. A randomized controlled clinical trial. Quintessence Int. 2013;44(3):217-28.

113. Endo MS, Martinho FC, Zaia AA, Ferraz CC, Almeida JF, Gomes BP. Quantification of cultivable bacteria and endotoxin in post-treatment apical periodontitis before and after chemo-mechanical preparation. Eur J Clin Microbiol Infect Dis. 2012;31(10):2575-83.

114. Rôças IN, Siqueira JF, Jr. Comparison of the in vivo antimicrobial effectiveness of sodium hypochlorite and chlorhexidine used as root canal irrigants: a molecular microbiology study. J Endod. 2011;37(2):143-50.

115. Rôças IN, Siqueira JF, Jr. In vivo antimicrobial effects of endodontic treatment procedures as assessed by molecular microbiologic techniques. J Endod. 2011;37(3):304-10.

116. Martinho FC, Chiesa WMM, Marinho ACS, Zaia AA, Ferraz CCR, Almeida JFA, et al. Clinical Investigation of the Efficacy of Chemomechanical Preparation with Rotary Nickel-Titanium Files for Removal of Endotoxin from Primarily Infected Root Canals. Journal of Endodontics. 2010;36(11):1766-9.

117. Abbaszadegan A, Khayat A, Motamedifar M. Comparison of Antimicrobial Efficacy of IKI and NaOCl Irrigants in Infected Root Canals: An In Vivo Study. Iran Endod J. 2010;5(3):101-6.

118. Malkhassian G, Manzur AJ, Legner M, Fillery ED, Manek S, Basrani BR, et al. Antibacterial efficacy of MTAD final rinse and two percent chlorhexidine gel medication in teeth with apical periodontitis: a randomized double-blinded clinical trial. J Endod. 2009;35(11):1483-90.

119. Bebek B, Bago I, Skaljac G, Plecko V, Miletić I, Anić I. Antimicrobial effect of 0.2% chlorhexidine in infected root canals. Coll Antropol. 2009;33(4):1159-63.

120. Vianna ME, Horz HP, Conrads G, Feres M, Gomes BP. Comparative analysis of endodontic pathogens using checkerboard hybridization in relation to culture. Oral Microbiol Immunol. 2008;23(4):282-90.

121. Martinho FC, Gomes BP. Quantification of endotoxins and cultivable bacteria in root canal infection before and after chemomechanical preparation with 2.5% sodium hypochlorite. J Endod. 2008;34(3):268-72.

122. Garcez AS, Nuñez SC, Hamblin MR, Ribeiro MS. Antimicrobial effects of photodynamic therapy on patients with necrotic pulps and periapical lesion. J Endod. 2008;34(2):138-42.

123. Garcez AS, Núñez SC, Hamblin MR, Ribeiro MS. Antimicrobial comparison on effectiveness of endodontic therapy and endodontic therapy combined with photo-disinfection on patients with periapical lesion: a 6 month follow-up. SPIE Proceedings. 2008;6846:86-92.

124. Blome B, Braun A, Sobarzo V, Jepsen S. Molecular identification and quantification of bacteria from endodontic infections using real-time polymerase chain reaction. Oral Microbiol Immunol. 2008;23(5):384-90.

125. Wang CS, Arnold RR, Trope M, Teixeira FB. Clinical efficiency of 2% chlorhexidine gel in reducing intracanal bacteria. J Endod. 2007;33(11):1283-9.

126. Vianna ME, Horz HP, Conrads G, Zaia AA, Souza-Filho FJ, Gomes BP. Effect of root canal procedures on endotoxins and endodontic pathogens. Oral Microbiol Immunol. 2007;22(6):411-8.

127. Siqueira JF, Jr., Rôças IN, Paiva SS, Guimarães-Pinto T, Magalhães KM, Lima KC. Bacteriologic investigation of the effects of sodium hypochlorite and chlorhexidine during the endodontic treatment of teeth with apical periodontitis. Oral Surg Oral Med Oral Pathol Oral Radiol Endod. 2007;104(1):122-30.

128. Siqueira JF, Jr., Paiva SS, Rôças IN. Reduction in the cultivable bacterial populations in infected root canals by a chlorhexidine-based antimicrobial protocol. J Endod. 2007;33(5):541-7.

129. Siqueira JF, Jr., Magalhães KM, Rôças IN. Bacterial reduction in infected root canals treated with 2.5% NaOCl as an irrigant and calcium hydroxide/camphorated paramonochlorophenol paste as an intracanal dressing. J Endod. 2007;33(6):667-72.

130. Siqueira JF, Jr., Guimarães-Pinto T, Rôças IN. Effects of chemomechanical preparation with 2.5% sodium hypochlorite and intracanal medication with calcium hydroxide on cultivable bacteria in infected root canals. J Endod. 2007;33(7):800-5.

131. Schirrmeister JF, Liebenow AL, Braun G, Wittmer A, Hellwig E, Al-Ahmad A. Detection and eradication of microorganisms in root-filled teeth associated with periradicular lesions: an in vivo study. J Endod. 2007;33(5):536-40.

132. Sakamoto M, Siqueira JF, Jr., Rôças IN, Benno Y. Bacterial reduction and persistence after endodontic treatment procedures. Oral Microbiol Immunol. 2007;22(1):19-23.

133. Paquette L, Legner M, Fillery ED, Friedman S. Antibacterial Efficacy of Chlorhexidine Gluconate Intracanal Medication In Vivo. Journal of Endodontics. 2007;33(7):788-95.

134. Manzur A, González AM, Pozos A, Silva-Herzog D, Friedman S. Bacterial quantification in teeth with apical periodontitis related to instrumentation and different intracanal medications: a randomized clinical trial. J Endod. 2007;33(2):114-8.

135. Carver K, Nusstein J, Reader A, Beck M. In vivo antibacterial efficacy of ultrasound after hand and rotary instrumentation in human mandibular molars. J Endod. 2007;33(9):1038-43.

136. Vianna ME, Horz HP, Gomes BP, Conrads G. In vivo evaluation of microbial reduction after chemo-mechanical preparation of human root canals containing necrotic pulp tissue. Int Endod J. 2006;39(6):484-92.

137. Soares JA, Pires Júnior DR. Influence of sodium hypochlorite-based irrigants on the susceptibility of intracanal microbiota to biomechanical preparation. Braz Dent J. 2006;17(4):310-6.

138. Chu FC, Leung WK, Tsang PC, Chow TW, Samaranayake LP. Identification of cultivable microorganisms from root canals with apical periodontitis following two-visit endodontic treatment with antibiotics/steroid or calcium hydroxide dressings. J Endod. 2006;32(1):17-23.

139. McGurkin-Smith R, Trope M, Caplan D, Sigurdsson A. Reduction of Intracanal Bacteria Using GT Rotary Instrumentation, 5.25% NaOCl, EDTA, and Ca(OH)2. Journal of Endodontics. 2005;31(5):359-63.

140. Ferrari PH, Cai S, Bombana AC. Effect of endodontic procedures on enterococci, enteric bacteria and yeasts in primary endodontic infections. Int Endod J. 2005;38(6):372-80.

141. de Souza CA, Teles RP, Souto R, Chaves MA, Colombo AP. Endodontic therapy associated with calcium hydroxide as an intracanal dressing: microbiologic evaluation by the checkerboard DNA-DNA hybridization technique. J Endod. 2005;31(2):79-83.

142. Kvist T, Molander A, Dahlén G, Reit C. Microbiological evaluation of one- and two-visit endodontic treatment of teeth with apical periodontitis: a randomized, clinical trial. J Endod. 2004;30(8):572-6.

143. Ercan E, Ozekinci T, Atakul F, Gül K. Antibacterial activity of 2% chlorhexidine gluconate and 5.25% sodium hypochlorite in infected root canal: in vivo study. J Endod. 2004;30(2):84-7.

144. Peters LB, van Winkelhoff AJ, Buijs JF, Wesselink PR. Effects of instrumentation, irrigation and dressing with calcium hydroxide on infection in pulpless teeth with periapical bone lesions. Int Endod J. 2002;35(1):13-21.

145. Dostálová T, Jelínková H, Housová D, Sulc J, Nemeć M, Dusková J, et al. Endodontic treatment with application of Er:YAG laser waveguide radiation disinfection. J Clin Laser Med Surg. 2002;20(3):135-9.

146. Peciuliene V, Reynaud AH, Balciuniene I, Haapasalo M. Isolation of yeasts and enteric bacteria in root-filled teeth with chronic apical periodontitis. Int Endod J. 2001;34(6):429-34.

147. Shuping GB, Orstavik D, Sigurdsson A, Trope M. Reduction of intracanal bacteria using nickel-titanium rotary instrumentation and various medications. J Endod. 2000;26(12):751-5.

148. Dalton BC, Orstavik D, Phillips C, Pettiette M, Trope M. Bacterial reduction with nickel-titanium rotary instrumentation. J Endod. 1998;24(11):763-7.
